# Crosstalk among m^6^A RNA methylation, hypoxia and metabolic reprogramming in TME: from immunosuppressive microenvironment to clinical application

**DOI:** 10.1186/s13045-022-01304-5

**Published:** 2022-07-06

**Authors:** Fusheng Zhang, Haiyang Liu, Meiqi Duan, Guang Wang, Zhenghou Zhang, Yutian Wang, Yiping Qian, Zhi Yang, Xiaofeng Jiang

**Affiliations:** Department of General Surgery, The Fourth Affiliated Hospital of China Medical University, Shenyang, 110032 China

**Keywords:** Hypoxia, Tumor metabolism, Exosomes, Immune escape, Tumor biological functions, Tumor combination therapy

## Abstract

The tumor microenvironment (TME), which is regulated by intrinsic oncogenic mechanisms and epigenetic modifications, has become a research hotspot in recent years. Characteristic features of TME include hypoxia, metabolic dysregulation, and immunosuppression. One of the most common RNA modifications, N6-methyladenosine (m^6^A) methylation, is widely involved in the regulation of physiological and pathological processes, including tumor development. Compelling evidence indicates that m^6^A methylation regulates transcription and protein expression through shearing, export, translation, and processing, thereby participating in the dynamic evolution of TME. Specifically, m^6^A methylation-mediated adaptation to hypoxia, metabolic dysregulation, and phenotypic shift of immune cells synergistically promote the formation of an immunosuppressive TME that supports tumor proliferation and metastasis. In this review, we have focused on the involvement of m^6^A methylation in the dynamic evolution of tumor-adaptive TME and described the detailed mechanisms linking m^6^A methylation to change in tumor cell biological functions. In view of the collective data, we advocate treating TME as a complete ecosystem in which components crosstalk with each other to synergistically achieve tumor adaptive changes. Finally, we describe the potential utility of m^6^A methylation-targeted therapies and tumor immunotherapy in clinical applications and the challenges faced, with the aim of advancing m^6^A methylation research.

## Introduction

N6-methyladenosine, a methylation process occurring at the N6 position of adenosine, is one of the most abundant and conserved internal transcriptional modifications, especially in eukaryotic messenger RNA (mRNA) [1]. More than 7000 human genes with 12,000 m^6^A methylation sites that are enriched in the consensus sequence ‘RRACH’, which is predominantly located stop codons and 3′ untranslated regions (3′ UTRs) [2]. The m^6^A methylation process is regulated by multiple regulators that are classified into three types, specifically, methyltransferases (writers), demethylases (erasers), and reading proteins (readers), which, respectively, add, remove or read an m^6^A methylation site. Methyltransferase writers (METTL3, METTL14, METTL16, Wilms tumor 1-associated protein (WTAP), KIAA1429) are responsible for initiation of the m^6^A methylation modification process [3, 4]. The main function of reading proteins (YTHDF1/2/3 and YTHDC1/2 etc.) is to recognize bases that undergo m^6^A methylation, thus activating downstream regulatory pathways, such as RNA degradation and miRNA processing [5, 6]. As erasers, the demethylases fat mass- and obesity-associated protein (FTO) and ALKBH5 are responsible for demethylation modification of bases that have undergone m^6^A methylation. These regulators (for example, the YTHDC and YTHDF families) are essential for RNA metabolic processes, including RNA shearing, export, translation, and processing [3, 7–9]. Therefore, m^6^A methylation is widely involved in multiple physiological activities. The issue of whether m^6^A methylation exerts anti- or pro-cancer effects remains controversial. The inconsistent results obtained to date may be attributable to distinct characteristics as a result of complex crosstalk of TME and differential regulation of target genes by m^6^A methylation [10]. However, accumulating evidence suggests that m^6^A methylation promotes the development of multiple diseases, including cancer, under specific circumstances [11–13].

Tumor survival and proliferation cannot be achieved without the support of TME, a highly complex and heterogeneous ecosystem that includes not only the tumor cells themselves but also their surrounding cell milieu, including immunosuppressive cells (tumor-associated mesenchymal stem cells (TA-MSCs), cancer-associated fibroblasts (CAFs), myeloid-derived suppressor cells (MDSCs), tumor-associated macrophages (TAMs), immune and inflammatory cells, and various other cell types) as well as intercellular stroma, microvasculature, and biomolecules infiltrating from nearby regions [14, 15]. The interactions between tumor cells and TME promote proliferation, differentiation, invasion, metastasis and even drug resistance [16]. Multiple components of TME contribute to the formation of an immunosuppressive microenvironment that promotes tumor immune escape, thus accelerating progression of these events, which can be attributed to hypoxia, metabolic dysfunction, immune cell phenotypic shift, and tumor-derived exosomes (Tu-Exo) [17–20]. As the most prevalent epigenetic modification of mRNA and non-coding RNA [21], m^6^A methylation achieves post-transcriptional control of protein expression through effects on RNA metabolism, in turn, influencing a wide range of cellular activities [10]. The efficacy of tumor immunotherapy is significantly limited by the immunosuppressive TME. To overcome these barriers, systematic evaluation of the phenotypic and functional changes of immune cells in TME is crucial. Interestingly, increasing evidence suggests that m^6^A methylation has the potential to support tumor immune escape via modulation of the immunosuppressive TME [22–24]. Indeed, tumor cell proliferation leads to hypoxia, which promotes the onset of metabolic reprogramming and participates in the phenotypic and functional conversion of immune cells in a m^6^A methylation-dependent manner, leading to the formation of an immunosuppressive tumor microenvironment (TME) that facilitates changes in mutually supportive biological functions to stimulate distant tumor metastasis. This review focuses on the complex association of m^6^A methylation with TME, highlighting potential mechanisms by which m^6^A methylation contributes to tumor growth and metastasis, including regulation of the hypoxic environment, metabolic reprogramming, immune cells, and biological functions.

## m^6^A methylation and hypoxia in TME

Excessive distance between the vascular system and tumor cells can lead to diffusion-limited hypoxia [25]. Under conditions of excessive tissue hypoxia, homeostasis of the microenvironment is disrupted, generating a hypoxic, hypoglycemic, and acidic TME conducive to tumor growth [26]. Notably, hypoxia and tumor growth form a mutually positive feedback loop. Tumor cell proliferation leads to excessive oxygen depletion and promotes a hypoxic environment in TME, which, in turn, provides conditions suitable for tumorigenesis and metastasis through multiple modalities including proliferation, differentiation, and drug resistance [23, 27]. Cell response to hypoxia is mainly controlled by hypoxia-inducible factors (HIF), heterodimeric helix-loop-helix proteins composed of O_2_-labile alpha and constitutively expressed beta subunits (HIF1α, HIF2α, and HIF-1β), involved in coordinating the regulation of numerous mechanisms that enable tumor cells to adapt to the hostile environment [28]. HIF-1α and HIF-2α primarily recognize similar hypoxia response elements in promoters of target genes and function stably in hypoxic environments [29]. HIF activates genes that control cellular oxygen homeostasis, including those involved in glucose metabolism and lactate metabolism. These molecular changes lead to a shift toward glycolysis rather than oxidative metabolism to accomplish tumor adaptive changes [30].

### m^6^A methylation promotes tumor progression in hypoxic conditions

HIF is extensively involved in tumor metabolism and has been shown to play a pivotal role in immune escape. Numerous m^6^A modification-related enzymes participate in regulation of HIF methylation and expression, consequently affecting tumor progression [31]. The immunosuppressive TME is critical for tumor growth and paracrine mediators facilitate tumor evasion of immune surveillance. A number of researchers have proposed that hypoxia-induced ALKBH5 stabilizes transcripts and promotes relocalization of the transcriptional repressor, SFPQ, from the CXCL8 promoter to paraspeckles by eliminating the deposition of m^6^A-methylated lncRNA NEAT1, ultimately stimulating tumor macrophage recruitment and tumor immune escape through upregulation of CXCL8/IL8 in glioblastoma [32]. Interestingly, m^6^A methylation is involved in shaping the hypoxic, hypoglycemic, and acidic microenvironment and promotes tumor growth through metabolic reprogramming. For instance, upregulation of hepatitis B virus X-interacting protein (HBXIP) positively promotes expression of the m^6^A methylase METTL3, resulting in enhanced HIF-1α expression and maintenance of high levels of glycolysis, thus promoting malignant proliferation of hepatocellular carcinoma (HCC) [33]. Accordingly, we suggest that m^6^A methylation and hypoxia synergistically promote tumor proliferation via forming a positive feedback loop. Hypoxia-induced YTH-domain family 2 (YTHDF2) is reported to inhibit hepatocellular carcinoma (HCC) growth via destabilizing EGFR mRNA, which may contribute to resistance to tumorigenesis in a hypoxic environment [34]. However, hypoxia in TME mainly promotes tumorigenesis and malignant progression by altering m^6^A methylation levels to affect downstream adaptive responses, such as immune cell function and tumor biological behavior (including angiogenesis, autophagy, apoptosis, epithelial-mesenchymal transition (EMT), and distant metastasis [35]), as discussed in detail below.

### HIF affects immune cell function

Hypoxia-mediated HIF is proposed to regulate various aspects of tumor immunity, particularly immune cell populations critical for effective anti-tumor immune responses. Disruption of these cell populations impairs the immune response and contributes to the development of immunosuppressive TME, thus allowing tumors to evade immune surveillance and killing.

As a major cellular component of the adaptive immune response to tumor neoantigens, T lymphocyte proliferation and function are significantly suppressed in hypoxia compared to normoxic conditions. Hypoxic areas of spleen and lymph may block CD8^+^ T-cell activation by stabilizing HIF1α and inhibiting TCR-mediated Ca^2+^ signaling [36]. In hypoxia, differentiation of effector T cells is suppressed and induces a decrease in effector cytokines (such as IFN-γ, IL-2, and proliferative cytokines), facilitating the tumor escape from immune-mediated detection and killing [37]. B cells are an important component of the immune system, and HIF increases the rate of B cell glycolysis, thereby reducing their proliferation and increasing B cell apoptosis to weaken the immune response; this process is associated with c-Myc-dependent glycolysis, ROS-induced apoptosis, glucose limitation, and the hypoxic microenvironment [38, 39]. Natural killer (NK) cells are known for their ability to non-specifically kill tumor cells. Multiple lines of evidence suggest that hypoxia can suppress the killing function of NK cells, potentially through activation of the PI3K/mTOR signaling pathway that upregulates HIF expression resulting in inhibition of NK cell function [40]. Furthermore, HIF1-α induces regulatory T cell (Treg) infiltration, which activates the immunosuppressive factor TGF-β, thereby impeding the antitumor potential of NK cells [41].

Immunosuppressive cells are essential for tumor growth and development, including Tregs, CAFs, and TAMs. Treg infiltration in TME could enhance tumor progression via suppressing anti-tumor immunity and promoting tumor immune evasion. Mechanistically, HIF-1 stimulates FOXP3 transcription by binding the promoter region of FOXP3 expressed in CD4^+^ T cells in a TGF-β-dependent manner, ultimately inducing differentiation into Tregs [42]. Specific cytokines act in concert with Treg to generate an environment that supports tumor growth. In HCC, Treg recruitment can be achieved via HIF-1α-dependent upregulation of CCL28 [43]. CAFs are abundant in stromal cells of TME and contribute to tumorigenesis by influencing the paracrine release of cytokines through various immunomodulatory mechanisms to promote extracellular matrix remodeling [44]. Considerable evidence suggests that HIF-1α is upregulated in CAFs and its activation in hypoxia mediates TGF-β signaling, which promotes release of CXCL13 from CAFs, thereby accelerating malignant progression of prostate cancer [45]. CAFs in TME can produce tumor-associated cytokines, such as IL-6, NF-κB, and TGF-β2, which support their function by enhancing secretion of HIF-1α and forming a feedback loop facilitating tumor migration and invasion by shaping the immunosuppressive TME [46, 47]. Therefore, blockage of the secretion of tumor-associated cytokines from CAFs may enhance the therapeutic effect of immune checkpoint blockade therapy. Macrophages that infiltrate tumor tissue or accumulate in the solid tumor microenvironment are defined as TAMs. Similar to CAFs, TAMs play important roles in tumor progression, such as promoting immunosuppression and proliferation through secretion of cytokines, which are mediated by HIF [48]. Tumors release succinate into the TME and activate the succinate receptor, which triggers the PI3K/HIF-1α signaling axis to polarize macrophages to TAMs and promote cancer cell migration and invasion [49].

### Potential of hypoxia and m^6^A methylation to co-construct an immunosuppressive TME

Studies to date have established that hypoxia-mediated m^6^A methylation promotes tumorigenesis and HIF contributes to the formation of an immunosuppressive TME that provides suitable conditions for tumor proliferation. Our hypothesis that hypoxia-mediated m^6^A methylation weakens the anti-tumor capacity of immune cells and facilitates the formation of an immunosuppressive TME has been validated. For example, hypoxia-induced ALKBH5 eliminates deposition of the m^6^A methylated lncRNA, NEAT1, stabilizing transcription and promoting NEAT1-mediated paraspeckle assembly, thus leading to relocalization of SFPQ, a transcriptional repressor, from the CXCL8 promoter to paraspeckles and ultimately, TAM recruitment and immunosuppression via upregulation of CXCL8/IL8 [32]. Furthermore, hypoxia supports the m^6^A methylation process and tumor proliferation by increasing exosome release and altering its cargo, while tumor-derived exosomes can transform the immune cell phenotype to form suppressive TME (discussed in detail in section 4.3) [50], thus future research is warranted to establish the interrelationships among hypoxia, m^6^A methylation, exosomes and immunosuppressive TME in more detail, which would not only improve our understanding of the tumor epigenome (including the m^6^A methylation signaling network) but also help to identify novel anti-cancer targets.

## m^6^A methylation and metabolic reprogramming in TME

Over the past few decades, the medical consensus has been that the majority of tumors are genetically related diseases and caused by genetic mutations of different causes. Increasing knowledge of tumor phenotypes supports two typical features of tumors: energy metabolic reprogramming and evasion of immune surveillance [51]. Maintenance of cancer cell viability and function as well as evasion of immune recognition depends on metabolic reprogramming [23], leading to the proposal that cancer is essentially an immune-related metabolic disease. The metabolic program of tumor cells provides a hypoxic, hypoglycemic, and acidic tumor microenvironment that supports proliferation. Meanwhile, hypoxia-induced HIF significantly enhances glycolysis and lipid metabolism that promote cancer-induced metabolic disorders, creating a positive feedback loop to accelerate tumor progression [16]. Therefore, hypoxia and metabolic reprogramming can be considered interdependent and synergistic factors and cooperate in the generation of an immunosuppressive TME to achieve adaptive changes required for tumor progression (Table 1).

**Table 1:** Involvement of m^6^A methylation in regulation of tumor proliferation through TME

TME	m^6^A regulator	Tumor type	Mechanism	Effect on tumor progression	References
Hypoxia	METTL3	HCC	Methylates FOXO3	RNA m^6^A methylation regulates sorafenib resistance in liver cancer through FOXO3-mediated autophagy	[202]
Hypoxia	ALKBH5	Breast cancer	Hypoxia mediates NANOG mRNA m^6^A-demethylation through HIF dependence and ALKBH5	Induces breast cancer stem cell phenotype and accelerates tumor cell proliferation	[166]
Hypoxia	METTL3	HCC	Induces upregulation of HIF-1α and maintenance of higher levels of glycolysis in hypoxia by positively promoting expression of the m^6^A methyl esterase METTL3	Promotes malignant biological behavior in hepatocellular carcinoma	[33]
Hypoxia	YTHDF2	Lung cancer	Hypoxia-induced SUMOylation of YTHDF2 at the major site of K571 significantly enhances its binding affinity to m^6^A methylation-modified mRNA, leading to dysregulation of gene	Promoting the progression of lung cancer	[307]
Hypoxia	ALKBH5	Glioblastoma	Hypoxia-induced ALKBH5 eliminates m^6^A methylated lncRNA NEAT1 deposition, stabilizes transcripts and promotes NEAT1-mediated paraspeckle assembly, resulting in secretion of the immunosuppressive factor CXCL8/IL8	Shapes the immunosuppressive TME through TAM recruitment and supports immune escape in glioblastoma	[32]
Metabolic	FTO	HCC	FTO triggers the demethylation of PKM2 mRNA and accelerates translation	Promotes hepatocellular carcinoma	[62]
Metabolic	IGF2BP2	Colorectal cancer	Overexpression of the m^6^A methylation reader IGF2BP2 stabilizes the ZFAS1/OLA1 axis and increases OLA1 recruitment, ATP hydrolysis and glycolysis	Promotes colorectal cancer cell invasion and colony formation by activating the Warburg effect	[65]
Metabolic	METTL1	HCC	Upregulates PTEN/AKT signaling	Promotes HCC growth, resulting in poor prognosis	[308]
Metabolic	METTL3	Uveal melanoma	Upregulates c-Met, p-AKT, cyclin and CDK	Promotes tumor cell metastasis and invasion	[309]
Metabolic	YTHDF2	Prostate cancer	Binds to LHPP and NKX3-1	Promotes tumor growth	[310]
Metabolic	ALKBH5	Ovarian cancer	Activates EGFR-PIK3CA-AKT-mTOR	Promotes tumor proliferation	[311]
Metabolic	METTL3/YTHDF2	NA	METTL3 promotes YTHDF2 binding to PPaRα through m^6^A modification to increase its mRNA stability	Increases lipid accumulation in cells	[85]
Immune escape	ALKBH5	Pancreatic cancer	Modulates CD8^+^ and CD4^+^ T cells aggregation	Induction of tumor immune escape	[312]
Immune escape	ALKBH5	Melanoma	Affects the expression of Mct4/Slc16a3 in TME to regulate the composition of tumor-infiltrating Tregs and MDSCs	Promotes melanoma escape by enhancing immunosuppressive effects	[157]
Immune escape	YTHDF1	Colon cancer	YTHDF1 induces lysosomal protease expression by recognizing its m^6^A methylation-tagged mRNA	Resulting in the inability of DCs to consistently induce tumor neoantigen production and impeding antigen-specific activation of CD8^+^ T cells	[123]
Immune escape	YTHDF2	NA	YTHDF2 exacerbates the CCR7‐induced DCs migration process and completely disables DCs by alleviating m^6^A modification‐based RNA degradation of lnc‐Dpf3	Promotes immune escape	[125]
Immune escape	FTO	Melanoma	FTO-mediated m^6^A demethylation in tumor cells enhances transcription factors c-Jun, JunB and C/EBPβ, thereby inducing hypoxia-mediated glycolytic metabolism and suppressing CD8^+^ T cell function	Tumors exploit FTO-mediated regulation of glycolytic metabolism to evade immune surveillance	[129]
Immune escape	METTL3/IGF2BP3	Breast cancer	METTL3/IGF2BP3 axis upregulates m^6^A modification of PD-L1 mRNA, suppressing T cell activation in breast cancer	Promotes tumor immune escape	[130]
Immune escape	ALKBH5	NA	Deletion of ALKBH5 in T cells reduces mRNA stability and corresponding protein expression	Reduces neutrophil recruitment to the central nervous system during neuroinflammation resulting in impaired CD4^+^ T cell responses	[131]
Immune escape	METTL3	NA	Downregulation of METTL3 leads to reduced METTL3-mediated methylation of m^6^A targeting the IL-7/STAT5/SOCS pathway	Causes stagnation of T-cell development	[132]
Immune escape	YTHDF2	Lung cancer	NPM1 negatively regulates the growth and development of B and NK cells through glycolysis and YTHDF2-mediated methylation	Participates in immune infiltration of lung cancer and promotes immune tumor escape	[134]
Immune escape	METTL14	Colorectal cancer	METTL14 in TAM induces CD8^+^ T cell dysfunction	Promotes tumor progression	[149]
Immune escape	ALKBH5	Melanoma	ALKBH5 regulates the composition of tumor-infiltrating Tregs and MDSCs by affecting Mct4/Slc16a3 expression in TME	Achieves enhanced immunosuppressive effects and promotes immune escape of melanoma	[157]
Immune escape	METTL3	Melanoma	METTL3 deficiency impairs YTHDF1-mediated SPRED2 translation, in turn, enhancing NF-κB and STAT3 activation via the ERK pathway	Causes increased TAM-induced Treg infiltration into TME and supports tumor metastasis	[150]
Immune escape	WTAP	Gastric cancer	Elevated expression of WTAP affects tumor-associated T-lymphocyte infiltration	Causes poor prognosis of gastric cancer	[313]
Immune escape	METTL3	Testicular germ cell tumors	Expression of METTL3 is positively correlated with infiltration of CD8^+^ and CD4^+^ T cells	Exerts an appropriate anti-tumor immune response	[314]
Immune escape	YTHDF2/METTL3	Melanoma/ovarian cancer	YTHDF2/METTL3 maintain NK cell homeostasis and terminal maturation and promote NK cell function	Enhances anti-tumor immunity	[136, 138]
Exosomes	ALKBH5	Glioblastoma	Warburg effect promotes exosome circ_0072083 release to upregulate NANOG and ALKBH5 expression through multiple pathways	Enhances resistance to temozolomide in gliomas	[215]
Exosomes	METTL3	Lung cancer	Exosome miR-4443 regulates FSP1 m^6^A methylation-mediated ferroptosis	Promotes cisplatin resistance in lung cancer	[315]
Exosomes	METTL7A	Myeloma	Induction of m^6^A methylation in adipocyte exosomal lncRNAs to mediate myeloma drug resistance	Promotes drug resistance in myeloma and supports tumor growth	[185]
Exosomes	METTL3	Colorectal cancer	m^6^A methylation-dependent promotion of miR-181d-5p expression in cancer-associated fibroblast exosomes	Targeting NCALD inhibits 5-FU sensitivity in colorectal cancer	[176]

### Glucose metabolism

Abnormal glucose metabolism is the main feature of metabolic reprogramming in cancer cells. Energy metabolism characteristics that serve as a marker of high aggressiveness of tumor cells mainly include increased glycolytic activity and lactic acid fermentation [52]. Even in the presence of sufficient oxygen, tumor cells can undergo metabolic reprogramming that drives conversion of glucose to lactate (Warburg effect) [52]. Hypoxia induces a metabolic shift from oxidative phosphorylation to glycolysis and increases glycogen synthesis to facilitate tumor proliferation [53]. In cases where the level of oxygen does not meet tumor demand, tumor cells autonomously alter their flux through various metabolic pathways to meet increased bioenergetic and biosynthetic requirements and mitigate oxidative stress required for proliferation and survival [54]. The cellular response to such complex microenvironmental changes is regulated by multiple mechanisms. m^6^A methylation in TME leads to increased glycolysis and reduced mitochondrial function, thereby shifting energy production from mitochondria to glycolytic sources [55]. Interestingly, preferential activation of hypoxia-responsive glycolytic genes can be achieved via binding to m^6^A methylation regulators. Genes controlling extracellular glucose input (GLUT1) [56] and enzymes responsible for intracellular glycolytic breakdown of glucose (phosphofructokinase-1 (PFK1) and aldolase) are regulated by activated m^6^A methylation to induce glycolytic energy production [57]. These metabolic shifts shape the hypoxic, hypoglycemic, and acidic TME that facilitates tumor growth in multiple dimensions.

Several studies have demonstrated that m^6^A modulators (e.g., METTL3 and WTAP) affect tumor glucose metabolism by regulating glycolytic enzymes (Warburg effect) to promote proliferation [58–60]. The glycolytic pyruvate kinase isoenzyme PKM2 (a glycolytic rate-limiting enzyme) is expressed in a variety of cells, particularly tumor cells [61]. The m^6^A eraser, FTO, accelerates translation and promotes malignant biological behavior of HCC by triggering demethylation of PKM2 mRNA. Moreover, knockdown of FTO retards tumor growth via induction of G0/G1 phase block [62]. c-Myc is a core regulator of glycolysis [63]. WNT/β-linked protein promotes m^6^A modification of c-Myc mRNA and supports tumor cell glycolysis and progression by inhibiting expression of FTO, a m^6^A demethylase [64]. Further studies revealed that the key regulatory network of the m^6^A methylation reader, IGF2BP2, stabilizes lncRNA and collectively contributes to mitochondrial energy metabolism in tumor pathogenesis. Specifically, metastasis and colony formation of colorectal cancer cells can be accomplished by upregulating IGF2BP2 to stabilize the ZFAS1/OLA1 axis, leading to increased ATP hydrolysis and glycolysis and activation of the Warburg effect [65]. Therefore, blockage of m^6^A methylation-dependent glycolysis may open up new avenues for anti-tumor strategies. However, m^6^A methylation modifiers act as a double-edged sword in the regulation of cellular processes associated with glycolysis, which may depend on the large number of RNA-binding proteins (RBPs), including both m^6^A methylation reading and non-reading proteins, and their recognition sites. Huang et al. [66] suggested that FTO suppresses APOE through IGF2BP2-mediated m^6^A methylation and inhibits glycolytic metabolism in PTC through regulating the IL-6/JAK2/STAT3 signaling pathway, thereby retarding thyroid cancer cell growth. Correspondingly, YTHDC1-mediated enhancement of miR-30d suppressed pancreatic tumorigenesis via attenuation of RUNX1-induced transcriptional activation of the Warburg effect [67]. Notably, metabolic programming can affect TME via modulation of the m^6^A modification process. One theory is that sphingolipids generated from sequential ceramide glycosylation activate cSrc and β-linked protein signaling, thereby upregulating METTL3 and mutant p53 R273H protein expression that promote tumor drug resistance [68]. Evidently, m^6^A methylation is a complex process and plays multiple roles in tumor metabolism, which may be attributed to differences in the types of m^6^A methylation regulators and their recognition sites. Clearly, while m^6^A modifications regulate metabolic programming, metabolism also supports tumor development by influencing the m^6^A modification process [68].

TME is a complex and dynamically changing ecosystem in which the components and metabolic processes are interrelated. Hypoxia causes metabolic disturbances that support tumor progression [69]. According to recent literature, HBXIP drives the metabolic reprogramming of HCC through METTL3-mediated m^6^A methylation of HIF-1α, which stimulates the Warburg effect and tumor cell survival [33]. Correspondingly, with the involvement of HIF-1α, follicular helper T cell differentiation may be promoted by the E3 ubiquitin ligase VHL via m^6^A methylation-mediated glycolytic modification [70], which supports the formation of germinal centers and malignant tumors, such as follicular lymphoma [71]. As an important component of TME, exosomes (to be mentioned later) are synergistically involved in the construction of pre-metastatic ecological niches (PMN) to facilitate tumor metastasis. For instance, miR-122 in breast cancer-derived exosomes inhibits glucose uptake in recipient cells by downregulating the glycolytic enzymes pyruvate-kinase-2 (PKM2) and GLUT1, inducing nutrient competition in favor of tumor cells and promoting migration [72]. Similarly, Tu-Exo-contained miR-105 could induce MYC-dependent metabolic programs in CAFs. Glucose and glutamine metabolism were enhanced in these reprogrammed CAFs to provide energy to neighboring tumor cells [73]. Therefore, interactions among hypoxia, exosome, glucose metabolic and m^6^A methylation exist that contribute to the generation of a microenvironment conducive to tumor development.

### Lipid metabolism

Lipids are hydrophobic molecules that include sterols, glycerol monoesters, diacylglycerides, triglycerides, phospholipids, and glycolipids. Digestion, absorption, synthesis, and breakdown of lipid metabolism through the actions of multiple enzymes are necessary to maintain cellular homeostasis [23]. Cancer cells regulate and utilize lipid metabolism to support their own proliferation and metastasis to meet the high nutrient demand. In addition, lipid production provides material and energy sources for tumor proliferation [74], a typical example being fatty acid oxidation. Activation of fatty acid synthase is induced by lipid transformation to support tumor cell survival and invasion [75]. The available data clearly indicate that m^6^A modifications regulate lipid metabolism to support tumor progression [76]. For instance, YTHDF2 supports glioblastoma growth by inducing cholesterol dysregulation [77].

FTO-dependent m^6^A demethylation is intimately involved in multiple aspects of lipid metabolism. Interestingly, overexpression of FTO in HepG2 cells has been shown to enhance the expression of genes involved in lipid metabolism (FASN, SCD1) and downregulate lipid transport genes (MTTP, APOB), thereby causing lipid accumulation and conditions supporting the development of various diseases, including cancer [78]. Furthermore, FTO overexpression enhances the levels of the key regulators of lipogenesis (SREBP1c, CIDEC) in hepatocytes and accelerates lipid accumulation [79]. Therefore, silencing of FTO expression may present a potential therapeutic strategy. Similarly, the m^6^A methylation reader, HNRNPA2B1, upregulates fatty acid metabolism-related genes, ACLY and ACC1, which contribute to tumor growth and metastasis, by promoting lipid accumulation in cells [80]. Conclusive evidence indicates that hypoxia-induced m^6^A methylation provides nutritional and environmental support for tumor growth by regulating glucose metabolism [81, 82]. Accordingly, we speculate that similar situations may arise in lipid metabolism. Expression of fatty acid binding protein-5 (FABP5) is regulated by FTO in liver tissues [83], and it is proposed that FABP5 could enhance HIF-1α activity by disrupting FIH/HIF-1α interactions when promoting HIF-1α synthesis, thereby activating the FABP5/HIF-1α axis to support lipid accumulation in the liver and facilitate HCC proliferation [84]. However, these inferences require further research. Furthermore, METTL3 is reported to promote YTHDF2 binding to PPARα through m^6^A modifications that mediate its mRNA stability and enhance lipid accumulation in cells, suggesting that m^6^A RNA methylation regulates metabolic processes by affecting downstream genes and circadian rhythms of lipid metabolism [85].

### Amino acid metabolism

Tumor cells have increased demand for amino acids to meet their rapid proliferation requirements. Metabolism of amino acids in the body is mainly manifested as synthesis of nitrogenous substances such as proteins and peptides required for their own synthesis, while catabolism of amino acids occurs through deamination and transamination to produce metabolites, such as α-keto acids and CO_2_. Glutamine, a highly abundant amino acid in the human circulation, is catabolized to glutamate, internalized into mitochondria, converted to α-ketoglutarate, which enters the tricarboxylic acid (TCA) cycle to fuel the production of energy and intermediates [86]. TCA plays an irreplaceable role in multiple metabolic pathways and its functional maintenance in tumor cells is dependent on elevation of glutaminase. Therefore, among the several factors closely associated with tumor progression, one important feature of tumor energy metabolism is amino acid degradation [87]. For example, m^6^A modification-mediated upregulation of DEGS2 in kidney cancer inhibits ceramide synthesis to increase the invasive ability of cancer cells [88]

Under hypoxic conditions, glutamine consumption in tumor cells is increased and preferentially provides carbon for fatty acid synthesis through reductive carboxylation, where glutamine-derived α-ketoglutarate is reduced to citrate by isocitrate dehydrogenase and NADPH is oxidized to NADP^+^ to provide energy for tumor cell growth [89]. In renal clear cell carcinoma, loss of Von Hippel-Lindau (VHL) tumor suppressor function that leads to a significant increase in HIF activity is a critical indicator [90]. Synthetic lethal effects of FTO and VHL, structural activation of VEGF and PDGF induced by VHL inactivation, and targeting of VEGF and PDGF to the downstream glutamine transporter SLC1A5 promote VHL deficiency-mediated metabolic reprogramming in kidney cancer cells and selectively affect the proliferation of VHL-deficient kidney cancer cells [91]. This signaling pathway can transmit molecular signals from outside the cell through the membrane to within the cell, also observed in m^6^A methylation-regulated amino acid metabolism [92, 93]. A novel hypoxic lncRNA, KB-1980E6.3, encodes RNA-binding regulatory peptide, which recruits the m^6^A methylation reader, IGF2BP1, and promotes its binding to m^6^A methylation-modified c-Myc coding region instability cluster (CRD) mRNA via a KB-1980E6.3/IGF2BP1/c-Myc signaling axis to maintain c-Myc mRNA stability and augment tumorigenesis [94]. In another interesting experiment, targeting YTHDF1 effectively re-sensitizes cisplatin-resistant colon cancer by modulating GLS-mediated glutamine metabolism, providing a novel strategy for targeted glutamine therapy for cancer [95].

### Mitochondrial metabolism

Considering the key role of mitochondria as cellular energy factories and metabolic centers, abnormalities in mitochondrial metabolism support tumor proliferation and metastasis. Tumor cell stemness is maintained via oxidative phosphorylation and mitochondria-dependent energy synthesis [96]. A mitochondrial enzyme, methylenetetrahydrofolate dehydrogenase-2 (MTHFD2), involved in HIF-2α transcriptomic regulation has been shown to promote progression of kidney cancer [97]. MTHFD2 is not considered an m^6^A methyltransferase but is overexpressed in renal cancer and involved in regulation of m^6^A methylation. In particular, MTHFD2 promotes translation of HIF-2α via m^6^A methylation, in turn, stimulating aerobic glycolysis and cancer cell progression. MTHFD2 increases METTL3-dependent methylation levels of HIF-2α, which, in turn, binds the promoter region of the MTHFD2 gene. Its overexpression induces an increase in MTHFD2 levels, resulting in the formation of a positive feedback loop and enhanced tumor proliferation through mitochondrial metabolism [97]. Thus, in mitochondria, hypoxia and metabolic reprogramming may synergistically mediate the malignant biological behavior of tumor cells through m^6^A methylation. Moreover, metabolites generated by mitochondrial dysfunction inhibit VHL-dependent HIF-2α degradation, creating a pseudo-hypoxic state. However, in VHL-deficient cells with high FTO expression, PGC-1α expression is induced via a decrease in m^6^A methylation, which restores mitochondrial activity and promotes oxidative stress (OS) and ROS production, with consequent inhibition of tumor progression [98]. An earlier study by Duan et al. [23] reported that m^6^A modifications regulate tumor progression through effects on multiple signaling pathways, including mTOR, MAPK, PI3K-Akt, Wnt, and NF-κB. Furthermore, multiple metabolic pathways may be interconnected, with FTO-induced downregulation of m^6^A levels leading to inhibition of mitochondrial metabolism, which would promote lipid accumulation and provide potential for dysregulated lipid metabolism-mediated tumorigenesis [99].

### Glycan metabolism and vitamin metabolism

Additionally, m^6^A methylation regulates the progression of many diseases through vitamin metabolism (vitamin B12/C/D, etc.) [100–102]. Vitamin B12 deficiency dysregulates m^6^A mRNA methylation of genes associated with neurological function, such as cognitive dysfunction, mental retardation, or memory impairment [100]. In addition, m^6^A methylation is also involved in peritoneal injury through vitamin metabolism (vitamin D) [102]. However, little literature is available on the role of m^6^A methylation with vitamin metabolism in tumors. For glycan metabolism, multiple m^6^A methylation regulators (METTL13/IGF2BP2/YTHDF2, etc.) regulate the development of multiple diseases (e.g., tumor and kidney injury) through glycan metabolism. [103, 104], and these pathogenic mechanisms involve different signaling pathways (GLUT1-mTORC1 axis, TRAF6/NF-κB and NF-κB/MAPK pathways). For example, METTL3 promotes colorectal cancer by activating the m6A-GLUT1-mTORC1 axis [104]; YTHDF2 mediates lipopolysaccharide-induced osteoclastogenesis and inflammatory response via the NF-κB and MAPK signaling pathways [105]. However, the impact of glycan metabolism on tumor biological functions needs to be further explored in the future.

### Metabolites shape the immunosuppressive TME

Mounting evidence suggests that intermediates or products of tumor metabolism regulate the proliferation, differentiation, activation, and function of immune cells. Metabolic programs can produce toxic metabolites that reshape the microenvironment to promote tumor survival, such as 2-hydroxyglutarate (2-HG), lactic acid, and H^+^. Metabolites can also mediate immunosuppressive functions through m^6^A methylation. For instance, lactic acid-driven METTL3 mediates RNA m^6^A modifications that promote immunosuppressive capacity [106].

Tumors consume glucose and produce lactic acid, even in the presence of sufficient oxygen. Lactic acid accumulates in the extracellular environment and contributes to the formation of an acidic TME by activating monocarboxylate transport proteins in the cell membrane. Both lactate accumulation and TME acidification have profound effects on T cell-mediated antitumor immune responses [107]. Tumor-derived lactate promotes Treg activity in highly glycolytic TME and impairs CD8^+^ T cell function [108]. Indeed, tumor glycolysis-derived lactate accelerates tumor proliferation by promoting secretion of IL-23 and IL-17 [109]. Lactic acid causes functional changes in NK cells, intake of pathological concentrations of lactic acid leads to intracellular acidification of NK cells, which inhibits upregulation of nuclear factor of activated T cells and leads to decreased IFN-γ production and selective apoptosis of T and NK cells, resulting in accelerated tumor immune escape [110]. In TME, lactic acid alters the TAM phenotype to acquire tumor growth-enhancing properties, lactate inhibits TFEB-mediated expression of the macrophage-specific vesicular ATPase subunit, ATP6V0d2, by activating mTORC1, resulting in HIF-2α lysosomal degradation and programming of TAMs to a tumor growth-promoting immunophenotype [111]. Moreover, lactic acid in highly glycolytic TME enhances Treg cell function via upregulating PD-1 while suppressing effector T cell function, which underlies treatment failure [108].

Under nutrient-deficient conditions, nutrient acquisition by tumor cells can be achieved by scavenging extracellular proteins and degrading them to amino acids, including glutamine [112]. Besides cancer cells, glutamine utilization in immune cells reflects cell fate decisions and immune responses, such as activation of macrophages and neutrophils [113, 114]. Selective death of immune cells is significantly decreased with increasing glutamine utilization. Glutamine-mediated downregulation of the pro-apoptotic proteins, Bax and Bcl-xs, leads to increased survival of neutrophils [115]. Moreover, glutamine affects the secretion of pro-inflammatory cytokines by macrophages. In renal cancer, glutamine depletion leads to activation of HIF-1α and promotes secretion of IL-23 by tumor-infiltrating macrophages to activate regulatory T cell proliferation and enhance expression of IL-10 and transforming growth factor β, thereby inhibiting tumor cell killing by cytotoxic lymphocytes [116]. This finding confirms our conclusion (section 3.3) that glutamine degradation is a feature of cancer metabolism as a functional sustainer of tumor cells. Prostate cancer cells are radiosensitized by glutamine deprivation, which induces DNA damage, oxidative stress, epigenetic modifications, and tumor stem cell depletion [117]. Conversely, prostate cancer cells resistant to glutamine depletion display activation of autophagy-related gene-mediated macroautophagy/autophagy as a survival strategy against radiation-induced damage [117]. However, owing to tumor heterogeneity, the effects of glutamine in each tumor type need to be analyzed.

Lipid metabolites and key enzymes of the tricarboxylic acid cycle, such as isocitrate dehydrogenase (IDH), additionally have similar immunosuppressive functions. Indeed, abnormal accumulation of lipid metabolites in tumor-infiltrating myeloid cells has been shown to mediate immune reprogramming and contribute to conversion of immune cells into immunosuppressive and anti-inflammatory phenotypes [118]. Furthermore, mutations in IDH promote derivation of the oncogenic metabolite, 2-HG, which limits the production of chemokines, CXCL9 and CXCL10, by downregulating STAT1, resulting in reduced infiltration of CD8^+^ T cells and tumor escape [119]. Notably, tumor cell-derived metabolites have a non-negligible impact on immune cells in TME. The number of tumor metabolites and their effects on immune cells are too diverse for exhaustive explanation. Here, we have focused on the regulation of immune cells by lactate and glutamine. The immune regulation effects of other metabolites require considerable study. Collectively, the data suggest that m^6^A methylation elicits tumor metabolic reprogramming and affects immune cell functions and phenotypic shifts through supplying metabolites to generate an immunosuppressive TME, thus contributing to metastasis and invasion.

## m^6^A methylation remodels immunosuppressive TME by directly affecting immune cells

**Fig. 1:**
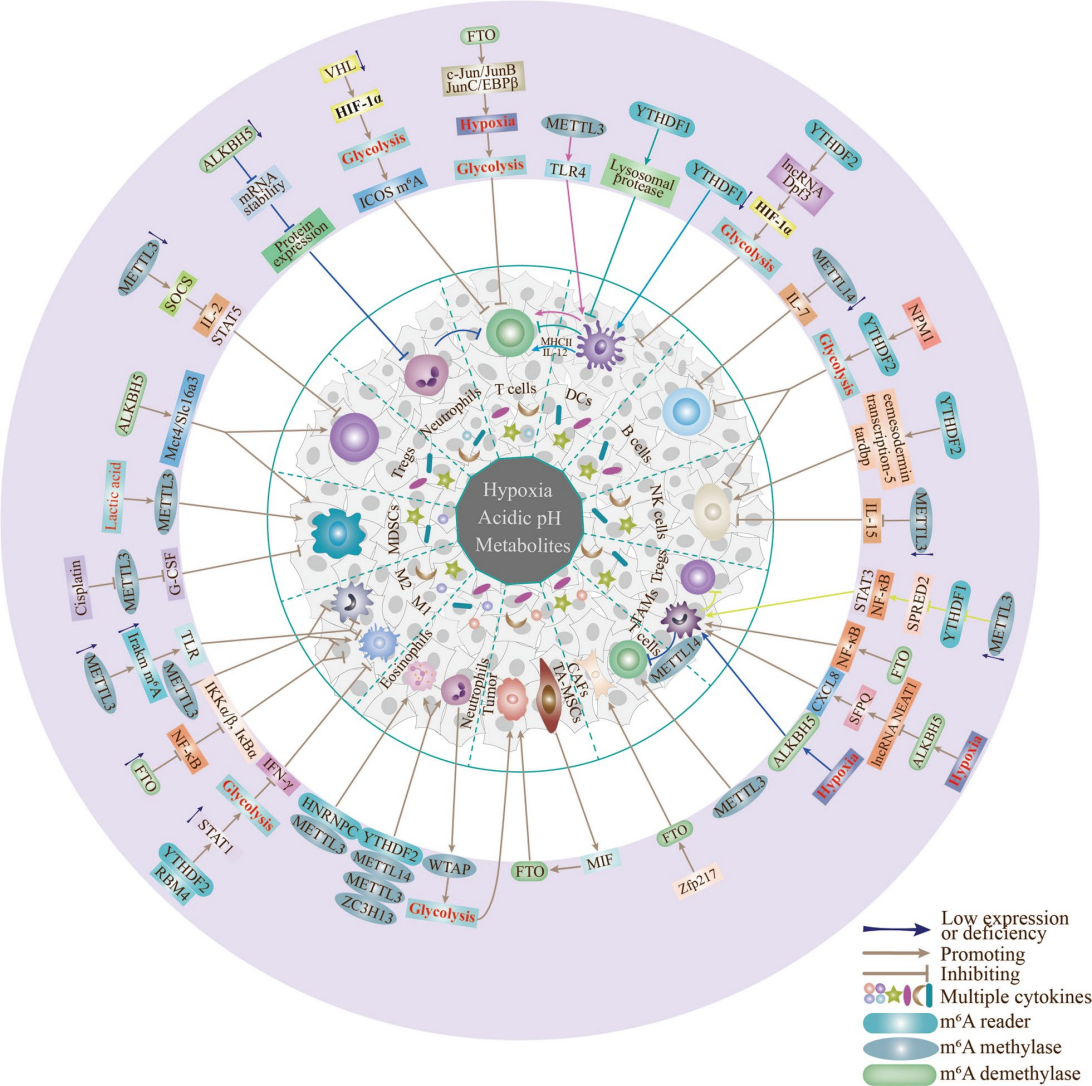
Effects of m^6^A modification on immune cells in hypoxia and metabolic TME. The different colored lines represent interactions of m^6^A modifications in immune cells through the respective pathways

Dysregulation of m^6^A methylation is closely related to cancer development and pathogenesis [120]. The majority of current studies have focused on immune regulation of tumors, with the key aim of addressing the persistent immunosuppressive response in TME [121]. However, the detailed mechanisms underlying the contributory effects of m^6^A methylation in immune cells to the series of transformations that culminate in "tumor immunity" are yet to be established (the formation of immunosuppressive TME). Here, we provide minimal representative but not exhaustive examples of some aspects of association of m^6^A methylation with tumor cell functions (Fig. 1).

### m^6^A methylation and anti-tumor immune cells in TME

#### Dendritic cells

Substantial abnormalities of m^6^A methylation have been reported in immune cells, such as dendritic cells (DCs), in TME. In certain cases, m^6^A methylation of mRNA expression significantly promotes immune dysfunction and tumor escape, in part, by inhibiting immune cell function, rather than suppression of cell proliferation and differentiation [122]. For example, YTHDF1 induces lysosomal protease expression by recognizing its m^6^A methylation-tagged mRNA and enhancing translation efficiency, further resulting in the inability of DCs to consistently induce tumor neoantigen production and impeding antigen-specific activation of CD8^+^ T cells [123]. Conversely, YTHDF1 deficiency in gastric cancer promotes the recruitment of mature DCs, which further stimulate MHCII expression and IL-12 secretion, in turn, increasing CD4^+^ and CD8^+^ T cell infiltration and IFN-γ secretion that potentially contribute to restoration of tumor immune sensitivity [124]. Therefore, YTHDF1 deletion may enhance antitumor immunity by facilitating interactions with DCs, rather than promoting their differentiation and proliferation, supporting a potential role of YTHDF2 as a tumor immunosuppressive factor. m^6^A modifications are also implicated in CC-chemokine receptor-7 (CCR7)-mediated migration of DCs and DC-based immune response pathways. The lncRNA Dpf3 plays a key role in these pathways, directly promoting HIF-1α activity and HIF-1α-dependent glycolytic metabolism to ultimately inhibit DC migration and inflammatory responses. YTHDF2 further exacerbates CCR7‐induced DC migration and completely disables DCs by alleviating m^6^A modification‐based RNA degradation of Dpf3 [125]. Therefore, induction of checkpoint blockade of YTHDF1 or YTHDF2 depletion in DCs may be effectively utilized as a potential immunotherapeutic strategy. Correspondingly, DC activation and function are influenced by METTL3-mediated methylation [126]. Specific depletion of METTL3 leads to impaired phenotypic and functional maturation of DCs and reduced expression of co-stimulatory molecules CD40, CD80 and cytokine IL-12 involved in maturation. Silencing of METTL3 has been shown to reduce the ability of DCs to stimulate T-cell responses [24]. Moreover, METTL3-mediated methylation of CD40, CD80 and TLR4 signal transduction junction TIRAP transcripts promotes translation in DCs to stimulate T-cell activation and enhances TLR4/NF-κB signaling to promote cytokine production [126]. Given the functional differences between METTL3 and YTHDF1/2 in DCs, this finding may be attributed to the involvement of m^6^A modifications in the adaptive alterations of hypoxia and glycolysis of immune cells in TME.

#### T and B cells

T cells are an important component of the human immune system. T cell receptors are responsible for initiating signaling pathways that activate, inactivate or eliminate T cells and alterations in T cell-positive/negative regulatory factors can inhibit T cell function and mediate immune escape [127, 128]. Methylation of m^6^A affects T cells in multiple ways (including signaling pathways and glycolytic metabolism), leading to profound effects on T cell differentiation and function and generation of an immunosuppressive environment to facilitate tumor evasion of immune surveillance [70, 129]. FTO-mediated m^6^A demethylation in tumor cells enhances transcription factors, such as c-Jun, JunB and C/EBPβ, thereby inducing hypoxia-mediated glycolytic metabolism and suppressing CD8^+^ T cell function [129]. VHL deficiency regulates m^6^A methylation levels in ICOS through a HIF-1α-dependent glycolytic pathway and inhibits T cell maturation [70]. Interestingly, the METTL3/IGF2BP3 axis promotes tumor immune escape via m^6^A modification of PD-L1 mRNA and suppression of T cell activation in breast cancer [130]. These findings suggest that m^6^A methylation in tumor cells could remodel TME and mediate immune escape by affecting T cell functions. Moreover, m^6^A modifications in T cells appear to regulate the autoimmune response. Deletion of ALKBH5 in T cells leads to impairment of CD4^+^ T cell responses through reduction of mRNA stability and corresponding protein expression and decreased neutrophil recruitment into the central nervous system during neuroinflammation [131]. Furthermore, downregulation of METTL3 induces reduction of METTL3-mediated methylation of m^6^A targeting the IL-7/STAT5/SOCS pathway and stagnation of T cells at the naive stage [132]. Thus, in malignant TME, m^6^A methylation in both tumor and T cells impairs the immune response in vivo. However, the potential existence of a positive feedback loop between the two is yet to be established.

Reader proteins of m^6^A methylation play important regulatory roles in development of B cells [133]. For instance, NPM1 negatively regulates the growth and development of B and NK cells through glycolysis and YTHDF2-mediated methylation, possibly involving the mTORC1-mediated p53-hypoxia pathway [134]. Furthermore, downregulation of METTL14 may trigger severe defects in B cell development via inhibition of IL-7-induced pro-B cell proliferation and pre-B cell transition [122]. However, the detailed mechanisms by which m^6^A methylation achieves its effects on T and B cells, i.e., by inhibiting cell function and migration or suppressing proliferation and apoptosis (attributable to differences in immune cell types and m^6^A modification pathways and the effects of glycolysis) warrant comprehensive investigation.

#### Natural killer cells

Natural killer (NK) cells are irreplaceable components of the immune system due to their ability to directly identify and kill tumor cells [135]. m^6^A modifications regulate the anti-tumor function of NK cells in several ways [136]. As important regulators of NK cell antitumor immunity and homeostasis in vivo, METTL3- and YTHDF2-mediated m^6^A methylation can positively regulate antitumor immunity of NK cells [137]. YTHDF2 maintains NK cell maturation, homeostasis, and antitumor activity by regulating downstream target genes, including signal transduction and activator of transcription-5 (STAT-5), Eomesodermin and TARDBP [136]. Downregulation of METTL3 leads to NK cell hyporesponsiveness to IL-15 and promotes tumor immune escape via targeted effects on protein tyrosine phosphatase-2 [138]. An earlier bioinformatics study lacking experimental validation showed that NPM1 expression is negatively correlated with B and NK cells and regulated by YTHDF1-mediated m^6^A modifications as well as multiple glycolytic genes [134]. Considering the lack of detailed information, clarification of the mechanisms underlying m^6^A methylation-mediated effects of hypoxia and metabolic reprogramming on NK cell proliferation and activation is of significant interest for the purpose of therapeutic application.

#### Macrophages

Macrophages can polarize into activated macrophages with anti-tumor function (M1 type) or those that favor tumor proliferation (M2 type) [139]. Regulation of macrophage polarization by m^6^A methylation is manifested in several aspects and serves to maintain homeostasis of the microenvironment. For instance, upregulation of METTL3 significantly promotes M1 and inhibits M2 macrophage polarization [140]. METTL3 deficiency is associated with loss of m^6^A modifications on Irakm mRNA and slower degradation, ultimately resulting in inhibition of TLR -mediated macrophage activation [141]. FTO silencing suppresses the phosphorylation of IKKα/β, IκBα and p65 in the NF-κB signaling pathway and inhibits M1 and M2 macrophage polarization [142]. Glycolysis in TME has been shown to impair the anti-tumor function of macrophages. RNA-binding motif-4 (RBM4) interacts with YTHDF2 and degrades m^6^A-modified STAT1 mRNA, subsequently inhibiting IFN-γ-induced M1 macrophage polarization through regulation of glycolysis [143]. The collective findings provide key insights into the molecular mechanisms underlying m^6^A modification and metabolic reprogramming-mediated regulation of macrophages.

#### Granulocytes

The granulocyte family is classified into neutrophils, eosinophils and basophils, which perform phagocytic and bactericidal functions in the immune system. Granulocyte expression and function are known to be influenced by m^6^A modifications. Considerable evidence suggests that YTHDF2, METTL14, METTL3, and ZC3H13 are significantly correlated with the level of infiltration of neutrophils, macrophages, and eosinophils [144–146]. METTL3 and HNRNPC positively regulate CD4 memory-activated T cells and eosinophils in head-and-neck squamous cell carcinoma (HNSCC) [146]. Moreover, WTAP-dependent m^6^A methylation in neutrophils promotes glycolysis in breast cancer, supporting the potential involvement of hypoxia and metabolism in granulocyte regulation [147]. Promotion of granulocyte expression via m^6^A modification in tumor cells may be considered an adaptive immune response in the early tumor stages while tumor proliferation via m^6^A modification in granulocytes may be attributed to modulation of immune cells by malignant TME in the advanced tumor stages.

### m^6^A methylation promotes immunosuppressive cell functions

#### TAMs

Tumor-associated macrophages (TAMs) are at the core of the immunosuppressive cell and cytokine networks that play a critical role in tumor immune evasion through mechanisms associated with m^6^A modifications, such as promotion of TAM aggregation and immunosuppressive functions of ALKBH5 [148] and involvement of FTO in macrophage M2 polarization via the NF-κB pathway [32, 142]. TAM-induced regulation of immunosuppressive TME is a complex process and hypoxia-mediated m^6^A methylation may contribute to TAM recruitment and immunosuppression [32]. TAMs can inhibit antitumor immune cell functions and enhance the recruitment or differentiation of immunosuppressive cells. Hypoxia-mediated ALKBH5 is reported to significantly accelerate TAM recruitment and immunosuppression [134] and the m^6^A methyltransferase METTL14 in TAMs induces CD8^+^ T cell dysfunction and tumor progression [149]. In addition, METTL3 deficiency impairs YTHDF1-mediated SPRED2 translation, which enhances NF-κB and STAT3 activation via the ERK pathway, resulting in increased TAM-induced Treg infiltration into TME and tumor metastasis [150].

#### Treg

m^6^A methylation-dependent immune functions have also been reported in Treg, which serve as key immunosuppressive effector T cells in TME and are extensively regulated by m^6^A methylation-mediated SOCS genes. In mouse Tregs, METTL3/m^6^A methylation knockdown is reported to upregulate SOCS family genes through enhancing mRNA stability, thereby suppressing the IL-2/STAT5 pathway essential for Treg activity. Paradoxically, enhanced SOCS activity has been shown to inhibit IL-7-mediated STAT5 activation and T cell homeostatic proliferation and differentiation [132], whereas m^6^A methylation is critical for inducible degradation of SOCS mRNA in response to IL-7 signaling to reprogram naive T cells for proliferation and differentiation. This discrepancy may be ascribed to cell specificity and environmental effects on Treg m^6^A methylation-mediated regulatory effects. Furthermore, deletion of METTL3 results in an inability to activate natural T cell proliferation and METTL3-mediated inflammation in mice [151]. Our collective findings confirm the regulatory effects of m^6^A methylation on the immune function of Tregs, although it remains to be established whether (1) the malignant TME affects Treg function through m^6^A modifications and (2) other immunosuppressive cells can activate m^6^A methylation in Tregs and accelerate proliferation and differentiation via secretion of cytokines.

#### MDSCs

Myeloid-derived suppressor cells (MDSCs) are bone marrow-derived heterogeneous precursors of dendritic cells, macrophages and granulocytes [152] that exert immunosuppressive functions through multiple pathways [153] including negative regulation of immune responses in tumors and other diseases, in which m^6^A methylation plays an important role, for example, ALKBH5 promotes PD-L1 expression on monocytes/macrophages and reduces infiltration of MDSCs [154]. Accumulating evidence indicates that m^6^A modifications are involved in the differentiation and functional regulatory network of MDSCs to create an inhibitory microenvironment conducive to tumor growth, including high METTL3 expression in cervical cancer and m^6^A modification-mediated Olfr29-ps1/miR-214-3p/MyD88 regulation in renal cancer [155, 156]. ALKBH5 regulates the composition of tumor-infiltrating Tregs and MDSCs by affecting Mct4/Slc16a3 expression in TME, ultimately achieving enhanced immunosuppressive effects and promoting melanoma escape. Conversely, deletion of ALKBH5 attenuates the immunosuppressive function of MDSCs and enhances the efficacy of tumor immunotherapy [157]. Interestingly, elevated expression of METTL3 is associated with regulation of MDSC differentiation and poor prognosis of cervical cancer [155]. However, cisplatin-targeting METTL3 was used to block G-CSF methylation, which reduced the number of MDSCs, thereby achieving successful inhibition of bladder cancer progression [158]. Therefore, blocking specific m^6^A modifications in tumors may present a feasible technique for targeted tumor therapy. Notably, differentiation of MDSCs is a complex and variable process, metabolic programming products can also mediate immunosuppression of MDSCs through m^6^A methylation. For instance, the metabolite lactic acid upregulates METTL3 expression in MDSCs by inducing histone lactonization, leading to increased m^6^A modification and immunosuppressive activity, and ultimately, tumor immune escape [106]. However, limited reports to date have focused on establishing the effects of hypoxia and metabolic reprogramming on the status and behavior of MDSCs. Further research is essential to determine the interactions between the components of TME and their effects on the differentiation status of MDSCs.

#### MSCs and TA-MSCs

The interactions between mesenchymal stem cells (MSCs) and TME are complex. MSCs act as antigen-presenting cells (APCs) that activate tumor-adaptive immune responses to retard tumor growth [159]. In addition, MSCs have strong differentiation and proliferation capability and can inhibit multiple immune cells and promote tumor cell escape from immune surveillance by participating in generation of TME [160]. Studies to date have focused on the effect of m^6^A methylation on MSCs at the site of tumor metastasis rather than primary TME. m^6^A methylation is involved in the mechanism of differentiation of bone marrow-MSCs (BM-MSCs) into osteoblasts. METTL3 is upregulated in BM-MSCs during osteogenesis induction and its knockdown inhibits BM-MSC differentiation, which may be ascribed to a significant decrease in phosphorylation in the AKT signaling pathway [161]. Furthermore, BM-MSCs participate in TME remodeling via recruitment to tumor sites or may even transform directly into tumor cells through complex interactions with TME. BM-MSCs can differentiate into VECs, myofibroblasts and even CAFs, and secrete multiple factors in TME that strongly support tumor proliferation, angiogenesis, and invasion [162, 163]. Future studies should focus on the potential impact of m^6^A methylation on MSC behavior in TME. Importantly, MSCs can transform into TA-MSCs that strongly support tumor proliferation with the involvement of tumor-secreted cytokines. Although limited literature has documented the relationship between m^6^A and TA-MSCs, our group confirmed that TA-MSCs express high levels of macrophage migration inhibitory factor (MIF), which enhances FTO expression in pancreatic cancer cells and promotes proliferation, migration and invasion (unpublished results). Conversely, knockdown of MIF in TA-MSCs reduced FTO expression and attenuated the cancer-promoting effect. Similarly, knockdown of FTO in TA-MSCs decreased their pro-tumorigenic effect while overexpression of FTO rescued the decreased function of cancer cells induced by MIF knockdown in TA-MSCs. These findings clearly indicate that m^6^A modification affects the biological function of TA-MSCs, although the precise mechanisms linking m^6^A methylation with regulation of tumor progression remain to be established.

#### CAFs

Cancer-associated fibroblasts (CAFs) potently support tumor proliferation and metastasis, mainly through the activities of paracrine cytokines. Recent findings have revealed a critical role of Zfp217-FTO-YTHDF2 in fibroblast-mediated differentiation of adipocytes from 3T3L1 cells [164]. However, few studies have focused on the effects of m^6^A methylation in the secretory functions of CAFs, which should be extensively explored bearing in mind the multiple ways in which CAFs mediate tumor invasion. In addition, m^6^A modification is additionally implicated in EMT [165], tumor stem cell transformation [166], chemoresistance [167], and distant metastasis. The issue of whether hypoxia and metabolic reprogramming-mediated m^6^A methylation contribute to these processes via CAFs deserves further attention.

In TME, hypoxia and glycolysis-based metabolic reprogramming (metabolites such as lactic acid) are extensively involved in the functional regulation of immune cells and create an immunosuppressive microenvironment with the aid of m^6^A modifications that supports tumor growth. Immunosuppressive cells can inhibit the functions of anti-tumor immune cells. For example, METTL14 in TAM promotes dysfunction of CD8^+^ T cells and tumor progression [149]. In turn, tumor cells further support proliferation by activating m^6^A modifications in anti-tumor immune cells (such as Treg and TAM [150]) and converting them to immunosuppressive phenotypes through secretion of cytokines [136, 168]. Moreover, m^6^A modification in tumors regulates the levels of anti-tumor immune and immunosuppressive cells to shape the immunosuppressive TME. For instance, expression levels of METTL3 in breast cancer are negatively correlated with CD8^+^ T cells, helper T cells and activated NK cells and positively correlated with TAMs [169]. Given the complexity of TME, exploring the intricate crosstalk in the m^6^A methylation-regulated immune microenvironment could provide valuable insights for improving targeted treatment of tumors.

### Exosome and m^6^A methylation affect immune cell functions

Exosomes (30–150 nm in diameter) are extracellular vesicles (EVs) with a double lipid membrane [170]. Under pathological and physiological conditions, almost all cells (including tumor cells) secrete exosomes for metabolic distribution throughout the body [170]. As a significant medium for cellular communication, the intercellular signaling transmission function of exosomes is mainly attributed to the constituent proteins, nucleic acids and lipids [14] among which miRNA, mRNA, and lncRNA play irreplaceable roles [171]. The hypoxic environment accelerates tumor signaling, disrupts the balance of the normal TME through promoting exosome secretion by tumor cells and performs essential functions in a range of pathological conditions [172], including tumor invasion, immune escape, metabolic dysregulation, and chemoresistance [173], thus promoting accelerated tumor cell growth. However, tumor cell proliferation consumes oxygen, leading to more severe tissue hypoxia [174]. Thus, tumor exosome secretion promoted by hypoxia creates a vicious cycle in which they support each other to facilitate tumor proliferation and invasion. For instance, in hypoxic TME, CAFs alter their exosome content and increase exosome release through metabolic reprogramming [175], CAF-derived exosomes have been shown to inhibit the sensitivity of colorectal cancer cells to 5-fluorouracil and promote metastasis via the METTL3/miR-181d-5p axis [176].

Tu-Exo converts immune cells into a phenotype that favors tumor growth and promotes distant metastasis and chemoresistance of tumor cells by shaping the immunosuppressive TME [14, 177, 178]. The mechanism of this phenotypic shift in immune cells appears to be related to Tu-Exo-mediated cytokines and chemokines. Tu-Exo has been shown to inhibit T cell activity and induce apoptosis [179, 180]. Elevated Treg activity contributes to an immunosuppressive TME. Specifically, upregulation of chemokine ligand 20 in nasopharyngeal carcinoma-derived exosomes is reported to enhance recruitment of Treg to TME and increase its immunosuppressive function via Treg amplification [181]. Importantly, Tu-Exo carries cytokines and chemokines that transform MSCs into TA-MSCs with a pro-tumor phenotype. TA-MSCs have strong immune escape and intercellular signaling capabilities that generate a microenvironment favorable for tumor growth and metastasis. This TME remodeling drives the conversion of immune cells to a phenotype conducive to tumor proliferation [14]. TA-MSCs are able to regulate signaling through secreted exosomes, thereby affecting tumor proliferation and metastasis. For example, TA-MSCs promote M2 macrophage differentiation by inducing overexpression of PD-L1 in CD206^+^ macrophages, which accelerates tumor invasion [182]. A study by Yang et al. characterized the mechanisms by which Tu-Exo affects immune cell function and generates PMN. Specifically, Tu-Exo induces immunosuppression by downregulating NKG2D in NK cells and activates PI3K/AKT signaling to promote M2 macrophage polarization [183].

Immune escape, a key link in tumor proliferation, may be achieved by exosomal cargo-mediated m^6^A methylation. For instance, circNEIL3 contained in exosomes secreted by glioma cells accelerates tumor evasion of immune surveillance by blocking HECTD4-mediated ubiquitination to stabilize the m^6^A methylation regulator, IGF2BP3, and promote an immunosuppressive phenotype in macrophages [184]. Interestingly, tumor proliferation requires oxygen consumption and hypoxia promotes release of exosomes from tumor cells [174], Tu-Exo supports PMN formation by metabolic reprogramming or directly affecting immune cell function, facilitating tumor metastasis and invasion. Furthermore, exosome-carried substances can influence tumor progression through m^6^A methylation, which, in turn, produces ideal conditions for tumor growth and metastasis by regulating immune cell function [150, 185]. Accordingly, we suggest that as biomolecules that transmit signals over long distances, exosomes, not only influence conditions to support tumor metastasis, but also activate m^6^A methylation through multiple cargoes and create PMN by regulating immune cells to support tumor metastasis. Overall, we would encourage consideration of TME as a dynamically changing medium as a whole in which the components may be interrelated and act in concert to collectively influence the tumor development process.

## m^6^A methylation regulates the biological functions of tumor cells

**Fig. 2:**
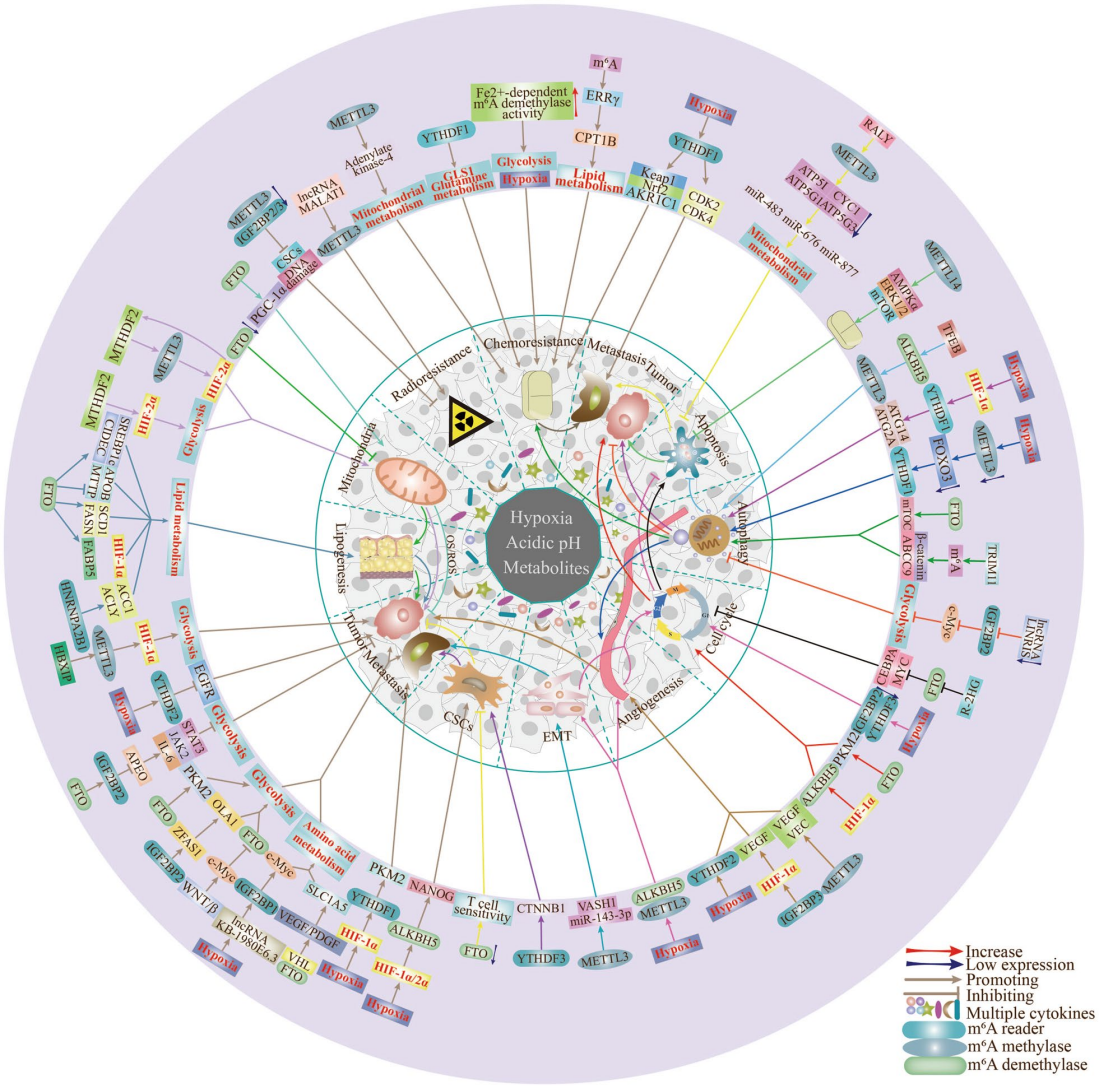
Effects of m^6^A modifications in hypoxia and metabolic TME on tumor biological functions. The different colored lines represent the interactions of m^6^A modifications in tumor biological functions through the respective pathways

With the substantial advances in oncology research, hypoxia, metabolism and m^6^A methylation have been shown to serve as regulatory factors in a variety of tumor-associated biological processes, including autophagy, apoptosis, chemotherapy resistance, and angiogenesis (Fig. 2, Table 2).

**Table 2:** Effects of m^6^A methylation on tumor biological functions

Types of tumor biological behavior	m^6^A regulators	Tumor types	Mechanism	Effects on biological behavior of tumors	References
Genomic instability	ALKBH5	Lung cancer	ALKBH5 improves the translation efficiency of FOXM1 mRNA by downregulating m^6^A methylation in FOXM1 mRNA	Promotes proliferation and invasion of lung adenocarcinoma cells under hypoxia	[316]
Genomic instability	METTL3	Lung cancer	METTL3-catalyzed m^6^A methylation mediates HMBOX1 gene downregulation, resulting in telomere dysfunction and inactivation of the p53 signaling pathway	Promotes cancer cell proliferation	[257]
Genomic instability	WTAP	HCC	WTAP downregulation suppresses the m^6^A methylation level, thereby blocking the G2/M phase transition	Mediates HCC occurrence	[317]
Cell cycle	ALKBH5	Renal cell carcinoma	Hypoxia-induced HIF-1α upregulates ALKBH5 expression	High ALKBH5 expression promotes tumor proliferation by increasing the percentage of cells in the G2/M phase	[250]
Cell cycle	METTL3	Uveal melanoma	Overexpression of METTL3 promotes tumor proliferation and colony formation by regulating the G1 phase	Promotes tumor proliferation	[309]
Cell cycle	IGF2BP1	HCC	The lncRNA HCG11 regulates IGF2BP1 to affect the G1 phase of HCC through MAPK signaling	Promotes tumorigenesis	[245]
Cell cycle	IGF2BP1	Renal cell carcinoma	IGF2BP1 promotes G1/S cell cycle transition by stabilizing mRNA	Enhances cell cycle progression and promotes tumor proliferation through m^6^A modifications	[318]
Cell cycle	IGF2BP1	Lung cancer	IGF2BP1 silencing induces cell cycle arrest and apoptosis through downregulation of Netrin-1	Inhibits tumor progression	[244]
Cell cycle	IGF2BP3	Renal cell carcinoma	DMDRMR binds IGF2BP3 to stabilize target genes and enhances cell cycle kinase CDK4 and G1-S phase transition	Promotes tumor progression	[247]
Autophagy	FTO	Kidney cancer	FTO-mediated demethylation prevents the decay of YTHDF2-dependent Unc-51-like kinase 1 mRNA by removing the methyl group from the 3′-UTR region	Promotes tumorigenesis	[226]
Autophagy	ALKBH5	Lung cancer	ALKBH5 improves the translation efficiency of lung cancer cells by decreasing the m^6^A methylation level of ubiquitin-binding enzyme E2C (UBE2C) mRNA	Autophagy is inhibited and accelerates migration and invasion of lung cancer cells	[319]
Autophagy/apoptosis	FTO	Ovarian cancer	FTO accelerates ovarian cancer cell proliferation by inhibiting apoptosis and activating autophagy	Promotes tumor proliferation	[320]
Autophagy	METTL3	Seminoma	Overexpression of METTL3 promotes autophagy and cisplatin resistance in tumors	Supports tumorigenesis and proliferation	[299]
Autophagy	YTHDF1	HCC	Hypoxia-mediated HIF-1α induces expression of YTHDF1, a m^6^A methylation reader, and promotes translation of autophagy-related genes ATG2A and ATG14 in a m^6^A methylation-dependent manner	Accelerates HCC autophagy and malignant tumorigenesis	[81]
Apoptosis	FTO	Colorectal cancer	microRNA-96 promotes anti-apoptosis in tumor cells by regulating the AMPKα2-FTO-m^6^A/MYC axis	Accelerates the growth and metastasis of colorectal cancer	[321]
Apoptosis	ALKBH5	Pancreatic cancer	ALKBH5 activates PER1 through transcription in a m^6^A-YTHDF2-dependent manner	Prevents cancer progression by promoting tumor cell apoptosis	[322]
Apoptosis	METTL14	Pancreatic cancer	Upregulation of METTL14 mediates cisplatin resistance by activating the AMPKα/ERK1/2/mTOR pathway to reduce autophagy and apoptosis	Promotes tumor progression	[233]
Apoptosis	METTL3	Colorectal cancer	METTL3 activates the glycolytic pathway and prevents tumor cell apoptosis by stabilizing the transcriptional translation of this gene	Promotes tumor proliferation	[57]
Angiogenesis	IGF2BP3	Colon cancer	IGF2BP3 binds to mRNA of cyclin D1 (cell cycle G1/S phase checkpoint) and regulates tumor angiogenesis by reading m^6^A methylation in the CDS region to reduce its mRNA stability	Promotes angiogenesis and metastasis in colon cancer	[192]
Angiogenesis	YTHDF3	Breast cancer	YTHDF3 induces the translation of m^6^A-enriched gene transcripts	Promotes breast cancer cell metastasis and invasion	[201]
Angiogenesis	YTHDF2	HCC	YTHDF2 processes the decay of m^6^A-containing interleukin 11 (IL11) and serpin family E member 2 (SERPINE2) mRNAs	Reduction of YTHDF2 contributes to angiogenesis and supports tumor metastasis	[195]
Angiogenesis	METTL3	Gastric cancer	P300-mediated activation of H3K27 acetylation in the METTL3 promoter induces METTL3 transcription, which stimulates m^6^A methylation of HDGF mRNA	Accelerates progression of gastric cancer	[197]
Drug resistance	METTL3	Lung cancer	Regulation of MALAT1-miR-1914-3p-YAP axis	Induces drug resistance and metastasis in lung cancer cells	[167]
Drug resistance	ALKBH5	Ovarian cancer	Overexpression of the ALKBH5-HOXA10 loop activates the JAK2/STAT3 signaling pathway	Induces cisplatin resistance in ovarian cancer	[323]
Drug resistance	METTL3	HCC	m^6^A methylation mediates autophagy in HCC via FOXO3	Promotes HCC sorafenib resistance	[202]
Drug resistance	YTHDF1	Colorectal cancer	YTHDF1 promotes cisplatin resistance by reprogramming GLS1-glutamine metabolism in colorectal cancer	Inhibits tumor cell death	[95]
EMT	METTL3	Leukemia	N6-methyladenosine regulates TGFβ1 expression and secretion to affect epithelial-mesenchymal transition of cancer cells	Promotes tumor progression	[324]
EMT	METTL3	Gastric cancer	METTL3 enhances the stability of ZMYM1 mRNA through m^6^A modification, which, in turn, promotes EMT by recruiting the CtBP/LSD1/CoREST complex to bind and mediate repression of the E-calmodulin promoter	Promotes gastric cancer metastasis	[325]
EMT	YTHDF1/METTL3	HCC	METTL3 and YTHDF1 mediate Snail mRNA translation to enhance EMT	Promotes tumor metastasis	[326]
EMT	METTL3	Lung cancer	The m^6^A methyltransferase METTL3 contributes to Transforming Growth Factor-beta-induced epithelial-mesenchymal transition of lung cancer cells through regulation of JUNB	Promotes tumor metastasis	[327]
EMT	METTL3	Ovarian cancer	METTL3 promotes EMT by regulating AXL translation	Promotes growth and invasion of ovarian cancer	[328]

### CSCs

Cancer stem cells (CSCs) are a leading cause of tumor recurrence and drug resistance. Hypoxia, a prominent feature of TME, is essential for rapid tumor proliferation and maintenance of stem cell function. Notably, specific m^6^A regulators act synergistically with HIF-1α and HIF-2α to promote the CSCs phenotype in multiple tumor types [166, 186]. The hypoxic microenvironment enriches stemness characteristics and CSCs levels through stimulating HIF and ALKBH5 expression in tumor cells [166]. In this biological pathway, HIF-1α/2α are considered upstream regulators of ALKBH5-mediated demethylation of the target gene NANOG, based on the finding that alterations in HIF-1α/2α expression affect ALKBH5 activity. Increased proportions of breast cancer stem cells can be achieved by dual regulation of HIF-1α/2α and ALKBH5 that induces higher expression and lower degradation levels of NANOG [166]. In addition to the hypoxia-HIF-1α/2α-ALKBH5-NANOG axis, an analogous mechanism involving hypoxia-HIF-ZNF17-m^6^A-NANOG/KLF4 signaling has been uncovered [187]. Similarly, in endometrial cancer, HIF-1α suppression downregulates ALKBH5 protein and inhibits the tumorigenicity of CSCs by reducing its demethylation capacity [186]. Moreover, PD-L1 signaling supports immune evasion and growth of CSCs, synergistically creating a hypoxic and immunosuppressive environment that facilitates tumor proliferation and invasion [188]. For example, FTO-mediated upregulation of PD-L1 in colon cancer cells promotes immune escape [189], while inhibition of FTO sensitizes tumor cells to T-cell toxicity and overcomes hypomethylating agent-induced immune evasion [190].

### m^6^A methylation promotes angiogenesis and tumor metastasis

Angiogenesis supplies nutrient and oxygen requirements and removes metabolic waste products [51], while invasion and metastasis are the leading causes of tumor-related death [191]. Neovascularization is an important process that provides adequate nutrients for metastatic invasion of tumors. A growing number of studies have evaluated the function of m^6^A methylation in tumor angiogenesis. For example, IGF2BP3 has been shown to bind vascular endothelial growth factor (VEGF) mRNA and upregulate its expression and stability through m^6^A methylation, thereby promoting angiogenesis in colon cancer [192]. Recent studies have focused on inducing vascular normalization in tumor cells and inhibiting malignant progression triggered by increased tumor hypoxia and dystrophy [193]. Hypoxia modulates the levels/activities of m^6^A regulators, in turn, altering m^6^A levels, thereby leading to increased target transcript expression and effects on tumor cell growth [194]. Regulation of the m^6^A reader YTHDF2 by hypoxia involves enhanced inflammation and angiogenesis and thus is critical for tumor invasion [195]. Overexpression of IGF2BP3 upregulates HIF-α in gastric cancer and promotes hypoxia-induced angiogenesis and tumor invasion [196]. In addition, tumor metabolism in hypoxic TME favors angiogenesis. Elevated expression of METTL3 in gastric cancer contributes to angiogenesis and liver metastasis by promoting HDGF secretion to support glycolysis [197]. Additionally, a role of m^6^A methylated mRNA in vascular endothelial cells (VECs) and vascular smooth muscle cells (VSMCs) of TME has been described. Knockdown of METTL3 significantly activates Notch signaling, which is associated with downregulation of heterodimeric Notch E3 ubiquitin ligase, and affects proliferation of VECs [198]. VEGF is considered the most potent and abundant specific vascular growth factor in angiogenesis that promotes metastasis [199]. METTL3-mediated m^6^A methylation positively regulates VEGF expression [200]. However, limited reports have comprehensively investigated the mechanisms involving m^6^A modifications by which hypoxia and metabolism affect vascular endothelial cells (VEC) and vascular smooth muscle cells (VSMC) and mediate tumor angiogenesis. Considering the clinical utility of anti-angiogenic therapy, targeting pro-angiogenic m^6^A methylation may provide an innovative avenue for treatment of solid tumors. Moreover, the specific mechanisms by which m^6^A methylation affects VEGF and other key genes in TME angiogenesis, such as Notch signaling, are worth further investigation.

The invasion and metastasis-promoting function of m^6^A methylation has been confirmed in breast cancer [201], liver cancer [202], lung cancer [165], gastric cancer [197]. However, tumor metastasis is a multistep process, designated the invasion-metastasis cascade [191, 203]. Therefore, the specific role of m^6^A methylation modifications at each step, from primary tumor cell development to metastasis formation, should be explored. For instance, the m^6^A reading protein, YTHDF3, potentiates tumorigenicity of cancer stem-like cells in ocular melanoma through facilitating translation of CTNNB1 [204]. The m^6^A methyltransferase METTL3 stimulates EMT in lung cancer through the miR-143-3p/VASH1 axis for metastasis and invasion [165]. Additionally, increased vascular permeability may be mediated by m^6^A methylation in the METTL3-YTHDF2-PKC-η/FAT4/PDGFRA signaling axis [205], which supports the possibility of transendothelial migration of tumor cells. Given the effects of hypoxia and metabolism on TME, a feasible consideration is that these factors promote metastasis in concert with m^6^A modifications. For instance, hypoxia in breast cancer induces HIF-1α expression and promotes the effect of PKM2 on glycolysis through upregulation of YTHDF1, triggering cancer cell growth and metastasis [206]. In fact, all known metastatic mechanisms involving CSCs [166], angiogenesis [196], EMT [207], chemoresistance [208] and autophagy [81] are associated with hypoxia and metabolism to varying degrees. However, the potential roles of m^6^A methylation in other metastatic processes, such as tumor cell entry and exit from dormancy, tumor-associated regulation of biological circadian rhythms, and signaling networks that promote metastatic colonization and evolution, remain to be established.

### m^6^A methylation is associated with therapeutic resistance

Similar to infectious diseases, tumors are often resistant to multiple treatment modalities and facilitate distant metastases [209], from traditional radiotherapy and chemotherapy to current targeted therapies and immunotherapy. Moreover, development of tumor resistance to combinations of drugs has been reported, which poses a considerable challenge. For improvement of therapeutic efficacy, the mechanisms underlying treatment resistance should be comprehensively understood. Interestingly, lung cancer cells adapt to the hypoxic microenvironment and mediate cisplatin resistance through the Keap1-Nrf2-AKR1C1 signaling axis by suppressing YTHDF1 expression, meanwhile, YTHDF1 inhibition regulates the translation of CDK2, CDK4 and cytokine D1, further promoting distant tumor metastasis [31]. Conversely, YTHDF1 gene expression is reported to be significantly upregulated in colorectal cancer, thereby reducing the sensitivity of tumor cells to cisplatin [210], suggesting that YTHDF1 mediates chemoresistance through distinct expression patterns in different cancer types. However, the mechanisms underlying TME crosstalk-induced treatment resistance are multifaceted. For instance, elevated endogenous Fe^2+^-dependent m^6^A demethylase activity promotes global m^6^A hypomethylation and post-transcriptional regulation of downstream genes involved in glycolysis, hypoxia, and immune checkpoint pathways, ultimately leading to treatment resistance in leukemia. This treatment-resistant phenotype could be effectively reversed by combination therapy with GNRa-CSP12 (a potential immunotherapeutic agent) and tyrosine kinase inhibitors [211]. Indeed, the involvement of m^6^A methylation in metabolic reprogramming after drug uptake and neutralization in TME may be a major factor in the development of therapeutic resistance. Another interesting finding is that YTHDF1 can promote cisplatin resistance by reprogramming GLS1-glutamine metabolism in colorectal cancer [95]. m^6^A-induced ERRγ stimulates fatty acid oxidation (FAO) and mediates chemoresistance via regulation of CPT1B, the rate-limiting enzyme of FAO [212]. Additionally, upregulation of METTL3 in tumors increases adenylate kinase-4 expression to promote mitochondrial metabolism-mediated tamoxifen resistance and tumor progression [213]. Similarly, a m^6^A-dependent mechanism enhances PDK1 mRNA stability and mediates temozolomide chemoresistance in glioblastoma by inducing an increase in glycolysis [214]. The Warburg effect (tumor cell glycolysis) promotes release of glioma exosomes carrying circ_0072083 that upregulates NANOG expression by targeting miR-1252-5p and mediating m^6^A demethylation, thereby promoting temozolomide resistance [215]. Therefore, comprehensive investigation of the mechanisms underlying m^6^A methylation-mediated chemoresistance may provide promising strategies for tumor treatment. Future studies should additionally focus on hypoxia, metabolism, and other TME components, including exosomes.

Radio-resistance is another cause of treatment failure. Various m^6^A methylation enzymes, such as METTL3, FTO, and ALKBH5, clearly affect key cellular processes in tumor cells, including apoptosis, resulting in resistance to radiation therapy [216–218]. Additionally, m^6^A methylation of the lncRNA MALAT1 is reported to induce radio-resistance/chemoresistance by impairing the apoptotic response in cervical cancer [217, 219]. Silencing of specific key m^6^A regulators, such as IGF2BP2/3 in lung cancer [218] and METTL3 in glioma [220], can reverse tumor radio-resistance via activation of DNA damage repair and inhibition of CSC functions. Elucidation of the molecular mechanisms underlying drug resistance in various malignancies is important to facilitate the development of individualized and precise therapeutic approaches.

### m^6^A methylation modulates autophagy

Autophagy is a type II programmed cell death mechanism [221]. The role of autophagy in tumors is currently controversial, but the general consensus is that when tumors progress to advanced stages and face a hostile environment, autophagy acts as a dynamic degradation and recycling system that contributes to the survival of established tumors and enhances aggressiveness by promoting metastasis [222]. Under strong environmental stimuli such as hypoxia, tumor cells maintain cellular integrity and support their survival and proliferation through autophagy [223]. Therefore, activation of autophagy represents an adaptive change of tumor cells in response to hypoxia that supports tumor survival. Mounting evidence supports the theory that the autophagy-associated mechanisms underlying tumor progression are associated with m^6^A methylation [81]. Mechanistically, hypoxia-mediated HIF-1α induces expression of the m^6^A methylation reader, YTHDF1, and promotes translation of autophagy-related genes, ATG2A and ATG14, in a m^6^A methylation-dependent manner, resulting in autophagy and tumorigenesis of HCC [81]. Angiogenesis and chemotherapy resistance are major contributory factors to the poor efficacy of antitumor therapy, and they can be achieved through autophagy. Low expression of the m^6^A methyltransferase, METTL3, under hypoxia is reported to trigger downregulation of FOXO3, thereby enhancing sorafenib resistance and angiogenic gene expression in HCC through YTHDF1-mediated autophagy, ultimately leading to poor prognosis [202]. Autophagy-mediated chemoresistance of gastric and nasopharyngeal carcinomas is similarly regulated by the mTORC signaling axis mediated by the m^6^A demethylase FTO and the β-catenin/ABCC9 signaling axis activated by m^6^A methylation on TRIM11 [224, 225]. In addition, multiple metabolic pathways (including lipid metabolism and glycolysis) are associated with autophagy-mediated tumor progression. FTO regulates obesity and adipogenesis through autophagy and provides energy for tumor survival through lipid metabolism. Mechanistically, FTO-mediated demethylation prevents YTHDF2-dependent decay of Unc-51-like kinase-1 (U51LK1) mRNA by removing methyl groups from the 3′-UTR region, thereby driving autophagy to promote tumorigenesis [226]. Hence, reduction of adipose accumulation through anti-autophagy pathways activated under conditions of FTO deficiency presents a critical strategy to prevent the harmful effects of increasing obesity [227]. Other m^6^A methylation regulators are additionally involved in modulation of autophagy. For example, knockdown of the lncRNA LINRIS in colorectal cancer blocks K139 ubiquitination of IGF2BP2 (a m^6^A reader), preventing its degradation by the autophagic lysosomal pathway and ultimately, attenuating the downstream pathways of IGF2BP2, such as MYC-mediated glycolysis in tumor cells [228]. These findings highlight critical interactions between autophagy and m^6^A methylation regulators, further confirming the biological significance of m^6^A methylation in hypoxia and metabolic programming in TME. However, the impact of m^6^A methylation regulators on the transcription and translation of autophagy-related genes through complex associations in TME and autophagy-related mechanisms involved in distant tumor metastasis (including angiogenesis, radio-resistance, tumor cell cycle, and EMT) remain poorly understood.

### m^6^A methylation is involved in apoptosis

Apoptosis is an orderly and coordinated cellular process that occurs under both physiological and pathological conditions. Deficiency of apoptosis induces persistent proliferation of tumor cells, which is one of the main reasons underlying poor prognosis [229, 230]. Clarification of the mechanisms associated with apoptosis may therefore provide useful information for targeted tumor therapy. Recent studies suggest that m^6^A methylation is closely associated with apoptosis [2]. As described by Chen et al. [231], m^6^A methylation regulates apoptosis through multiple mechanisms including modulation of apoptosis-related genes, silencing of genes encoding methylated or demethylated enzymes, and reduction of reading proteins-mediated transcripts. Hypoxia-mediated autophagy is involved in m^6^A modification-mediated regulation of apoptosis. Mechanistically, transcription factor EB (TREB, a major regulator of lysosomal biogenesis and autophagy genes) enhances the autophagic flux in hypoxic cardiomyocytes to inhibit apoptosis via induction of ALKBH5 and inhibition of METTL3 expression [232]. Furthermore, upregulation of METTL14 mediates cisplatin resistance through activating the AMPKα/ERK1/2/mTOR pathway to reduce autophagy and apoptosis, resulting in accelerated tumor proliferation and metastasis [233]. Tumor proliferation induced by apoptosis inhibition is, in fact, a multifactor-mediated biological behavior that incorporates EMT in addition to autophagy. Hypoxia favors tumor progression by interfering with the levels of m^6^A, leading to uncontrolled expression/activity of related genes. Specifically, the hypoxic environment promotes upregulation of METTL14/ALKBH5 [166, 234] and subsequently modulates m^6^A levels of EMT and angiogenesis-related transcripts (including genes involved in transforming growth factor-β signaling), leading to inappropriate cell cycle progression and tumor evasion of apoptosis [194]. Altered cellular metabolism is an adaptive adjustment mechanism by tumors in response to malignant stimuli such as hypoxia. METTL3 interacts directly with the 5′/3′UTR region of HK2 and the 3′UTR region of GLUT1 to activate the glycolytic pathway and prevents tumor apoptosis by stabilizing the transcriptional translation of these two genes [57]. In addition, a novel RNA-binding protein, RALY, systematically downregulates metabolism-related genes (ATP5I, ATP5G1, ATP5G3 and CYC1) through METTL3-mediated m^6^A methylation to promote post-transcriptional processing of specific miRNA subsets (miR-483, miR-676, and miR-877), thereby reprogramming mitochondrial metabolism in cancer cells to inhibit apoptosis and promote metastasis [235].

Tumor heterogeneity poses a considerable challenge in the delivery of effective tumor-targeted therapy. Targeting of m^6^A methylation regulators to induce apoptosis is proposed to provide a feasible therapeutic option [236]. For example, R-2-hydroxyglutaric acid (R-2HG) stimulates overall m^6^A methylation modifications of RNA in R-2HG-sensitive leukemia cells by inhibiting FTO activity, which, in turn, reduces the stability of MYC/CEBPA transcripts, thereby suppressing leukemia cell proliferation/viability and promoting cell cycle arrest (G2/M) and apoptosis [237]. Alterations in m^6^A RNA modifications can further modulate downstream adaptive responses, such as key regulators of apoptosis, autophagy, pro-survival and oncogenic pathways, CSCs and TME [238]. Thus, apoptotic evasion of tumor cells is the result of multiple factors and future precision tumor therapy needs to consider the associations among biological behaviors in malignant TME, including hypoxia and metabolism. m^6^A modifications are additionally reported to be involved in other cell death types, including necroptosis, ferroptosis, and pyroptosis, but their potential crosstalk with malignant TME has not been established [10]. Moreover, the relationship between more recently identified modes of cell death (cuproptosis and immunogenic cell death) and m^6^A methylation in tumor progression is worth further investigation [239, 240].

### m^6^A methylation regulates the cell cycle

Cell division is composed of G1 → S → G2 → M stages (designated the 'cell cycle') and its dysregulation is closely related to tumorigenesis. For example, dysregulation of the G1 phase suppresses DNA damage repair and promotes retinoblastoma progression [241]. m^6^A methylation has been shown to promote tumor proliferation through effects on the cell cycle [242]. An earlier study reported that IGF2BP3 increases the percentage of cells in the S phase and promotes proliferation of colorectal cancer cells through regulation of cyclin D1 expression [192]. Notably, the IGF2BP family modulates tumor progression in an m^6^A-dependent manner by affecting the cell cycle to varying degrees in multiple tumor types, including bladder cancer [243], lung cancer [244], HCC [245], endometrial cancer [246], and kidney cancer [247]. FTO-mediated m^6^A methylation of PKM2 promotes HCC progression by accelerating the generation of translation products and conversely, FTO knockdown induces G0/G1 phase blockage and inhibits tumor proliferation and growth in vivo [248]. Importantly, hypoxia-induced regulation of downstream biological behavior (e.g., the cell cycle) can be achieved through effects on reprogramming of the m^6^A-modified episodic transcriptome [249]. In renal cell carcinoma, hypoxia-induced HIF-1α enhances ALKBH5 expression, which, in turn, promotes tumor proliferation through increasing the percentage of cells in the G2/M phase [250]. Similarly, loss of YTHDF3 or IGF2BP2 in hypoxia induces a significant decrease in the percentage of cells in the G1 phase and significant increase in G2 phase cells, thereby promoting cell cycle progression and impeding apoptosis [251]. The collective findings suggest that hypoxia-mediated m^6^A modifications allow tumor cells to avoid apoptosis and continue to proliferate and induce distant metastasis through effects on the cell cycle. However, the issue of whether other biological behaviors (e.g., autophagy and glycolysis) are involved in regulation of the tumor cell cycle through m^6^A modifications is yet to be established.

### Genomic instability

Genomic instability (GI), which refers to the increased frequency of genomic alterations acquired by cells, is one of the most prevalent features of tumor cells and often associated with poor prognosis [252, 253]. Tumor cells with altered genomes (e.g., loss of apoptotic potential) are allowed to survive under hypoxic conditions that exacerbate tumor hypoxia, while persistent hypoxia increases genomic instability and heterogeneity and triggers environmental changes to support tumor cell proliferation [254]. RNA m^6^A methylation has recently been shown to regulate tumor progression through effects on GI [255]. Yin et al. [256] reported that multiple m^6^A regulators (METTL3, WTAP, YTHDF1/2/3) are associated with GI and mediate poor prognosis in HCC. Interestingly, METTL3-catalyzed m^6^A methylation is reported to mediate downregulation of the HMBOX1 gene, resulting in telomere dysfunction and inactivation of p53 signaling, thereby affecting telomere homeostasis and genome stability and promoting tumorigenesis [257]. Based on these findings, we propose that m^6^A methylation facilitates tumor growth through regulation of genomic stability. However, the issue of whether hypoxia or metabolism plays a role in this process and the underlying mechanisms are yet to be determined. Earlier studies support the involvement of m^6^A methylation in maintenance of genomic stability. For instance, METTL3-catalyzed m^6^A RNA methylation not only suppresses chromosomal translocation by driving RNA exosome recognition and 3′ end processing of SμGLT but also inhibits IgH-associated aberrant DNA breakage and prevents genomic instability [258]. The variable functions may be attributed to differences in tumor or cell type. However, m^6^A methylation in TME appears to primarily mediate the onset of GI. Theoretically, the maintenance of genomic stability is essential for efficient operation of the cell cycle. To our knowledge, few studies to date have focused on the mechanisms by which m^6^A modifications interfere with genomic stability and regulate tumor progression through the cell cycle.

### Extracellular matrix

Extracellular matrix (ECM) consists of basement membrane and intercellular stroma, which is an important tissue barrier for tumor metastasis. The tumor cells activate or secrete protein-degrading enzymes to degrade the matrix through adhesion of their surface receptors to various components in the ECM, thus forming a local lysis zone that constitutes a tumor cell metastasis runway. To some extent, m^6^A methylation is involved in the degradation of ECM and affects the progression of osteoarthritis [259], renal fibrosis [260], and tumors [261]. For instance, METTL3 involves the progression of osteoarthritis probably by affecting ECM degradation and regulating the inflammatory response [259]; the m^6^A methylation regulates endometrial carcinogenesis by targeting genes associated with ECM [261]. Indeed, m^6^A methylation is involved in cellular senescence and apoptosis through the regulation of ECM [262–264]. However, these studies have been focused more on non-tumor diseases, so there is a need to further investigate whether and how m^6^A methylation and ECM affect tumor development (e.g., senescence, apoptosis, and metabolism).

Importantly, hypoxia and metabolic programming jointly create a malignant immunosuppressive TME that supports multiple aspects of tumor development. Subsequently, tumor biological behaviors act synergistically to support disease progression in an m^6^A methylation-dependent manner. For example, hypoxia and glycolysis involved in m^6^A-dependent autophagy contribute to chemoresistance and inhibition of apoptosis and promote distant tumor metastasis. Persistent proliferation exacerbates hypoxia and mediates autophagy to promote further malignant growth. Tumor angiogenesis can also support chemoresistance and EMT formation as well as apoptosis inhibition. Indeed, some of the lesser reported m^6^A "readers" may also support tumor progression, high expression of YTHDC2 may increase mutation frequency in favor of tumorigenesis and portend a poor prognosis for patients with soft tissue sarcoma [265].

## Potential clinical applications of m^6^A methylation

Accumulating research has elucidated the mechanisms of m^6^A methylation in epigenetic regulation in tumorigenesis, providing valuable insights into the diagnostic and therapeutic applications (Table 3) of this modification.

**Table 3:** Tumor-targeted drug therapy based on m^6^A methylation

Drug name	Targeting	Tumor	Functions	References
Curcumin	ALKHB5	NA	Curcumin reduces ALKHB5 (m^6^A demethylase) expression in a m^6^A methylation-dependent manner to inhibit adipogenesis	[329]
Quercetin	METTL3	Cervical cancer	Quercetin acts synergistically with cisplatin to inhibit migration and invasion of cervical cancer cells by suppressing expression of METTL3	[330]
Baicalin	FTO/ALKBH5	Nasopharyngeal carcinoma	Baicalein affects genomic stability to inhibit tumor growth by mediating increase in METTL3 and METTL14 and decreases in FTO and ALKBH5	[331]
Saikosaponin	FTO	Myelomonocytic leukemia	Saikosaponin sensitizes myelomonocytic leukemia cells to tyrosine kinase inhibitors by suppressing FTO to rescue m^6^A hypermethylation in MYC and RARA	[332]
Simvastatin	METTL3	Lung cancer	Simvastatin mediates METTL3 downregulation and affects epithelial mesenchymal transition through m^6^A methylation of EZH2 mRNA, thereby inhibiting malignant progression of lung cancer	[333]
DAC51	FTO	Melanoma	Dac51 blocks FTO-mediated regulation of glycolytic metabolism and prevents tumor immune evasion by inhibiting FTO activity	[129]
Meclofenamic acid	FTO	Cervical cancer	Selective inhibition of FTO demethylation of ALKBH5 via m^6^A methylation	[276]
Alk-04	ALKBH5	Melanoma	Inhibition of ALKBH5 to regulate Mct4/Slc16a3 expression and lactate content in TME and the composition of tumor-infiltrating Treg and MDSCs	[157]
miR-4429	METTL3	Gastric cancer	miR-4429 prevents gastric cancer progression by targeting METTL3 to inhibit m^6^A methylation-induced stabilization of SEC62	[281]
miR-186	METTL3	HCC	miR-186 targets METTL3 and inhibits value-added invasion of HCC through the Wnt/β-catenin signaling pathway	[282]
Cisplatin	METTL3	Seminoma	Enhanced stability of TFAP2C mRNA may promote survival of cisplatin-loaded spermatocytoma cells through upregulation of DNA repair-related genes	[334]
Oxaliplatin	METTL3	Colorectal cancer	Depletion of Sec62 sensitizes tumor cells to chemotherapeutic agents by inhibiting Wnt/β-catenin signaling	[335]
Everolimus	METTL3	Gastric cancer	Inhibition of AKT/mTOR pathway to enhance drug sensitivity in tumor cells	[336]
Anti‑PD‑1	METTL3	Melanoma	Inhibition of NF-κB and STAT3 via suppressing the ERK pathway, thereby slowing tumor growth	[150]
STM2457	METTL3	Leukemia	STM2457 inhibits growth and differentiation and increases apoptosis of cancer cells by targeting METTL3	[337]
Imidazobenzoxazin-5-thione MV1035	ALKBH5	Glioblastoma	MV1035 reduces tumor migration and invasion by inhibiting ALKBH5	[338]
CS1/CS2	FTO	Leukemia	Inhibits expression of immune checkpoint genes (in particular, LILRB4) and significantly attenuates leukemic stem cell/initiating cell self-renewal and reprogramming immune responses through targeting FTO	[190]
Tyrosine kinase inhibitors	FTO	Leukemia	FTO-dependent m^6^A demethylation enhances mRNA stability of proliferation/survival transcripts containing m^6^A, leading to increased protein synthesis	[339]
Triptonide	IGF2BP1	Nasopharyngeal carcinoma	Inhibits tumor growth by disrupting the lncRNA THOR-IGF2BP1 signaling pathway	[340]
Berberine	IGF2BP3	Colorectal cancer	Blocks tumor proliferation by downregulating IGF2BP3, inducing a G0/G1 phase block	[341]
JQ1	IGF2BP3	Ewing sarcoma	Inhibits tumor growth by reducing IGF2BP3 expression	[342]
BTYNB	IGF2BP1	Melanoma	Suppresses IGF2BP1 protein expression and retards ovarian cancer growth by impairing interactions between IGF2BP1 and c-MYC or E2F1 mRNA	[273]
Benzamidobenzoic acid/ureidothiophene	IGF2BP2	Colorectal/HCC	Delays tumor progression through targeted inhibition of IGF2BP2	[343]
Chidamide	METTL3/WTAP	Lung cancer	Downregulates c-MET expression by suppressing its mRNA m^6^A methylation, leading to a subsequent c-MET-/HGF-dependent increase in crizotinib sensitivity of tumor cells	[344]

### Potential of m^6^A methylation in tumor diagnosis and prognosis

Effective biomarkers and assays with high sensitivity and specificity should greatly improve the efficiency of early tumor diagnosis. m^6^A methylation and its regulators are emerging biomarkers for tumor diagnosis and prognosis [266, 267]. Due to their metabolic reversibility, high abundance and stability, methylated nucleosides can be obtained in biological fluids or circulating cells [268, 269]. Huang et al. [268] showed elevated levels of m^6^A methylated RNA in circulating tumor cells from lung cancer patients using liquid chromatography-electrospray ionization tandem mass spectrometry (LC–ESI–MS/MS). Similarly, Pei and co-workers [270] reported elevated m^6^A methylation in peripheral blood leukocytes of lung cancer patients via flow cytometry, further supporting the potential of this modification as a biomarker. Multiple m^6^A methylation regulators have been shown to be associated with prognosis of different tumor types, including METTL3, WTAP, IGF2BP, FTO, and YTHDF [271]. Tumor prognosis is affected not only by gene expression but also the combined characteristics of multiple m^6^A methylation regulators. Although m^6^A methylation and its regulators show strong potential as biomarkers, their clinical application remains a considerable challenge due to the heterogeneity of m^6^A methylation in patients and lack of effective assays to detect site-specific m^6^A methylation from low-input clinical samples. In the future, single-cell sequencing technologies and spatial transcriptomic analyses may help to address these issues.

### Therapeutic potential of targeted m^6^A methylation modulators

Abnormal reduction or increase in specific m^6^A methylation regulators frequently cause dysregulation of overall levels of m^6^A methylation, thus affecting tumorigenesis, progression, and treatment. Currently, m^6^A methylation inhibitors are the most commonly used modality of targeted therapy, with reported positive antitumor effects. Indeed, inhibition of specific m^6^A regulators, such as METTL3 [104] and YTHDF1 [272], prevents glycolysis-mediated glucose uptake and lactate production, which offers promise for clinically targeted combination therapy. To date, a number of small-molecule inhibitors based on m^6^A modifications have been developed. For instance, BTYNB, a selective inhibitor of IGF2BP1, reduces IGF2BP1 protein expression and retards ovarian cancer growth by impairing interactions between IGF2BP1 and c-MYC or E2F1 mRNA [273]. Another specific inhibitor of ALKBH5, ALK-04, reduces Treg cell and MDSC infiltration and enhances the efficacy of anti-PD-1 therapy against tumor growth [157]. Targeted suppression of FTO with small-molecule inhibitors CS1 and CS2 can inhibit tumor proliferation through several pathways, including blockage of immune checkpoints (LILRB4) to suppress CSCs, induction of tumor cell cycle arrest (G0 phase), and suppression of immune escape through increasing tumor sensitivity to T cells, confirming the holistic nature of TME [190]. Given that silencing of a number of m^6^A regulators contributes to tumor proliferation and metastasis, upregulation of these molecules with the aid of specific agonists may present an effective option for m^6^A tumor-targeted therapy (e.g., certain ligands can act as activators of the METTL3-14-WTAP complex [274]). However, relatively few studies have been conducted in this area.

In addition to small-molecule inhibitors, a non-steroidal anti-inflammatory drug, maclofenamic acid (MA), specifically inhibits FTO demethylase activity and increases m^6^A methylation levels of mRNA. MA has been shown to effectively inhibit the growth and survival of glioblastoma stem cells and enhance the efficacy of the chemotherapeutic agent temozolomide [275, 276]. Other inhibitor compounds with similar antitumor effects have been identified. For example, 3-deazaadenosine inhibits METTL3/METTL14 [277], CA4 (carbonic anhydrase member) induces WTAP degradation and suppresses colorectal cancer proliferation [278], and R-2-hydroxyglutarate (R-2HG) delays leukemia progression via inhibition of FTO [237]. Moreover, METTL3-mediated autophagy-induced resistance to gefitinib could be reversed by β-elemene in lung cancer [279]. Although a variety of m^6^A modulator inhibitors hold promise for improving oncologic outcomes in clinical practice, limited relevant data are currently available, highlighting the urgent need for further clinical trials and development of effective strategies targeting further m^6^A methylation modulators.

Additionally, miRNAs are also feasible as inhibitors of m^6^A regulators, are non-coding RNA molecules with potent gene regulation properties shown to bind to target mRNAs, leading to inhibition of translation or degradation [280]. Notably, multiple miRNAs directly targeting m^6^A methyltransferases in tumors have been identified. In gastric cancer, miR-4429 acts as a tumor suppressor by targeting METTL3 to inhibit m^6^A methylation-induced stabilization of SEC62 and reduce tumor growth [281]. Interestingly, miR-186 and miR-600 appear to similarly target METTL3 and inhibit the progression of hepatoblastoma and lung cancer [282–284]. supporting the significant potential of miRNAs in m^6^A methylation-based targeted tumor therapy. However, the finding that specific miRNAs can target multiple genes complicates the development of selective miRNA-directed therapies. To identify efficacious therapeutic candidates, a clear picture of the miRNA targetome with information on the definitive number of oncogenes or tumor suppressors targeted by individual miRNAs is essential.

### Potential of m^6^A methylation in combination therapy

Targeted silencing or activation of m^6^A regulators can retard tumor proliferation. Radio- and chemoresistance are important factors in tumor recurrence. Therefore, combinations of m^6^A methylation inhibitors that block tumor growth and reverse radio- and chemoresistance may achieve better efficacy. In support of this theory, silencing of IGF2BP2/3, FTO, and METTL3 with specific inhibitors has been shown to suppress proliferation and reverse radio- and chemoresistance in multiple tumor types, including lung, cervical and pancreatic cancer, and glioma) [217, 218, 220, 275, 285], with ultimate improvement of treatment outcomes.

Tremendous progress in cancer immunotherapy has been made over the last decade. The most widely used immunotherapeutic agents at present are antibodies targeting immunosuppressive receptors such as CTLA-4, PD-1 and PD-L1 [286]. PD-1 is reported to downregulate the immune system response and promote immune tolerance by suppressing T-cell activity. Elucidation of the molecular mechanisms of various immunosuppressive TME is critical for the development of individualized and precise m^6^A methylation-mediated therapeutic approaches. Specific m^6^A modulators in anti-PD-1/L1 therapy have shown promise in eradication of malignant tumors [287]. Interestingly, in colorectal cancer and melanoma, enhanced anti-PD-1 efficacy could be achieved by depletion of METTL3 and METTL14, potentially via increased CD8^+^ T-cell infiltration and tumor killing function [288]. Moreover, knockdown of FTO led to increased PD-1 expression, thereby reversing melanoma resistance to anti-PD-1 therapy in preclinical trials [289]. Higher CD8^+^ T cell numbers reported in YTHDF1-deficient mice in TME are associated with prediction of improved PD-L1 checkpoint blockade outcomes [123]. These findings validate the ability of m^6^A modulators to modify the immune response against PD-1/L1 therapy. In fact, combining PD-L1 checkpoint blockade with YTHDF1 depletion enhances CD8^+^ T cell function and consequently slows tumor progression [123], providing a reference for the development of strategies utilizing m^6^A inhibitors in conjunction with PD-L1 therapy. Similarly, targeted inhibition of FTO and METTL3/14 combined with anti-PD-1 based immune checkpoint blockade therapy has been used for leukemia and colorectal cancer [190, 288]. The clinical value of integrated therapy based on immune checkpoint blockade with other m^6^A inhibitors is of considerable research interest. Combined inhibition of demethylase-FTO and immune checkpoint gene (LILRB4) has been shown to significantly attenuate leukemic stem cell/initiating cell self-renewal and reprogramming of immune responses [190].

Notably, hypoxia and metabolism are extensively involved in the complex crosstalk of TME through m^6^A methylation and promote tumor progression via multiple pathways. Therefore, multifaceted combination therapy should be considered. Accumulating evidence has revealed that HIF-1α and HIF-2α inhibitors block tumor growth through multiple mechanisms [290, 291]. For example, LAQ824 promotes polyubiquitination of HIF-1α through an unknown pathway, leading to inhibition of its function [292]. HIF-2α inhibitors, such as PT2385 and Vorinostat, suppress the HIF pathway by interfering with epigenetic mechanisms to achieve inhibition of soft tissue sarcoma and neuroblastoma, respectively [293, 294]. Combined inhibition of IGF2BP3 and HIF-1α is reported to further prevent tumor angiogenesis and metastasis [196]. Metabolic reprogramming in TME provides nutrition for tumor survival and proliferation. Therefore, targeting metabolic pathways associated with tumor proliferation and metastasis, such as glycolysis, mitochondrial metabolism, glutamate metabolism and autophagy, could provide an effective strategy for the development of novel drug discovery programs. For instance, an antitumor effect of BPTES, an inhibitor of glutaminase activity, has been reported [295]. Mitochondrial metabolism is critical for tumor growth. Metformin, an antidiabetic drug, has been shown to act as a key target pathway for cancer therapy through mitochondria-mediated metabolic pathways [296]. In this context, the drug biguanide phenformin, an inhibitor of mitochondrial complex I, displays anticancer activity [297]. Given the holistic nature of TME, combining these strategies with m^6^A methylation-targeted therapy may be a future research direction for the development of effective antitumor agents. For instance, METTL3 silencing can be synergistically implemented with the glycolysis inhibitor 2-deoxyglucose (2-DG) to block HCC growth [298] and combined inhibition of METTL3 and autophagy increases the sensitivity of spermatocytoma to cisplatin [299]. Additionally, co-inhibition of YTHDF2, ATF4-induced autophagy, and glutamine provides a novel strategy for targeted therapy in colorectal cancer [300].

## Conclusions and future perspectives

**Fig. 3:**
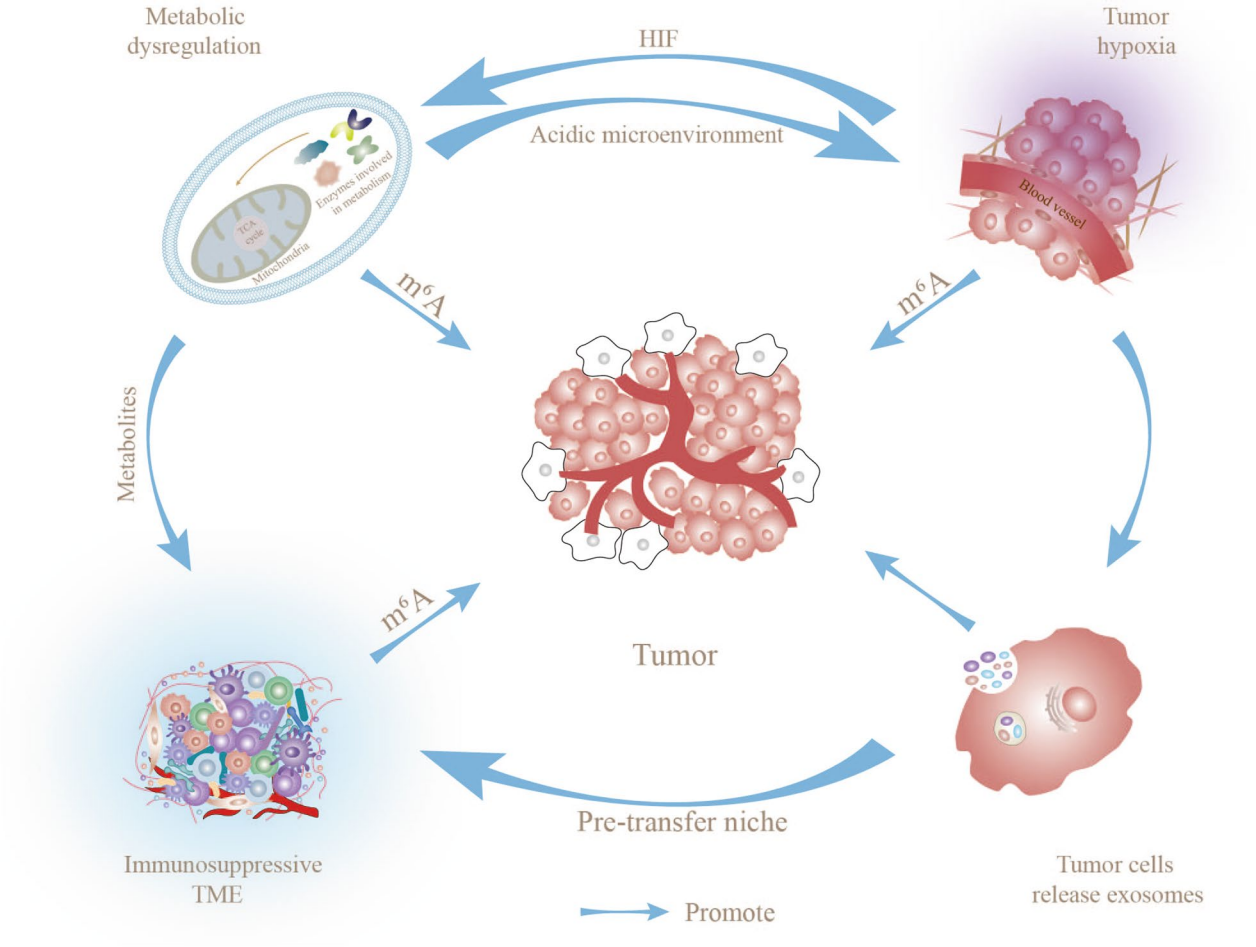
m^6^A methylation promotes immunosuppressive TME properties and supports tumor proliferation through pathways involving hypoxia, metabolic dysregulation, tumor exosomes and immune cells. HIF influences tumor cells under hypoxic conditions through m^6^A methylation modifications. Tumor cells in hypoxia accelerate the release of exosomes, contributing to the formation of immunosuppressive TME. m^6^A-mediated metabolic dysregulation generates an acidic environment that further supports tumor growth and exacerbates tumor hypoxia. A number of metabolites support immunosuppressive characteristics. In hypoxic conditions, tumors undergo metabolic reprogramming mediated by a HIF-induced positive feedback loop to further exacerbate metabolic dysregulation. Additionally, m^6^A methylation directly regulates immune cells to promote the progressive establishment of immunosuppressive TME

Hypoxia is commonly associated with tumor cell proliferation. To survive in such a hostile microenvironment, tumor cells utilize various strategies, such as regulating metabolic reprogramming and altering immune cell functions, thereby generating an immunosuppressive TME that supports their growth. Since the regulatory mechanisms of m^6^A methylation and immunosuppressive TME are not completely understood at present, we were unable to establish a perfect framework to understand the mutual crosstalk. However, a number of conclusions could be drawn based on the available data (Fig. 3): Tumor proliferation causes hypoxia in TME and hypoxia-mediated m^6^A methylation not only accelerates metabolite production by regulating metabolic reprogramming but also supports malignant progression by altering immune cell functions and phenotypes in the TME to promote immunosuppressive properties. In addition, these immunosuppressive signals are mediated by Tu-Exo for transfer to distant sites and generate an immunosuppressive TME by suppressing normal immune function to create conditions suitable for tumor cell metastasis and invasion. Hypoxia and metabolic reprogramming regulate multiple tumor biological behaviors in an m^6^A methylation-dependent manner. Tumor cells further support proliferation and metastasis through crosstalk among various mechanisms such as autophagy, inhibition of apoptosis, angiogenesis, and chemoresistance, generating a positive feedback cycle that results in poor prognosis. m^6^A methylation is extensively involved in the dynamic evolution of TME (hypoxia, metabolic dysregulation, functional transformation of immune cells, autophagy, angiogenesis, treatment resistance) and exacerbates the immunosuppressive properties of TME, providing potent conditions for adaptive changes and metastatic invasion of tumors. Furthermore, m^6^A methylation serves as an ideal marker for tumor surveillance, providing useful clinical information for tumor diagnosis and prediction that could be implemented in alteration of treatment regimens for optimal outcomes. Importantly, combination of strategies to remove and target m^6^A methylation, immunosuppressive TME-related approaches (e.g., targeting immune cells), and metabolic and hypoxia-based targeted therapies may provide novel and innovative avenues for clinical tumor therapy.

While targeted inhibitors based on m^6^A methylation regulators are relatively traditional therapeutic agents, their application is limited by low inhibition efficiency. Benefiting from the development of gene editing technology, m^6^A methylation-based therapies have been proposed to add or remove m^6^A methylation sites in specific genes. A novel tool, designated RCas9-FTO, facilitates sequence-specific demethylation of m^6^A in RNA via fusion of FTO with RCas9 [301]. This tool employs single guide RNA (sgRNA) to target RNA and PAMmer for enhancing their interactions, along with fusion with FTO to demethylate specific m^6^A sites. Another programmable in vivo manipulation tool, CRISPR-Cas13b-ALKBH5, has been utilized for targeted demethylation of specific mRNAs [302]. These tools do not require genomic alteration and are physiologically degradable but mRNA instability is a major challenge in the development of mRNA drugs. Regulation of structural elements involved in mRNA translation and metabolism via modification may present an effective strategy to address this problem.

Therapies based on suppression and targeting of TME immunosuppressive activity have gained significant attention in recent years. Currently, cancer immunotherapy is mainly based on immune checkpoint blockade technology, antibody/oncoprotein technology, CAR T cells, and small molecules. However, these approaches have a number of drawbacks, such as individualized differences and tumor heterogeneity, leading to non-response in some patients. Therefore, RNA-mediated immunotherapy may be an alternative option for tumor treatment [303]. Recently, Yeo and co-workers used CRISPR-Cas9 screening to identify 57 RNA-binding protein candidates with critical roles in promoting MYC-driven oncogenic pathways. Their study revealed an important function of YTHDF2 protein in global transcriptional regulation of MYC-driven breast cancer, highlighting the therapeutic applicability of RNA-binding proteins [304]. However, owing to complex tumor cell signaling mechanisms and immune cell phenotype shifts, no real breakthroughs have been made in this area. With the development of single-cell RNA sequencing and spatial transcriptome analysis, differences between tumor cell subpopulations should be identified, offering the possibility of addressing the temporal-spatial structure of tumor and immune cells (bearing in mind that spatial proximity between tumor and immune cells does not necessarily mean that actual communication is taking place). Furthermore, examination of how different cell subpopulations accomplish complex intercommunication and coordination in time and space may contribute to our understanding of the dynamics of TME.

Potential applications of exosomes as antitumor drug carriers are comprehensively discussed in a previous study by our group, which show utility as specific drug carriers for tumor-targeted therapy owing to low immunogenicity and biocompatibility [14, 305]. However, specific conditions of TME (such as hypoxia or metabolism) may affect the morphology or heterogeneity of exosomes and Tu-Exo may exert tumorigenic effects [306]. Therefore, studies on changes in exosome properties in TME (including how Tu-Exos are involved in metabolic reprogramming to influence tumor progression) and avoidance of Tu-Exo-mediated tumor proliferation are essential to facilitate their successful clinical application.

In summary, further in-depth exploration of the complex links and mechanisms between m^6^A methylation (including the implications of hypoxia and metabolic reprogramming-mediated crosstalk in immune cell function and tumor biological behavior) in TME should accelerate the clinical development of m^6^A methylation-based tumor immunotherapy. In view of the complex crosstalk within TME components, multifaceted combination therapies targeting m^6^A, hypoxia, metabolism, and the immune system should be considered.

## Data Availability

Not applicable.

## Abbreviations

2-DG: 2-Deoxyglucose
2-HG: 2-Hydroxyglutarate
APCs: Antigen-presenting cells
BM-MSCs: Bone marrow-MSCs
CAFs: Cancer-associated fibroblasts
CCR7: CC-chemokine receptor-7
CSCs: Cancer stem cells
DCs: Dendritic cells
ECM: Extracellular matrix
EMT: Epithelial-mesenchymal transition
EVs: Extracellular vesicles
FABP5: Fatty acid binding protein-5
FTO: Fat mass and obesity-associated protein
GI: Genomic instability
HBXIP: Hepatitis B virus X-interacting protein
HCC: Hepatocellular carcinoma
HIF: Hypoxia-inducible factors
HNSCC: Head and neck squamous cell carcinoma
IDH: Isocitrate dehydrogenase
MA: Maclofenamic acid
MDSCs: Myeloid-derived suppressor cells
MIF: Migration inhibitory factor
mRNA: Messenger RNA
MSCs: Mesenchymal stem cells
MTHFD2: Methylenetetrahydrofolate dehydrogenase-2
NK: Natural killer
OS: Oxidative stress
PKM2: Pyruvate-kinase-2
PMN: Pre-metastatic ecological niches
R-2HG: R-2-hydroxyglutaric acid
RBM4: RNA-binding motif-4
RBPs: RNA-binding proteins
sgRNA: Single guide RNA
TAMs: Tumor-associated macrophages
TA-MSCs: Tumor-associated mesenchymal stem cells
TCA: Tricarboxylic acid
TME: Tumor microenvironment
Treg: Regulatory T cell
Tu-Exo: Tumor-derived exosomes
U51LK1: Unc-51-like kinase-1
VECs: Vascular endothelial cells
VHL: Von Hippel–Lindau
VSMCs: Vascular smooth muscle cells
WTAP: Wilms tumor 1-associated protein

## References

[1] Wang T, Kong S, Tao M, Ju S (2020). The potential role of RNA N6-methyladenosine in cancer progression. Mol Cancer.

[2] Dominissini D, Moshitch-Moshkovitz S, Schwartz S, Salmon-Divon M, Ungar L, Osenberg S, Cesarkas K, Jacob-Hirsch J, Amariglio N, Kupiec M (2012). Topology of the human and mouse m6A RNA methylomes revealed by m6A-seq. Nature.

[3] Ping XL, Sun BF, Wang L, Xiao W, Yang X, Wang WJ, Adhikari S, Shi Y, Lv Y, Chen YS (2014). Mammalian WTAP is a regulatory subunit of the RNA N6-methyladenosine methyltransferase. Cell Res.

[4] Wang P, Doxtader KA, Nam Y (2016). Structural basis for cooperative function of Mettl3 and Mettl14 methyltransferases. Mol Cell.

[5] Xiao W, Adhikari S, Dahal U, Chen YS, Hao YJ, Sun BF, Sun HY, Li A, Ping XL, Lai WY (2016). Nuclear m(6)A reader YTHDC1 regulates mRNA splicing. Mol Cell.

[6] Wojtas MN, Pandey RR, Mendel M, Homolka D, Sachidanandam R, Pillai RS (2017). Regulation of m(6)A transcripts by the 3′→5′ RNA helicase YTHDC2 is essential for a successful meiotic program in the mammalian germline. Mol Cell.

[7] Liu N, Dai Q, Zheng G, He C, Parisien M, Pan T (2015). N(6)-methyladenosine-dependent RNA structural switches regulate RNA-protein interactions. Nature.

[8] Shi H, Wei J, He C (2019). Where, when, and how: context-dependent functions of RNA methylation writers, readers, and erasers. Mol Cell.

[9] Yen YP, Chen JA (2021). The m(6)A epitranscriptome on neural development and degeneration. J Biomed Sci.

[10] Liu L, Li H, Hu D, Wang Y, Shao W, Zhong J, Yang S, Liu J, Zhang J (2022). Insights into N6-methyladenosine and programmed cell death in cancer. Mol Cancer.

[11] Su R, Dong L, Li Y, Gao M, He PC, Liu W, Wei J, Zhao Z, Gao L, Han L (2022). METTL16 exerts an m(6)A-independent function to facilitate translation and tumorigenesis. Nat Cell Biol.

[12] Yang Z, Jiang X, Li D, Jiang X (2020). HBXIP promotes gastric cancer via METTL3-mediated MYC mRNA m6A modification. Aging (Albany NY).

[13] Yang Z, Jiang X, Zhang Z, Zhao Z, Xing W, Liu Y, Jiang X, Zhao H (2021). HDAC3-dependent transcriptional repression of FOXA2 regulates FTO/m6A/MYC signaling to contribute to the development of gastric cancer. Cancer Gene Ther.

[14] Zhang F, Guo J, Zhang Z, Qian Y, Wang G, Duan M, Zhao H, Yang Z, Jiang X (2021). Mesenchymal stem cell-derived exosome: a tumor regulator and carrier for targeted tumor therapy. Cancer Lett.

[15] Bejarano L, Jordāo MJC, Joyce JA (2021). Therapeutic targeting of the tumor microenvironment. Cancer Discov.

[16] Gu Y, Wu X, Zhang J, Fang Y, Pan Y, Shu Y, Ma P (2021). The evolving landscape of N(6)-methyladenosine modification in the tumor microenvironment. Mol Ther.

[17] Riera-Domingo C, Audigé A, Granja S, Cheng WC, Ho PC, Baltazar F, Stockmann C, Mazzone M (2020). Immunity, hypoxia, and metabolism—the Ménage à Trois of cancer: implications for immunotherapy. Physiol Rev.

[18] Wang Y, Wang Y, Ren Y, Zhang Q, Yi P, Cheng C (2022). Metabolic modulation of immune checkpoints and novel therapeutic strategies in cancer. Semin Cancer Biol.

[19] Liu Y, Wang M, Deng T, Liu R, Ning T, Bai M, Ying G, Zhang H, Ba Y (2022). Exosomal miR-155 from gastric cancer induces cancer-associated cachexia by suppressing adipogenesis and promoting brown adipose differentiation via C/EPBβ. Cancer Biol Med.

[20] Miyai Y, Sugiyama D, Hase T, Asai N, Taki T, Nishida K, Fukui T, Chen-Yoshikawa TF, Kobayashi H, Mii S (2022). Meflin-positive cancer-associated fibroblasts enhance tumor response to immune checkpoint blockade. Life Sci Alliance.

[21] Wang Y, Zhang Y, Du Y, Zhou M, Hu Y, Zhang S (2020). Emerging roles of N6-methyladenosine (m(6)A) modification in breast cancer. Cell Biosci.

[22] van den Homberg DAL, van der Kwast R, Quax PHA, Nossent AY (2022). N-6-methyladenosine in vasoactive microRNAs during hypoxia; a novel role for METTL4. Int J Mol Sci.

[23] An Y, Duan H (2022). The role of m6A RNA methylation in cancer metabolism. Mol Cancer.

[24] Guo L, Yang H, Zhou C, Shi Y, Huang L, Zhang J (2021). N6-methyladenosine RNA modification in the tumor immune microenvironment: novel implications for immunotherapy. Front Immunol.

[25] Daşu A, Toma-Daşu I, Karlsson M (2003). Theoretical simulation of tumour oxygenation and results from acute and chronic hypoxia. Phys Med Biol.

[26] Gilkes DM, Semenza GL, Wirtz D (2014). Hypoxia and the extracellular matrix: drivers of tumour metastasis. Nat Rev Cancer.

[27] Xu F, Huang M, Chen Q, Niu Y, Hu Y, Hu P, Chen D, He C, Huang K, Zeng Z (2021). LncRNA HIF1A-AS1 promotes gemcitabine resistance of pancreatic cancer by enhancing glycolysis through modulating the AKT/YB1/HIF1α pathway. Cancer Res.

[28] Wigerup C, Påhlman S, Bexell D (2016). Therapeutic targeting of hypoxia and hypoxia-inducible factors in cancer. Pharmacol Ther.

[29] Hu CJ, Wang LY, Chodosh LA, Keith B, Simon MC (2003). Differential roles of hypoxia-inducible factor 1alpha (HIF-1alpha) and HIF-2alpha in hypoxic gene regulation. Mol Cell Biol.

[30] Liu Y, Yan W, Tohme S, Chen M, Fu Y, Tian D, Lotze M, Tang D, Tsung A (2015). Hypoxia induced HMGB1 and mitochondrial DNA interactions mediate tumor growth in hepatocellular carcinoma through Toll-like receptor 9. J Hepatol.

[31] Shi Y, Fan S, Wu M, Zuo Z, Li X, Jiang L, Shen Q, Xu P, Zeng L, Zhou Y (2019). YTHDF1 links hypoxia adaptation and non-small cell lung cancer progression. Nat Commun.

[32] Dong F, Qin X, Wang B, Li Q, Hu J, Cheng X, Guo D, Cheng F, Fang C, Tan Y (2021). ALKBH5 facilitates hypoxia-induced paraspeckle assembly and IL8 secretion to generate an immunosuppressive tumor microenvironment. Cancer Res.

[33] Yang N, Wang T, Li Q, Han F, Wang Z, Zhu R, Zhou J (2021). HBXIP drives metabolic reprogramming in hepatocellular carcinoma cells via METTL3-mediated m6A modification of HIF-1α. J Cell Physiol.

[34] Zhong L, Liao D, Zhang M, Zeng C, Li X, Zhang R, Ma H, Kang T (2019). YTHDF2 suppresses cell proliferation and growth via destabilizing the EGFR mRNA in hepatocellular carcinoma. Cancer Lett.

[35] Ruan DY, Li T, Wang YN, Meng Q, Li Y, Yu K, Wang M, Lin JF, Luo LZ, Wang DS (2021). FTO downregulation mediated by hypoxia facilitates colorectal cancer metastasis. Oncogene.

[36] Damgaci S, Ibrahim-Hashim A, Enriquez-Navas PM, Pilon-Thomas S, Guvenis A, Gillies RJ (2018). Hypoxia and acidosis: immune suppressors and therapeutic targets. Immunology.

[37] Wang B, Zhao Q, Zhang Y, Liu Z, Zheng Z, Liu S, Meng L, Xin Y, Jiang X (2021). Targeting hypoxia in the tumor microenvironment: a potential strategy to improve cancer immunotherapy. J Exp Clin Cancer Res.

[38] Jellusova J, Cato MH, Apgar JR, Ramezani-Rad P, Leung CR, Chen C, Richardson AD, Conner EM, Benschop RJ, Woodgett JR, Rickert RC (2017). Gsk3 is a metabolic checkpoint regulator in B cells. Nat Immunol.

[39] Burrows N, Maxwell PH (2017). Hypoxia and B cells. Exp Cell Res.

[40] Francis A, Venkatesh GH, Zaarour RF, Zeinelabdin NA, Nawafleh HH, Prasad P, Buart S, Terry S, Chouaib S (2018). Tumor hypoxia: a key determinant of microenvironment hostility and a major checkpoint during the antitumor response. Crit Rev Immunol.

[41] Labiano S, Palazon A, Melero I (2015). Immune response regulation in the tumor microenvironment by hypoxia. Semin Oncol.

[42] Clambey ET, McNamee EN, Westrich JA, Glover LE, Campbell EL, Jedlicka P, de Zoeten EF, Cambier JC, Stenmark KR, Colgan SP, Eltzschig HK (2012). Hypoxia-inducible factor-1 alpha-dependent induction of FoxP3 drives regulatory T-cell abundance and function during inflammatory hypoxia of the mucosa. Proc Natl Acad Sci U S A.

[43] Ren L, Yu Y, Wang L, Zhu Z, Lu R, Yao Z (2016). Hypoxia-induced CCL28 promotes recruitment of regulatory T cells and tumor growth in liver cancer. Oncotarget.

[44] Chen X, Song E (2019). Turning foes to friends: targeting cancer-associated fibroblasts. Nat Rev Drug Discov.

[45] Ammirante M, Shalapour S, Kang Y, Jamieson CA, Karin M (2014). Tissue injury and hypoxia promote malignant progression of prostate cancer by inducing CXCL13 expression in tumor myofibroblasts. Proc Natl Acad Sci U S A.

[46] Haddad JJ, Harb HL (2005). Cytokines and the regulation of hypoxia-inducible factor (HIF)-1alpha. Int Immunopharmacol.

[47] Zhang J, Fan J, Zeng X, Nie M, Luan J, Wang Y, Ju D, Yin K (2021). Hedgehog signaling in gastrointestinal carcinogenesis and the gastrointestinal tumor microenvironment. Acta Pharm Sin B.

[48] Lin Y, Xu J, Lan H (2019). Tumor-associated macrophages in tumor metastasis: biological roles and clinical therapeutic applications. J Hematol Oncol.

[49] Wu JY, Huang TW, Hsieh YT, Wang YF, Yen CC, Lee GL, Yeh CC, Peng YJ, Kuo YY, Wen HT (2020). Cancer-derived succinate promotes macrophage polarization and cancer metastasis via succinate receptor. Mol Cell.

[50] Tan Y, Tang F, Li J, Yu H, Wu M, Wu Y, Zeng H, Hou K, Zhang Q (2022). Tumor-derived exosomes: the emerging orchestrators in melanoma. Biomed Pharmacother.

[51] Hanahan D, Weinberg RA (2011). Hallmarks of cancer: the next generation. Cell.

[52] Liberti MV, Locasale JW (2016). The Warburg effect: how does it benefit cancer cells?. Trends Biochem Sci.

[53] Denko NC (2008). Hypoxia, HIF1 and glucose metabolism in the solid tumour. Nat Rev Cancer.

[54] Martínez-Reyes I, Chandel NS (2021). Cancer metabolism: looking forward. Nat Rev Cancer.

[55] Hu Y, Tang J, Xu F, Chen J, Zeng Z, Han S, Wang F, Wang D, Huang M, Zhao Y (2022). A reciprocal feedback between N6-methyladenosine reader YTHDF3 and lncRNA DICER1-AS1 promotes glycolysis of pancreatic cancer through inhibiting maturation of miR-5586-5p. J Exp Clin Cancer Res.

[56] Yang D, Chang S, Li F, Ma M, Yang J, Lv X, Huangfu L, Jia C (2021). m(6) A transferase KIAA1429-stabilized LINC00958 accelerates gastric cancer aerobic glycolysis through targeting GLUT1. IUBMB Life.

[57] Shen C, Xuan B, Yan T, Ma Y, Xu P, Tian X, Zhang X, Cao Y, Ma D, Zhu X (2020). m(6)A-dependent glycolysis enhances colorectal cancer progression. Mol Cancer.

[58] Xue L, Li J, Lin Y, Liu D, Yang Q, Jian J, Peng J (2021). m(6) A transferase METTL3-induced lncRNA ABHD11-AS1 promotes the Warburg effect of non-small-cell lung cancer. J Cell Physiol.

[59] Wang Q, Guo X, Li L, Gao Z, Su X, Ji M, Liu J (2020). N(6)-methyladenosine METTL3 promotes cervical cancer tumorigenesis and Warburg effect through YTHDF1/HK2 modification. Cell Death Dis.

[60] Yu H, Zhao K, Zeng H, Li Z, Chen K, Zhang Z, Li E, Wu Z (2021). N(6)-methyladenosine (m(6)A) methyltransferase WTAP accelerates the Warburg effect of gastric cancer through regulating HK2 stability. Biomed Pharmacother.

[61] Fu J, Shinjo T, Li Q, St-Louis R, Park K, Yu MG, Yokomizo H, Simao F, Huang Q, Wu IH, King GL (2022). Regenerating glomerular metabolism and function by podocyte pyruvate kinase M2 in diabetic nephropathy. JCI Insight.

[62] Li J, Zhu L, Shi Y, Liu J, Lin L, Chen X (2019). m6A demethylase FTO promotes hepatocellular carcinoma tumorigenesis via mediating PKM2 demethylation. Am J Transl Res.

[63] Tateishi K, Iafrate AJ, Ho Q, Curry WT, Batchelor TT, Flaherty KT, Onozato ML, Lelic N, Sundaram S, Cahill DP (2016). Myc-driven glycolysis is a therapeutic target in glioblastoma. Clin Cancer Res.

[64] Yang X, Shao F, Guo D, Wang W, Wang J, Zhu R, Gao Y, He J, Lu Z (2021). WNT/β-catenin-suppressed FTO expression increases m(6)A of c-Myc mRNA to promote tumor cell glycolysis and tumorigenesis. Cell Death Dis.

[65] Lu S, Han L, Hu X, Sun T, Xu D, Li Y, Chen Q, Yao W, He M, Wang Z (2021). N6-methyladenosine reader IMP2 stabilizes the ZFAS1/OLA1 axis and activates the Warburg effect: implication in colorectal cancer. J Hematol Oncol.

[66] Huang J, Sun W, Wang Z, Lv C, Zhang T, Zhang D, Dong W, Shao L, He L, Ji X (2022). FTO suppresses glycolysis and growth of papillary thyroid cancer via decreasing stability of APOE mRNA in an N6-methyladenosine-dependent manner. J Exp Clin Cancer Res.

[67] Hou Y, Zhang Q, Pang W, Hou L, Liang Y, Han X, Luo X, Wang P, Zhang X, Li L, Meng X (2021). YTHDC1-mediated augmentation of miR-30d in repressing pancreatic tumorigenesis via attenuation of RUNX1-induced transcriptional activation of Warburg effect. Cell Death Differ.

[68] Uddin MB, Roy KR, Hosain SB, Khiste SK, Hill RA, Jois SD, Zhao Y, Tackett AJ, Liu YY (2019). An N(6)-methyladenosine at the transited codon 273 of p53 pre-mRNA promotes the expression of R273H mutant protein and drug resistance of cancer cells. Biochem Pharmacol.

[69] Dong S, Liang S, Cheng Z, Zhang X, Luo L, Li L, Zhang W, Li S, Xu Q, Zhong M (2022). ROS/PI3K/Akt and Wnt/β-catenin signalings activate HIF-1α-induced metabolic reprogramming to impart 5-fluorouracil resistance in colorectal cancer. J Exp Clin Cancer Res.

[70] Zhu Y, Zhao Y, Zou L, Zhang D, Aki D, Liu YC (2019). The E3 ligase VHL promotes follicular helper T cell differentiation via glycolytic-epigenetic control. J Exp Med.

[71] Mintz MA, Cyster JG (2020). T follicular helper cells in germinal center B cell selection and lymphomagenesis. Immunol Rev.

[72] Fong MY, Zhou W, Liu L, Alontaga AY, Chandra M, Ashby J, Chow A, O'Connor ST, Li S, Chin AR (2015). Breast-cancer-secreted miR-122 reprograms glucose metabolism in premetastatic niche to promote metastasis. Nat Cell Biol.

[73] Yan W, Wu X, Zhou W, Fong MY, Cao M, Liu J, Liu X, Chen CH, Fadare O, Pizzo DP (2018). Cancer-cell-secreted exosomal miR-105 promotes tumour growth through the MYC-dependent metabolic reprogramming of stromal cells. Nat Cell Biol.

[74] Zaidi N, Lupien L, Kuemmerle NB, Kinlaw WB, Swinnen JV, Smans K (2013). Lipogenesis and lipolysis: the pathways exploited by the cancer cells to acquire fatty acids. Prog Lipid Res.

[75] Svensson RU, Parker SJ, Eichner LJ, Kolar MJ, Wallace M, Brun SN, Lombardo PS, Van Nostrand JL, Hutchins A, Vera L (2016). Inhibition of acetyl-CoA carboxylase suppresses fatty acid synthesis and tumor growth of non-small-cell lung cancer in preclinical models. Nat Med.

[76] Zuo X, Chen Z, Gao W, Zhang Y, Wang J, Wang J, Cao M, Cai J, Wu J, Wang X (2020). M6A-mediated upregulation of LINC00958 increases lipogenesis and acts as a nanotherapeutic target in hepatocellular carcinoma. J Hematol Oncol.

[77] Fang R, Chen X, Zhang S, Shi H, Ye Y, Shi H, Zou Z, Li P, Guo Q, Ma L (2021). EGFR/SRC/ERK-stabilized YTHDF2 promotes cholesterol dysregulation and invasive growth of glioblastoma. Nat Commun.

[78] Zhao Z, Meng J, Su R, Zhang J, Chen J, Ma X, Xia Q (2020). Epitranscriptomics in liver disease: Basic concepts and therapeutic potential. J Hepatol.

[79] Chen A, Chen X, Cheng S, Shu L, Yan M, Yao L, Wang B, Huang S, Zhou L, Yang Z, Liu G (2018). FTO promotes SREBP1c maturation and enhances CIDEC transcription during lipid accumulation in HepG2 cells. Biochim Biophys Acta Mol Cell Biol Lipids.

[80] Guo H, Wang B, Xu K, Nie L, Fu Y, Wang Z, Wang Q, Wang S, Zou X (2020). m(6)A reader HNRNPA2B1 promotes esophageal cancer progression via up-regulation of ACLY and ACC1. Front Oncol.

[81] Li Q, Ni Y, Zhang L, Jiang R, Xu J, Yang H, Hu Y, Qiu J, Pu L, Tang J, Wang X (2021). HIF-1α-induced expression of m6A reader YTHDF1 drives hypoxia-induced autophagy and malignancy of hepatocellular carcinoma by promoting ATG2A and ATG14 translation. Signal Transduct Target Ther.

[82] Tang K, Zhu L, Chen J, Wang D, Zeng L, Chen C, Tang L, Zhou L, Wei K, Zhou Y (2021). Hypoxia promotes breast cancer cell growth by activating a glycogen metabolic program. Cancer Res.

[83] Ben-Haim MS, Pinto Y, Moshitch-Moshkovitz S, Hershkovitz V, Kol N, Diamant-Levi T, Beeri MS, Amariglio N, Cohen HY, Rechavi G (2021). Dynamic regulation of N(6),2′-O-dimethyladenosine (m(6)Am) in obesity. Nat Commun.

[84] Seo J, Jeong DW, Park JW, Lee KW, Fukuda J, Chun YS (2020). Fatty-acid-induced FABP5/HIF-1 reprograms lipid metabolism and enhances the proliferation of liver cancer cells. Commun Biol.

[85] Zhong X, Yu J, Frazier K, Weng X, Li Y, Cham CM, Dolan K, Zhu X, Hubert N, Tao Y (2018). Circadian clock regulation of hepatic lipid metabolism by modulation of m(6)A mRNA methylation. Cell Rep.

[86] Ramapriyan R, Caetano MS, Barsoumian HB, Mafra ACP, Zambalde EP, Menon H, Tsouko E, Welsh JW, Cortez MA (2019). Altered cancer metabolism in mechanisms of immunotherapy resistance. Pharmacol Ther.

[87] Li T, Le A (2018). Glutamine metabolism in cancer. Adv Exp Med Biol.

[88] Guo W, Zhang C, Feng P, Li M, Wang X, Xia Y, Chen D, Li J (2021). M6A methylation of DEGS2, a key ceramide-synthesizing enzyme, is involved in colorectal cancer progression through ceramide synthesis. Oncogene.

[89] Wang Y, Bai C, Ruan Y, Liu M, Chu Q, Qiu L, Yang C, Li B (2019). Coordinative metabolism of glutamine carbon and nitrogen in proliferating cancer cells under hypoxia. Nat Commun.

[90] Kaelin WG (2008). The von Hippel–Lindau tumour suppressor protein: O2 sensing and cancer. Nat Rev Cancer.

[91] Xiao Y, Thakkar KN, Zhao H, Broughton J, Li Y, Seoane JA, Diep AN, Metzner TJ, von Eyben R, Dill DL (2020). The m(6)A RNA demethylase FTO is a HIF-independent synthetic lethal partner with the VHL tumor suppressor. Proc Natl Acad Sci U S A.

[92] Zhang C, Zhang M, Ge S, Huang W, Lin X, Gao J, Gong J, Shen L (2019). Reduced m6A modification predicts malignant phenotypes and augmented Wnt/PI3K-Akt signaling in gastric cancer. Cancer Med.

[93] Liu HT, Zou YX, Zhu WJ, Sen L, Zhang GH, Ma RR, Guo XY, Gao P (2021). lncRNA THAP7-AS1, transcriptionally activated by SP1 and post-transcriptionally stabilized by METTL3-mediated m6A modification, exerts oncogenic properties by improving CUL4B entry into the nucleus. Cell Death Differ.

[94] Zhu P, He F, Hou Y, Tu G, Li Q, Jin T, Zeng H, Qin Y, Wan X, Qiao Y (2021). A novel hypoxic long noncoding RNA KB-1980E6.3 maintains breast cancer stem cell stemness via interacting with IGF2BP1 to facilitate c-Myc mRNA stability. Oncogene.

[95] Chen P, Liu XQ, Lin X, Gao LY, Zhang S, Huang X (2021). Targeting YTHDF1 effectively re-sensitizes cisplatin-resistant colon cancer cells by modulating GLS-mediated glutamine metabolism. Mol Ther Oncolytics.

[96] Courtney KD, Bezwada D, Mashimo T, Pichumani K, Vemireddy V, Funk AM, Wimberly J, McNeil SS, Kapur P, Lotan Y (2018). Isotope tracing of human clear cell renal cell carcinomas demonstrates suppressed glucose oxidation in vivo. Cell Metab.

[97] Green NH, Galvan DL, Badal SS, Chang BH, LeBleu VS, Long J, Jonasch E, Danesh FR (2019). MTHFD2 links RNA methylation to metabolic reprogramming in renal cell carcinoma. Oncogene.

[98] Zhuang C, Zhuang C, Luo X, Huang X, Yao L, Li J, Li Y, Xiong T, Ye J, Zhang F, Gui Y (2019). N6-methyladenosine demethylase FTO suppresses clear cell renal cell carcinoma through a novel FTO-PGC-1α signalling axis. J Cell Mol Med.

[99] Kang H, Zhang Z, Yu L, Li Y, Liang M, Zhou L (2018). FTO reduces mitochondria and promotes hepatic fat accumulation through RNA demethylation. J Cell Biochem.

[100] Mosca P, Robert A, Alberto JM, Meyer M, Kundu U, Hergalant S, Umoret R, Coelho D, Guéant JL, Leheup B, Dreumont N (2021). Vitamin B(12) deficiency dysregulates m6A mRNA methylation of genes involved in neurological functions. Mol Nutr Food Res.

[101] Yu XX, Liu YH, Liu XM, Wang PC, Liu S, Miao JK, Du ZQ, Yang CX (2018). Ascorbic acid induces global epigenetic reprogramming to promote meiotic maturation and developmental competence of porcine oocytes. Sci Rep.

[102] Cheng FY, Chiou YY, Hung SY, Lin TM, Wang HK, Lin CW, Liou HH, Chang MY, Wang HH, Lee YC (2021). Novel application of magnetite nanoparticle-mediated vitamin D3 delivery for peritoneal dialysis-related peritoneal damage. Int J Nanomedicine.

[103] Wang JN, Wang F, Ke J, Li Z, Xu CH, Yang Q, Chen X, He XY, He Y, Suo XG (2022). Inhibition of METTL3 attenuates renal injury and inflammation by alleviating TAB3 m6A modifications via IGF2BP2-dependent mechanisms. Sci Transl Med.

[104] Chen H, Gao S, Liu W, Wong CC, Wu J, Wu J, Liu D, Gou H, Kang W, Zhai J (2021). RNA N(6)-methyladenosine methyltransferase METTL3 facilitates colorectal cancer by activating the m(6)A-GLUT1-mTORC1 axis and is a therapeutic target. Gastroenterology.

[105] Fang C, He M, Li D, Xu Q (2021). YTHDF2 mediates LPS-induced osteoclastogenesis and inflammatory response via the NF-κB and MAPK signaling pathways. Cell Signal.

[106] Xiong J, He J, Zhu J, Pan J, Liao W, Ye H, Wang H, Song Y, Du Y, Cui B (2022). Lactylation-driven METTL3-mediated RNA m(6)A modification promotes immunosuppression of tumor-infiltrating myeloid cells. Mol Cell.

[107] Fischer K, Hoffmann P, Voelkl S, Meidenbauer N, Ammer J, Edinger M, Gottfried E, Schwarz S, Rothe G, Hoves S (2007). Inhibitory effect of tumor cell-derived lactic acid on human T cells. Blood.

[108] Kumagai S, Koyama S, Itahashi K, Tanegashima T, Lin YT, Togashi Y, Kamada T, Irie T, Okumura G, Kono H (2022). Lactic acid promotes PD-1 expression in regulatory T cells in highly glycolytic tumor microenvironments. Cancer Cell.

[109] Goetze K, Walenta S, Ksiazkiewicz M, Kunz-Schughart LA, Mueller-Klieser W (2011). Lactate enhances motility of tumor cells and inhibits monocyte migration and cytokine release. Int J Oncol.

[110] Brand A, Singer K, Koehl GE, Kolitzus M, Schoenhammer G, Thiel A, Matos C, Bruss C, Klobuch S, Peter K (2016). LDHA-associated lactic acid production blunts tumor immunosurveillance by T and NK cells. Cell Metab.

[111] Liu N, Luo J, Kuang D, Xu S, Duan Y, Xia Y, Wei Z, Xie X, Yin B, Chen F (2019). Lactate inhibits ATP6V0d2 expression in tumor-associated macrophages to promote HIF-2α-mediated tumor progression. J Clin Invest.

[112] Cohen AS, Geng L, Zhao P, Fu A, Schulte ML, Graves-Deal R, Washington MK, Berlin J, Coffey RJ, Manning HC (2020). Combined blockade of EGFR and glutamine metabolism in preclinical models of colorectal cancer. Transl Oncol.

[113] Püschel F, Favaro F, Redondo-Pedraza J, Lucendo E, Iurlaro R, Marchetti S, Majem B, Eldering E, Nadal E, Ricci JE (2020). Starvation and antimetabolic therapy promote cytokine release and recruitment of immune cells. Proc Natl Acad Sci U S A.

[114] Johnson MO, Wolf MM, Madden MZ, Andrejeva G, Sugiura A, Contreras DC, Maseda D, Liberti MV, Paz K, Kishton RJ (2018). Distinct regulation of Th17 and Th1 cell differentiation by glutaminase-dependent metabolism. Cell.

[115] Lagranha CJ, Senna SM, de Lima TM, Silva E, Doi SQ, Curi R, Pithon-Curi TC (2004). Beneficial effect of glutamine on exercise-induced apoptosis of rat neutrophils. Med Sci Sports Exerc.

[116] Fu Q, Xu L, Wang Y, Jiang Q, Liu Z, Zhang J, Zhou Q, Zeng H, Tong S, Wang T (2019). Tumor-associated macrophage-derived interleukin-23 interlinks kidney cancer glutamine addiction with immune evasion. Eur Urol.

[117] Mukha A, Kahya U, Dubrovska A (2021). Targeting glutamine metabolism and autophagy: the combination for prostate cancer radiosensitization. Autophagy.

[118] Currie E, Schulze A, Zechner R, Walther TC, Farese RV (2013). Cellular fatty acid metabolism and cancer. Cell Metab.

[119] Lucca LE, Hafler DA (2017). Resisting fatal attraction: a glioma oncometabolite prevents CD8+ T cell recruitment. J Clin Invest.

[120] Zhang B, Wu Q, Li B, Wang D, Wang L, Zhou YL (2020). m(6)A regulator-mediated methylation modification patterns and tumor microenvironment infiltration characterization in gastric cancer. Mol Cancer.

[121] Li B, Zhu L, Lu C, Wang C, Wang H, Jin H, Ma X, Cheng Z, Yu C, Wang S (2021). circNDUFB2 inhibits non-small cell lung cancer progression via destabilizing IGF2BPs and activating anti-tumor immunity. Nat Commun.

[122] Li M, Zha X, Wang S (2021). The role of N6-methyladenosine mRNA in the tumor microenvironment. Biochim Biophys Acta Rev Cancer.

[123] Han D, Liu J, Chen C, Dong L, Liu Y, Chang R, Huang X, Liu Y, Wang J, Dougherty U (2019). Anti-tumour immunity controlled through mRNA m(6)A methylation and YTHDF1 in dendritic cells. Nature.

[124] Bai X, Wong CC, Pan Y, Chen H, Liu W, Zhai J, Kang W, Shi Y, Yamamoto M, Tsukamoto T (2022). Loss of YTHDF1 in gastric tumors restores sensitivity to antitumor immunity by recruiting mature dendritic cells. J Immunother Cancer.

[125] Liu J, Zhang X, Chen K, Cheng Y, Liu S, Xia M, Chen Y, Zhu H, Li Z, Cao X (2019). CCR7 chemokine receptor-inducible lnc-Dpf3 restrains dendritic cell migration by inhibiting HIF-1α-mediated glycolysis. Immunity.

[126] Wang H, Hu X, Huang M, Liu J, Gu Y, Ma L, Zhou Q, Cao X (1898). Mettl3-mediated mRNA m(6)A methylation promotes dendritic cell activation. Nat Commun.

[127] Legut M, Gajic Z, Guarino M, Daniloski Z, Rahman JA, Xue X, Lu C, Lu L, Mimitou EP, Hao S (2022). A genome-scale screen for synthetic drivers of T cell proliferation. Nature.

[128] Braun DA, Wu CJ (2022). Tumor-infiltrating T cells—a portrait. N Engl J Med.

[129] Liu Y, Liang G, Xu H, Dong W, Dong Z, Qiu Z, Zhang Z, Li F, Huang Y, Li Y (2021). Tumors exploit FTO-mediated regulation of glycolytic metabolism to evade immune surveillance. Cell Metab.

[130] Wan W, Ao X, Chen Q, Yu Y, Ao L, Xing W, Guo W, Wu X, Pu C, Hu X (2022). METTL3/IGF2BP3 axis inhibits tumor immune surveillance by upregulating N(6)-methyladenosine modification of PD-L1 mRNA in breast cancer. Mol Cancer.

[131] Zhou J, Zhang X, Hu J, Qu R, Yu Z, Xu H, Chen H, Yan L, Ding C, Zou Q (2021). m(6)A demethylase ALKBH5 controls CD4(+) T cell pathogenicity and promotes autoimmunity. Sci Adv.

[132] Li HB, Tong J, Zhu S, Batista PJ, Duffy EE, Zhao J, Bailis W, Cao G, Kroehling L, Chen Y (2017). m(6)A mRNA methylation controls T cell homeostasis by targeting the IL-7/STAT5/SOCS pathways. Nature.

[133] Zheng Z, Zhang L, Cui XL, Yu X, Hsu PJ, Lyu R, Tan H, Mandal M, Zhang M, Sun HL (2020). Control of early B cell development by the RNA N(6)-methyladenosine methylation. Cell Rep.

[134] Liu XS, Zhou LM, Yuan LL, Gao Y, Kui XY, Liu XY, Pei ZJ (2021). NPM1 is a prognostic biomarker involved in immune infiltration of lung adenocarcinoma and associated with m6A modification and glycolysis. Front Immunol.

[135] Yilmaz A, Cui H, Caligiuri MA, Yu J (2020). Chimeric antigen receptor-engineered natural killer cells for cancer immunotherapy. J Hematol Oncol.

[136] Ma S, Yan J, Barr T, Zhang J, Chen Z, Wang LS, Sun JC, Chen J, Caligiuri MA, Yu J (2021). The RNA m6A reader YTHDF2 controls NK cell antitumor and antiviral immunity. J Exp Med.

[137] Li X, Ma S, Deng Y, Yi P, Yu J (2022). Targeting the RNA m(6)A modification for cancer immunotherapy. Mol Cancer.

[138] Song H, Song J, Cheng M, Zheng M, Wang T, Tian S, Flavell RA, Zhu S, Li HB, Ding C (2021). METTL3-mediated m(6)A RNA methylation promotes the anti-tumour immunity of natural killer cells. Nat Commun.

[139] Zhou J, Tang Z, Gao S, Li C, Feng Y, Zhou X (2020). Tumor-associated macrophages: recent insights and therapies. Front Oncol.

[140] Liu Y, Liu Z, Tang H, Shen Y, Gong Z, Xie N, Zhang X, Wang W, Kong W, Zhou Y, Fu Y (2019). The N(6)-methyladenosine (m(6)A)-forming enzyme METTL3 facilitates M1 macrophage polarization through the methylation of STAT1 mRNA. Am J Physiol Cell Physiol.

[141] Tong J, Wang X, Liu Y, Ren X, Wang A, Chen Z, Yao J, Mao K, Liu T, Meng FL (2021). Pooled CRISPR screening identifies m(6)A as a positive regulator of macrophage activation. Sci Adv.

[142] Gu X, Zhang Y, Li D, Cai H, Cai L, Xu Q (2020). N6-methyladenosine demethylase FTO promotes M1 and M2 macrophage activation. Cell Signal.

[143] Huangfu N, Zheng W, Xu Z, Wang S, Wang Y, Cheng J, Li Z, Cheng K, Zhang S, Chen X, Zhu J (2020). RBM4 regulates M1 macrophages polarization through targeting STAT1-mediated glycolysis. Int Immunopharmacol.

[144] Su G, Liu T, Han X, Sun H, Che W, Hu K, Xiao J, Li Y, Liu Y, Li W, Mei H (2021). YTHDF2 is a potential biomarker and associated with immune infiltration in kidney renal clear cell carcinoma. Front Pharmacol.

[145] Gong PJ, Shao YC, Yang Y, Song WJ, He X, Zeng YF, Huang SR, Wei L, Zhang JW (2020). Analysis of N6-methyladenosine methyltransferase reveals METTL14 and ZC3H13 as tumor suppressor genes in breast cancer. Front Oncol.

[146] Yi L, Wu G, Guo L, Zou X, Huang P (2020). Comprehensive analysis of the PD-L1 and immune infiltrates of m(6)A RNA methylation regulators in head and neck squamous cell carcinoma. Mol Ther Nucleic Acids.

[147] Ou B, Liu Y, Yang X, Xu X, Yan Y, Zhang J (2021). C5aR1-positive neutrophils promote breast cancer glycolysis through WTAP-dependent m6A methylation of ENO1. Cell Death Dis.

[148] Jiang Y, Wan Y, Gong M, Zhou S, Qiu J, Cheng W (2020). RNA demethylase ALKBH5 promotes ovarian carcinogenesis in a simulated tumour microenvironment through stimulating NF-κB pathway. J Cell Mol Med.

[149] Dong L, Chen C, Zhang Y, Guo P, Wang Z, Li J, Liu Y, Liu J, Chang R, Li Y (2021). The loss of RNA N(6)-adenosine methyltransferase Mettl14 in tumor-associated macrophages promotes CD8(+) T cell dysfunction and tumor growth. Cancer Cell.

[150] Yin H, Zhang X, Yang P, Zhang X, Peng Y, Li D, Yu Y, Wu Y, Wang Y, Zhang J (2021). RNA m6A methylation orchestrates cancer growth and metastasis via macrophage reprogramming. Nat Commun.

[151] Tong J, Cao G, Zhang T, Sefik E, Amezcua Vesely MC, Broughton JP, Zhu S, Li H, Li B, Chen L (2018). m(6)A mRNA methylation sustains Treg suppressive functions. Cell Res.

[152] Park MJ, Baek JA, Choi JW, Jang SG, Kim DS, Park SH, Cho ML, Kwok SK (2021). Programmed death-ligand 1 expression potentiates the immune modulatory function of myeloid-derived suppressor cells in systemic lupus erythematosus. Front Immunol.

[153] Saleh R, Toor SM, Taha RZ, Al-Ali D, Sasidharan Nair V, Elkord E (2020). DNA methylation in the promoters of PD-L1, MMP9, ARG1, galectin-9, TIM-3, VISTA and TGF-β genes in HLA-DR(-) myeloid cells, compared with HLA-DR(+) antigen-presenting cells. Epigenetics.

[154] Qiu X, Yang S, Wang S, Wu J, Zheng B, Wang K, Shen S, Jeong S, Li Z, Zhu Y (2021). M(6)A demethylase ALKBH5 regulates PD-L1 expression and tumor immunoenvironment in intrahepatic cholangiocarcinoma. Cancer Res.

[155] Ni HH, Zhang L, Huang H, Dai SQ, Li J (2020). Connecting METTL3 and intratumoural CD33(+) MDSCs in predicting clinical outcome in cervical cancer. J Transl Med.

[156] Shang W, Gao Y, Tang Z, Zhang Y, Yang R (2019). The pseudogene Olfr29-ps1 promotes the suppressive function and differentiation of monocytic MDSCs. Cancer Immunol Res.

[157] Li N, Kang Y, Wang L, Huff S, Tang R, Hui H, Agrawal K, Gonzalez GM, Wang Y, Patel SP, Rana TM (2020). ALKBH5 regulates anti-PD-1 therapy response by modulating lactate and suppressive immune cell accumulation in tumor microenvironment. Proc Natl Acad Sci U S A.

[158] Mu X, Wu K, Zhu Y, Zhu Y, Wang Y, Xiao L, Yao Z, Huang W, Sun F, Fan J (2021). Intra-arterial infusion chemotherapy utilizing cisplatin inhibits bladder cancer by decreasing the fibrocytic myeloid-derived suppressor cells in an m6A-dependent manner. Mol Immunol.

[159] Chan JL, Tang KC, Patel AP, Bonilla LM, Pierobon N, Ponzio NM, Rameshwar P (2006). Antigen-presenting property of mesenchymal stem cells occurs during a narrow window at low levels of interferon-gamma. Blood.

[160] Papait A, Stefani FR, Cargnoni A, Magatti M, Parolini O, Silini AR (2020). The multifaceted roles of MSCs in the tumor microenvironment: interactions with immune cells and exploitation for therapy. Front Cell Dev Biol.

[161] Wu Y, Xie L, Wang M, Xiong Q, Guo Y, Liang Y, Li J, Sheng R, Deng P, Wang Y (2018). Mettl3-mediated m(6)A RNA methylation regulates the fate of bone marrow mesenchymal stem cells and osteoporosis. Nat Commun.

[162] Chang X, Ma Z, Zhu G, Lu Y, Yang J (2021). New perspective into mesenchymal stem cells: Molecular mechanisms regulating osteosarcoma. J Bone Oncol.

[163] Tanno T, Lim Y, Wang Q, Chesi M, Bergsagel PL, Matthews G, Johnstone RW, Ghosh N, Borrello I, Huff CA, Matsui W (2014). Growth differentiating factor 15 enhances the tumor-initiating and self-renewal potential of multiple myeloma cells. Blood.

[164] Song T, Yang Y, Wei H, Xie X, Lu J, Zeng Q, Peng J, Zhou Y, Jiang S, Peng J (2019). Zfp217 mediates m6A mRNA methylation to orchestrate transcriptional and post-transcriptional regulation to promote adipogenic differentiation. Nucleic Acids Res.

[165] Wang H, Deng Q, Lv Z, Ling Y, Hou X, Chen Z, Dinglin X, Ma S, Li D, Wu Y (2019). N6-methyladenosine induced miR-143-3p promotes the brain metastasis of lung cancer via regulation of VASH1. Mol Cancer.

[166] Zhang C, Samanta D, Lu H, Bullen JW, Zhang H, Chen I, He X, Semenza GL (2016). Hypoxia induces the breast cancer stem cell phenotype by HIF-dependent and ALKBH5-mediated m^6^A-demethylation of NANOG mRNA. Proc Natl Acad Sci U S A.

[167] Jin D, Guo J, Wu Y, Du J, Yang L, Wang X, Di W, Hu B, An J, Kong L (2019). m(6)A mRNA methylation initiated by METTL3 directly promotes YAP translation and increases YAP activity by regulating the MALAT1-miR-1914-3p-YAP axis to induce NSCLC drug resistance and metastasis. J Hematol Oncol.

[168] Wang X, Ji Y, Feng P, Liu R, Li G, Zheng J, Xue Y, Wei Y, Ji C, Chen D, Li J (2021). The m6A reader IGF2BP2 regulates macrophage phenotypic activation and inflammatory diseases by stabilizing TSC1 and PPARγ. Adv Sci (Weinh).

[169] He X, Tan L, Ni J, Shen G (2021). Expression pattern of m(6)A regulators is significantly correlated with malignancy and antitumor immune response of breast cancer. Cancer Gene Ther.

[170] Yang D, Zhang W, Zhang H, Zhang F, Chen L, Ma L, Larcher LM, Chen S, Liu N, Zhao Q (2020). Progress, opportunity, and perspective on exosome isolation - efforts for efficient exosome-based theranostics. Theranostics.

[171] Valadi H, Ekström K, Bossios A, Sjöstrand M, Lee JJ, Lötvall JO (2007). Exosome-mediated transfer of mRNAs and microRNAs is a novel mechanism of genetic exchange between cells. Nat Cell Biol.

[172] Shao C, Yang F, Miao S, Liu W, Wang C, Shu Y, Shen H (2018). Role of hypoxia-induced exosomes in tumor biology. Mol Cancer.

[173] Meng W, Hao Y, He C, Li L, Zhu G (2019). Exosome-orchestrated hypoxic tumor microenvironment. Mol Cancer.

[174] Kumar A, Deep G (2020). Hypoxia in tumor microenvironment regulates exosome biogenesis: molecular mechanisms and translational opportunities. Cancer Lett.

[175] Ilkhani K, Bastami M, Delgir S, Safi A, Talebian S, Alivand MR (2021). The engaged role of tumor microenvironment in cancer metabolism: focusing on cancer-associated fibroblast and exosome mediators. Anticancer Agents Med Chem.

[176] Pan S, Deng Y, Fu J, Zhang Y, Zhang Z, Qin X (2022). N6-methyladenosine upregulates miR-181d-5p in exosomes derived from cancer-associated fibroblasts to inhibit 5-FU sensitivity by targeting NCALD in colorectal cancer. Int J Oncol.

[177] Mashouri L, Yousefi H, Aref AR, Ahadi AM, Molaei F, Alahari SK (2019). Exosomes: composition, biogenesis, and mechanisms in cancer metastasis and drug resistance. Mol Cancer.

[178] Wan M, Ning B, Spiegel S, Lyon CJ, Hu TY (2020). Tumor-derived exosomes (TDEs): how to avoid the sting in the tail. Med Res Rev.

[179] Taylor DD, Gerçel-Taylor C (2005). Tumour-derived exosomes and their role in cancer-associated T-cell signalling defects. Br J Cancer.

[180] Kim JW, Wieckowski E, Taylor DD, Reichert TE, Watkins S, Whiteside TL (2005). Fas ligand-positive membranous vesicles isolated from sera of patients with oral cancer induce apoptosis of activated T lymphocytes. Clin Cancer Res.

[181] Mrizak D, Martin N, Barjon C, Jimenez-Pailhes AS, Mustapha R, Niki T, Guigay J, Pancré V, de Launoit Y, Busson P (2015). Effect of nasopharyngeal carcinoma-derived exosomes on human regulatory T cells. J Natl Cancer Inst.

[182] Biswas S, Mandal G, Roy Chowdhury S, Purohit S, Payne KK, Anadon C, Gupta A, Swanson P, Yu X, Conejo-Garcia JR, Bhattacharyya A (2019). Exosomes produced by mesenchymal stem cells drive differentiation of myeloid cells into immunosuppressive M2-polarized macrophages in breast cancer. J Immunol.

[183] Yang X, Zhang Y, Zhang Y, Zhang S, Qiu L, Zhuang Z, Wei M, Deng X, Wang Z, Han J (2021). The key role of exosomes on the pre-metastatic Niche formation in tumors. Front Mol Biosci.

[184] Pan Z, Zhao R, Li B, Qi Y, Qiu W, Guo Q, Zhang S, Zhao S, Xu H, Li M (2022). EWSR1-induced circNEIL3 promotes glioma progression and exosome-mediated macrophage immunosuppressive polarization via stabilizing IGF2BP3. Mol Cancer.

[185] Wang Z, He J, Bach DH, Huang YH, Li Z, Liu H, Lin P, Yang J (2022). Induction of m(6)A methylation in adipocyte exosomal LncRNAs mediates myeloma drug resistance. J Exp Clin Cancer Res.

[186] Chen G, Liu B, Yin S, Li S, Guo Y, Wang M, Wang K, Wan X (2020). Hypoxia induces an endometrial cancer stem-like cell phenotype via HIF-dependent demethylation of SOX2 mRNA. Oncogenesis.

[187] Zhang C, Zhi WI, Lu H, Samanta D, Chen I, Gabrielson E, Semenza GL (2016). Hypoxia-inducible factors regulate pluripotency factor expression by ZNF217- and ALKBH5-mediated modulation of RNA methylation in breast cancer cells. Oncotarget.

[188] Plaks V, Kong N, Werb Z (2015). The cancer stem cell niche: how essential is the niche in regulating stemness of tumor cells?. Cell Stem Cell.

[189] Tsuruta N, Tsuchihashi K, Ohmura H, Yamaguchi K, Ito M, Ariyama H, Kusaba H, Akashi K, Baba E (2020). RNA N6-methyladenosine demethylase FTO regulates PD-L1 expression in colon cancer cells. Biochem Biophys Res Commun.

[190] Su R, Dong L, Li Y, Gao M, Han L, Wunderlich M, Deng X, Li H, Huang Y, Gao L (2020). Targeting FTO suppresses cancer stem cell maintenance and immune evasion. Cancer Cell.

[191] Lambert AW, Pattabiraman DR, Weinberg RA (2017). Emerging biological principles of metastasis. Cell.

[192] Yang Z, Wang T, Wu D, Min Z, Tan J, Yu B (2020). RNA N6-methyladenosine reader IGF2BP3 regulates cell cycle and angiogenesis in colon cancer. J Exp Clin Cancer Res.

[193] De Bock K, Cauwenberghs S, Carmeliet P (2011). Vessel abnormalization: another hallmark of cancer? Molecular mechanisms and therapeutic implications. Curr Opin Genet Dev.

[194] Panneerdoss S, Eedunuri VK, Yadav P, Timilsina S, Rajamanickam S, Viswanadhapalli S, Abdelfattah N, Onyeagucha BC, Cui X, Lai Z (2018). Cross-talk among writers, readers, and erasers of m(6)A regulates cancer growth and progression. Sci Adv.

[195] Hou J, Zhang H, Liu J, Zhao Z, Wang J, Lu Z, Hu B, Zhou J, Zhao Z, Feng M (2019). YTHDF2 reduction fuels inflammation and vascular abnormalization in hepatocellular carcinoma. Mol Cancer.

[196] Jiang L, Li Y, He Y, Wei D, Yan L, Wen H (2021). Knockdown of m6A reader IGF2BP3 inhibited hypoxia-induced cell migration and angiogenesis by regulating hypoxia inducible factor-1α in stomach cancer. Front Oncol.

[197] Wang Q, Chen C, Ding Q, Zhao Y, Wang Z, Chen J, Jiang Z, Zhang Y, Xu G, Zhang J (2020). METTL3-mediated m(6)A modification of HDGF mRNA promotes gastric cancer progression and has prognostic significance. Gut.

[198] Wang LJ, Xue Y, Huo R, Yan Z, Xu H, Li H, Wang J, Zhang Q, Cao Y, Zhao JZ (2020). N6-methyladenosine methyltransferase METTL3 affects the phenotype of cerebral arteriovenous malformation via modulating Notch signaling pathway. J Biomed Sci.

[199] Apte RS, Chen DS, Ferrara N (2019). VEGF in signaling and disease: beyond discovery and development. Cell.

[200] Chen X, Hua W, Huang X, Chen Y, Zhang J, Li G (2019). Regulatory role of RNA N(6)-methyladenosine modification in bone biology and osteoporosis. Front Endocrinol (Lausanne).

[201] Chang G, Shi L, Ye Y, Shi H, Zeng L, Tiwary S, Huse JT, Huo L, Ma L, Ma Y (2020). YTHDF3 induces the translation of m(6)A-enriched gene transcripts to promote breast cancer brain metastasis. Cancer Cell.

[202] Lin Z, Niu Y, Wan A, Chen D, Liang H, Chen X, Sun L, Zhan S, Chen L, Cheng C (2020). RNA m(6) A methylation regulates sorafenib resistance in liver cancer through FOXO3-mediated autophagy. Embo J.

[203] Gupta GP, Massagué J (2006). Cancer metastasis: building a framework. Cell.

[204] Xu Y, He X, Wang S, Sun B, Jia R, Chai P, Li F, Yang Y, Ge S, Jia R (2022). The m(6)A reading protein YTHDF3 potentiates tumorigenicity of cancer stem-like cells in ocular melanoma through facilitating CTNNB1 translation. Oncogene.

[205] Suo L, Liu C, Zhang QY, Yao MD, Ma Y, Yao J, Jiang Q, Yan B (2022). METTL3-mediated N (6)-methyladenosine modification governs pericyte dysfunction during diabetes-induced retinal vascular complication. Theranostics.

[206] Yao X, Li W, Li L, Li M, Zhao Y, Fang D, Zeng X, Luo Z (2022). YTHDF1 upregulation mediates hypoxia-dependent breast cancer growth and metastasis through regulating PKM2 to affect glycolysis. Cell Death Dis.

[207] Zhang C, Chen L, Liu Y, Huang J, Liu A, Xu Y, Shen Y, He H, Xu D (2021). Downregulated METTL14 accumulates BPTF that reinforces super-enhancers and distal lung metastasis via glycolytic reprogramming in renal cell carcinoma. Theranostics.

[208] Yu H, Yang X, Tang J, Si S, Zhou Z, Lu J, Han J, Yuan B, Wu Q, Lu Q, Yang H (2021). ALKBH5 inhibited cell proliferation and sensitized bladder cancer cells to cisplatin by m6A-CK2α-mediated glycolysis. Mol Ther Nucleic Acids.

[209] Acharyya S, Oskarsson T, Vanharanta S, Malladi S, Kim J, Morris PG, Manova-Todorova K, Leversha M, Hogg N, Seshan VE (2012). A CXCL1 paracrine network links cancer chemoresistance and metastasis. Cell.

[210] Wang S, Gao S, Zeng Y, Zhu L, Mo Y, Wong CC, Bao Y, Su P, Zhai J, Wang L (2021). N6-methyladenosine reader YTHDF1 promotes ARHGEF2 translation and RhoA signaling in colorectal cancer. Gastroenterology.

[211] Du Y, Han M, Cao K, Li Q, Pang J, Dou L, Liu S, Shi Z, Yan F, Feng S (2021). Gold nanorods exhibit intrinsic therapeutic activity via controlling N6-methyladenosine-based epitranscriptomics in acute myeloid leukemia. ACS Nano.

[212] Chen Z, Wu L, Zhou J, Lin X, Peng Y, Ge L, Chiang CM, Huang H, Wang H, He W (2020). N6-methyladenosine-induced ERRγ triggers chemoresistance of cancer cells through upregulation of ABCB1 and metabolic reprogramming. Theranostics.

[213] Liu X, Gonzalez G, Dai X, Miao W, Yuan J, Huang M, Bade D, Li L, Sun Y, Wang Y (2020). Adenylate kinase 4 modulates the resistance of breast cancer cells to tamoxifen through an m(6)A-based epitranscriptomic mechanism. Mol Ther.

[214] Li XD, Wang MJ, Zheng JL, Wu YH, Wang X, Jiang XB (2021). Long noncoding RNA just proximal to X-inactive specific transcript facilitates aerobic glycolysis and temozolomide chemoresistance by promoting stability of PDK1 mRNA in an m6A-dependent manner in glioblastoma multiforme cells. Cancer Sci.

[215] Ding C, Yi X, Chen X, Wu Z, You H, Chen X, Zhang G, Sun Y, Bu X, Wu X (2021). Warburg effect-promoted exosomal circ_0072083 releasing up-regulates NANGO expression through multiple pathways and enhances temozolomide resistance in glioma. J Exp Clin Cancer Res.

[216] Kowalski-Chauvel A, Lacore MG, Arnauduc F, Delmas C, Toulas C, Cohen-Jonathan-Moyal E, Seva C (2020). The m6A RNA demethylase ALKBH5 promotes radioresistance and invasion capability of glioma stem cells. Cancers (Basel).

[217] Taketo K, Konno M, Asai A, Koseki J, Toratani M, Satoh T, Doki Y, Mori M, Ishii H, Ogawa K (2018). The epitranscriptome m6A writer METTL3 promotes chemo- and radioresistance in pancreatic cancer cells. Int J Oncol.

[218] Zhou S, Bai ZL, Xia D, Zhao ZJ, Zhao R, Wang YY, Zhe H (2018). FTO regulates the chemo-radiotherapy resistance of cervical squamous cell carcinoma (CSCC) by targeting β-catenin through mRNA demethylation. Mol Carcinog.

[219] Peng L, Yuan X, Jiang B, Tang Z, Li GC (2016). LncRNAs: key players and novel insights into cervical cancer. Tumour Biol.

[220] Visvanathan A, Patil V, Arora A, Hegde AS, Arivazhagan A, Santosh V, Somasundaram K (2018). Essential role of METTL3-mediated m(6)A modification in glioma stem-like cells maintenance and radioresistance. Oncogene.

[221] Dikic I, Elazar Z (2018). Mechanism and medical implications of mammalian autophagy. Nat Rev Mol Cell Biol.

[222] Poillet-Perez L, White E (2019). Role of tumor and host autophagy in cancer metabolism. Genes Dev.

[223] Feng X, Zhang H, Meng L, Song H, Zhou Q, Qu C, Zhao P, Li Q, Zou C, Liu X, Zhang Z (2021). Hypoxia-induced acetylation of PAK1 enhances autophagy and promotes brain tumorigenesis via phosphorylating ATG5. Autophagy.

[224] Feng S, Qiu G, Yang L, Feng L, Fan X, Ren F, Huang K, Chen Y (2021). Biosci Rep.

[225] Zhang R, Li SW, Liu L, Yang J, Huang G, Sang Y (2020). TRIM11 facilitates chemoresistance in nasopharyngeal carcinoma by activating the β-catenin/ABCC9 axis via p62-selective autophagic degradation of Daple. Oncogenesis.

[226] Jin S, Zhang X, Miao Y, Liang P, Zhu K, She Y, Wu Y, Liu DA, Huang J, Ren J, Cui J (2018). m(6)A RNA modification controls autophagy through upregulating ULK1 protein abundance. Cell Res.

[227] Sarcognato S, Jong IEM, Fabris L, Cadamuro M, Guido M (2020). Necroptosis in cholangiocarcinoma. Cells.

[228] Wang Y, Lu JH, Wu QN, Jin Y, Wang DS, Chen YX, Liu J, Luo XJ, Meng Q, Pu HY (2019). LncRNA LINRIS stabilizes IGF2BP2 and promotes the aerobic glycolysis in colorectal cancer. Mol Cancer.

[229] Pistritto G, Trisciuoglio D, Ceci C, Garufi A, D'Orazi G (2016). Apoptosis as anticancer mechanism: function and dysfunction of its modulators and targeted therapeutic strategies. Aging (Albany NY).

[230] Wong RS (2011). Apoptosis in cancer: from pathogenesis to treatment. J Exp Clin Cancer Res.

[231] Chen J, Wang C, Fei W, Fang X, Hu X (2019). Epitranscriptomic m6A modification in the stem cell field and its effects on cell death and survival. Am J Cancer Res.

[232] Song H, Feng X, Zhang H, Luo Y, Huang J, Lin M, Jin J, Ding X, Wu S, Huang H (2019). METTL3 and ALKBH5 oppositely regulate m(6)A modification of TFEB mRNA, which dictates the fate of hypoxia/reoxygenation-treated cardiomyocytes. Autophagy.

[233] Kong F, Liu X, Zhou Y, Hou X, He J, Li Q, Miao X, Yang L (2020). Downregulation of METTL14 increases apoptosis and autophagy induced by cisplatin in pancreatic cancer cells. Int J Biochem Cell Biol.

[234] Zhou XL, Huang FJ, Li Y, Huang H, Wu QC (2021). SEDT2/METTL14-mediated m6A methylation awakening contributes to hypoxia-induced pulmonary arterial hypertension in mice. Aging (Albany NY).

[235] Sun L, Wan A, Zhou Z, Chen D, Liang H, Liu C, Yan S, Niu Y, Lin Z, Zhan S (2021). RNA-binding protein RALY reprogrammes mitochondrial metabolism via mediating miRNA processing in colorectal cancer. Gut.

[236] Niu Y, Lin Z, Wan A, Chen H, Liang H, Sun L, Wang Y, Li X, Xiong XF, Wei B (2019). RNA N6-methyladenosine demethylase FTO promotes breast tumor progression through inhibiting BNIP3. Mol Cancer.

[237] Su R, Dong L, Li C, Nachtergaele S, Wunderlich M, Qing Y, Deng X, Wang Y, Weng X, Hu C (2018). R-2HG exhibits anti-tumor activity by targeting FTO/m(6)A/MYC/CEBPA signaling. Cell.

[238] Li B, Jiang J, Assaraf YG, Xiao H, Chen ZS, Huang C (2020). Surmounting cancer drug resistance: new insights from the perspective of N(6)-methyladenosine RNA modification. Drug Resist Updat.

[239] Galluzzi L, Buqué A, Kepp O, Zitvogel L, Kroemer G (2017). Immunogenic cell death in cancer and infectious disease. Nat Rev Immunol.

[240] Tsvetkov P, Coy S, Petrova B, Dreishpoon M, Verma A, Abdusamad M, Rossen J, Joesch-Cohen L, Humeidi R, Spangler RD (2022). Copper induces cell death by targeting lipoylated TCA cycle proteins. Science.

[241] Massagué J (2004). G1 cell-cycle control and cancer. Nature.

[242] Li L, Chen YX, Yang B, Liao JY, Peng JW, Zhu S (2020). The crosstalk between RNA m(6)A epitranscriptome and TGFβ signaling pathway contributes to the arrest of cell cycle. Gene.

[243] Xie F, Huang C, Liu F, Zhang H, Xiao X, Sun J, Zhang X, Jiang G (2021). CircPTPRA blocks the recognition of RNA N(6)-methyladenosine through interacting with IGF2BP1 to suppress bladder cancer progression. Mol Cancer.

[244] Zhang J, Luo W, Chi X, Zhang L, Ren Q, Wang H, Zhang W (2020). IGF2BP1 silencing inhibits proliferation and induces apoptosis of high glucose-induced non-small cell lung cancer cells by regulating Netrin-1. Arch Biochem Biophys.

[245] Xu Y, Zheng Y, Liu H, Li T (2017). Modulation of IGF2BP1 by long non-coding RNA HCG11 suppresses apoptosis of hepatocellular carcinoma cells via MAPK signaling transduction. Int J Oncol.

[246] Zhang L, Wan Y, Zhang Z, Jiang Y, Gu Z, Ma X, Nie S, Yang J, Lang J, Cheng W, Zhu L (2021). IGF2BP1 overexpression stabilizes PEG10 mRNA in an m6A-dependent manner and promotes endometrial cancer progression. Theranostics.

[247] Gu Y, Niu S, Wang Y, Duan L, Pan Y, Tong Z, Zhang X, Yang Z, Peng B, Wang X (2021). DMDRMR-mediated regulation of m(6)A-modified CDK4 by m(6)A reader IGF2BP3 drives ccRCC progression. Cancer Res.

[248] Quinn JM, Greenwade MM, Palisoul ML, Opara G, Massad K, Guo L, Zhao P, Beck-Noia H, Hagemann IS, Hagemann AR (2019). Therapeutic inhibition of the receptor tyrosine kinase AXL improves sensitivity to platinum and taxane in ovarian cancer. Mol Cancer Ther.

[249] Wang YJ, Yang B, Lai Q, Shi JF, Peng JY, Zhang Y, Hu KS, Li YQ, Peng JW, Yang ZZ (2021). Reprogramming of m(6)A epitranscriptome is crucial for shaping of transcriptome and proteome in response to hypoxia. RNA Biol.

[250] Zhang X, Wang F, Wang Z, Yang X, Yu H, Si S, Lu J, Zhou Z, Lu Q, Wang Z, Yang H (2020). ALKBH5 promotes the proliferation of renal cell carcinoma by regulating AURKB expression in an m(6)A-dependent manner. Ann Transl Med.

[251] Xiao K, Liu P, Yan P, Liu Y, Song L, Liu Y, Xie L (2021). N6-methyladenosine reader YTH N6-methyladenosine RNA binding protein 3 or insulin like growth factor 2 mRNA binding protein 2 knockdown protects human bronchial epithelial cells from hypoxia/reoxygenation injury by inactivating p38 MAPK, AKT, ERK1/2, and NF-κB pathways. Bioengineered.

[252] Duijf PHG, Nanayakkara D, Nones K, Srihari S, Kalimutho M, Khanna KK (2019). Mechanisms of genomic instability in breast cancer. Trends Mol Med.

[253] Lord CJ, Ashworth A (2012). The DNA damage response and cancer therapy. Nature.

[254] Höckel M, Vaupel P (2001). Biological consequences of tumor hypoxia. Semin Oncol.

[255] Tian J, Ying P, Ke J, Zhu Y, Yang Y, Gong Y, Zou D, Peng X, Yang N, Wang X (2020). ANKLE1 N(6) -Methyladenosine-related variant is associated with colorectal cancer risk by maintaining the genomic stability. Int J Cancer.

[256] Yin T, Zhao L, Yao S (2022). Comprehensive characterization of m6A methylation and its impact on prognosis, genome instability, and tumor microenvironment in hepatocellular carcinoma. BMC Med Genomics.

[257] Lee JH, Hong J, Zhang Z, de la Peña AB, Proietti CJ, Deamicis AR, Guzmán GP, Lam HM, Garcia J, Roudier MP (2021). Regulation of telomere homeostasis and genomic stability in cancer by N (6)-adenosine methylation (m(6)A). Sci Adv.

[258] Nair L, Zhang W, Laffleur B, Jha MK, Lim J, Lee H, Wu L, Alvarez NS, Liu ZP, Munteanu EL (2021). Mechanism of noncoding RNA-associated N(6)-methyladenosine recognition by an RNA processing complex during IgH DNA recombination. Mol Cell.

[259] Sang W, Xue S, Jiang Y, Lu H, Zhu L, Wang C, Ma J (2021). METTL3 involves the progression of osteoarthritis probably by affecting ECM degradation and regulating the inflammatory response. Life Sci.

[260] Liu P, Zhang B, Chen Z, He Y, Du Y, Liu Y, Chen X (2020). m(6)A-induced lncRNA MALAT1 aggravates renal fibrogenesis in obstructive nephropathy through the miR-145/FAK pathway. Aging (Albany NY).

[261] Song K, Xu H, Wang C (2020). The role of N6-methyladenosine methylation in the progression of endometrial cancer. Cancer Biother Radiopharm.

[262] Ren J, Li Y, Wuermanbieke S, Hu S, Huang G (2022). N(6)-methyladenosine (m(6)A) methyltransferase METTL3-mediated LINC00680 accelerates osteoarthritis through m(6)A/SIRT1 manner. Cell Death Discov.

[263] Fang S, Zeng F, Chen R, Li M (2022). SIAH1 promotes senescence and apoptosis of nucleus pulposus cells to exacerbate disc degeneration through ubiquitinating XIAP. Tissue Cell.

[264] Shi L, Hu H, Sun P, Li Z, Ji L, Liu S, Zhang J (2022). RPL38 knockdown inhibits the inflammation and apoptosis in chondrocytes through regulating METTL3-mediated SOCS2 m6A modification in osteoarthritis. Inflamm Res.

[265] Hou M, Guo X, Chen Y, Cong L, Pan C (2020). A prognostic molecular signature of N^6^-methyladenosine methylation regulators for soft-tissue sarcoma from The Cancer Genome Atlas Database. Med Sci Monit.

[266] Huang H, Weng H, Chen J (2020). m(6)A modification in coding and non-coding RNAs: roles and therapeutic implications in cancer. Cancer Cell.

[267] Dong S, Wu Y, Liu Y, Weng H, Huang H (2021). N(6)-methyladenosine steers RNA metabolism and regulation in cancer. Cancer Commun (Lond).

[268] Huang W, Qi CB, Lv SW, Xie M, Feng YQ, Huang WH, Yuan BF (2016). Determination of DNA and RNA methylation in circulating tumor cells by mass spectrometry. Anal Chem.

[269] Gehrke CW, Kuo KC, Waalkes TP, Borek E (1979). Patterns of urinary excretion of modified nucleosides. Cancer Res.

[270] Pei Y, Lou X, Li K, Xu X, Guo Y, Xu D, Yang Z, Xu D, Cui W, Zhang D (2020). Peripheral blood leukocyte N6-methyladenosine is a noninvasive biomarker for non-small-cell lung carcinoma. Onco Targets Ther.

[271] He L, Li H, Wu A, Peng Y, Shu G, Yin G (2019). Functions of N6-methyladenosine and its role in cancer. Mol Cancer.

[272] Li Z, Peng Y, Li J, Chen Z, Chen F, Tu J, Lin S, Wang H (2020). N(6)-methyladenosine regulates glycolysis of cancer cells through PDK4. Nat Commun.

[273] Mahapatra L, Andruska N, Mao C, Le J, Shapiro DJ (2017). A novel IMP1 inhibitor, BTYNB, targets c-Myc and inhibits melanoma and ovarian cancer cell proliferation. Transl Oncol.

[274] Selberg S, Blokhina D, Aatonen M, Koivisto P, Siltanen A, Mervaala E, Kankuri E, Karelson M (2019). Discovery of small molecules that activate RNA methylation through cooperative binding to the METTL3-14-WTAP complex active site. Cell Rep.

[275] Xiao L, Li X, Mu Z, Zhou J, Zhou P, Xie C, Jiang S (2020). FTO inhibition enhances the antitumor effect of temozolomide by targeting MYC-miR-155/23a cluster-MXI1 feedback circuit in glioma. Cancer Res.

[276] Huang Y, Yan J, Li Q, Li J, Gong S, Zhou H, Gan J, Jiang H, Jia GF, Luo C, Yang CG (2015). Meclofenamic acid selectively inhibits FTO demethylation of m6A over ALKBH5. Nucleic Acids Res.

[277] Fustin JM, Doi M, Yamaguchi Y, Hida H, Nishimura S, Yoshida M, Isagawa T, Morioka MS, Kakeya H, Manabe I, Okamura H (2013). RNA-methylation-dependent RNA processing controls the speed of the circadian clock. Cell.

[278] Zhang J, Tsoi H, Li X, Wang H, Gao J, Wang K, Go MY, Ng SC, Chan FK, Sung JJ, Yu J (2016). Carbonic anhydrase IV inhibits colon cancer development by inhibiting the Wnt signalling pathway through targeting the WTAP-WT1-TBL1 axis. Gut.

[279] Liu S, Li Q, Li G, Zhang Q, Zhuo L, Han X, Zhang M, Chen X, Pan T, Yan L (2020). The mechanism of m(6)A methyltransferase METTL3-mediated autophagy in reversing gefitinib resistance in NSCLC cells by β-elemene. Cell Death Dis.

[280] Sun YM, Chen YQ (2020). Principles and innovative technologies for decrypting noncoding RNAs: from discovery and functional prediction to clinical application. J Hematol Oncol.

[281] He H, Wu W, Sun Z, Chai L (2019). MiR-4429 prevented gastric cancer progression through targeting METTL3 to inhibit m(6)A-caused stabilization of SEC62. Biochem Biophys Res Commun.

[282] Cui X, Wang Z, Li J, Zhu J, Ren Z, Zhang D, Zhao W, Fan Y, Zhang D, Sun R (2020). Cross talk between RNA N6-methyladenosine methyltransferase-like 3 and miR-186 regulates hepatoblastoma progression through Wnt/β-catenin signalling pathway. Cell Prolif.

[283] Wei W, Huo B, Shi X (2019). miR-600 inhibits lung cancer via downregulating the expression of METTL3. Cancer Manag Res.

[284] Wang WT, Han C, Sun YM, Chen TQ, Chen YQ (2019). Noncoding RNAs in cancer therapy resistance and targeted drug development. J Hematol Oncol.

[285] Hao CC, Xu CY, Zhao XY, Luo JN, Wang G, Zhao LH, Ge X, Ge XF (2020). Up-regulation of VANGL1 by IGF2BPs and miR-29b-3p attenuates the detrimental effect of irradiation on lung adenocarcinoma. J Exp Clin Cancer Res.

[286] Bagchi S, Yuan R, Engleman EG (2021). Immune checkpoint inhibitors for the treatment of cancer: clinical impact and mechanisms of response and resistance. Annu Rev Pathol.

[287] Chen L, Han X (2015). Anti-PD-1/PD-L1 therapy of human cancer: past, present, and future. J Clin Invest.

[288] Wang L, Hui H, Agrawal K, Kang Y, Li N, Tang R, Yuan J, Rana TM (2020). m(6) A RNA methyltransferases METTL3/14 regulate immune responses to anti-PD-1 therapy. Embo J.

[289] Yang S, Wei J, Cui YH, Park G, Shah P, Deng Y, Aplin AE, Lu Z, Hwang S, He C, He YY (2019). m(6)A mRNA demethylase FTO regulates melanoma tumorigenicity and response to anti-PD-1 blockade. Nat Commun.

[290] Murugesan T, Rajajeyabalachandran G, Kumar S, Nagaraju S, Jegatheesan SK (2018). Targeting HIF-2α as therapy for advanced cancers. Drug Discov Today.

[291] Semenza GL (2012). Hypoxia-inducible factors: mediators of cancer progression and targets for cancer therapy. Trends Pharmacol Sci.

[292] Qian DZ, Kachhap SK, Collis SJ, Verheul HM, Carducci MA, Atadja P, Pili R (2006). Class II histone deacetylases are associated with VHL-independent regulation of hypoxia-inducible factor 1 alpha. Cancer Res.

[293] Westerlund I, Shi Y, Toskas K, Fell SM, Li S, Surova O, Södersten E, Kogner P, Nyman U, Schlisio S, Holmberg J (2017). Combined epigenetic and differentiation-based treatment inhibits neuroblastoma tumor growth and links HIF2α to tumor suppression. Proc Natl Acad Sci U S A.

[294] Nakazawa MS, Eisinger-Mathason TS, Sadri N, Ochocki JD, Gade TP, Amin RK, Simon MC (2016). Epigenetic re-expression of HIF-2α suppresses soft tissue sarcoma growth. Nat Commun.

[295] Xiang Y, Stine ZE, Xia J, Lu Y, O'Connor RS, Altman BJ, Hsieh AL, Gouw AM, Thomas AG, Gao P (2015). Targeted inhibition of tumor-specific glutaminase diminishes cell-autonomous tumorigenesis. J Clin Invest.

[296] Weinberg SE, Chandel NS (2015). Targeting mitochondria metabolism for cancer therapy. Nat Chem Biol.

[297] Birsoy K, Possemato R, Lorbeer FK, Bayraktar EC, Thiru P, Yucel B, Wang T, Chen WW, Clish CB, Sabatini DM (2014). Metabolic determinants of cancer cell sensitivity to glucose limitation and biguanides. Nature.

[298] Lin Y, Wei X, Jian Z, Zhang X (2020). METTL3 expression is associated with glycolysis metabolism and sensitivity to glycolytic stress in hepatocellular carcinoma. Cancer Med.

[299] Chen H, Xiang Y, Yin Y, Peng J, Peng D, Li D, Kitazawa R, Tang Y, Yang J (2021). The m6A methyltransferase METTL3 regulates autophagy and sensitivity to cisplatin by targeting ATG5 in seminoma. Transl Androl Urol.

[300] Han S, Zhu L, Zhu Y, Meng Y, Li J, Song P, Yousafzai NA, Feng L, Chen M, Wang Y (2021). Targeting ATF4-dependent pro-survival autophagy to synergize glutaminolysis inhibition. Theranostics.

[301] Rau K, Rösner L, Rentmeister A (2019). Sequence-specific m(6)A demethylation in RNA by FTO fused to RCas9. RNA.

[302] Li J, Chen Z, Chen F, Xie G, Ling Y, Peng Y, Lin Y, Luo N, Chiang CM, Wang H (2020). Targeted mRNA demethylation using an engineered dCas13b-ALKBH5 fusion protein. Nucleic Acids Res.

[303] Pandey PR, Young KH, Kumar D, Jain N (2022). RNA-mediated immunotherapy regulating tumor immune microenvironment: next wave of cancer therapeutics. Mol Cancer.

[304] Einstein JM, Perelis M, Chaim IA, Meena JK, Nussbacher JK, Tankka AT, Yee BA, Li H, Madrigal AA, Neill NJ (2021). Inhibition of YTHDF2 triggers proteotoxic cell death in MYC-driven breast cancer. Mol Cell.

[305] Zhang F, Guo J, Zhang Z, Duan M, Wang G, Qian Y, Zhao H, Yang Z, Jiang X (2022). Application of engineered extracellular vesicles for targeted tumor therapy. J Biomed Sci.

[306] He G, Peng X, Wei S, Yang S, Li X, Huang M, Tang S, Jin H, Liu J, Zhang S (2022). Exosomes in the hypoxic TME: from release, uptake and biofunctions to clinical applications. Mol Cancer.

[307] Hou G, Zhao X, Li L, Yang Q, Liu X, Huang C, Lu R, Chen R, Wang Y, Jiang B, Yu J (2021). SUMOylation of YTHDF2 promotes mRNA degradation and cancer progression by increasing its binding affinity with m6A-modified mRNAs. Nucleic Acids Res.

[308] Tian QH, Zhang MF, Zeng JS, Luo RG, Wen Y, Chen J, Gan LG, Xiong JP (2019). METTL1 overexpression is correlated with poor prognosis and promotes hepatocellular carcinoma via PTEN. J Mol Med (Berl).

[309] Luo G, Xu W, Zhao Y, Jin S, Wang S, Liu Q, Chen X, Wang J, Dong F, Hu DN (2020). RNA m(6) A methylation regulates uveal melanoma cell proliferation, migration, and invasion by targeting c-Met. J Cell Physiol.

[310] Li J, Xie H, Ying Y, Chen H, Yan H, He L, Xu M, Xu X, Liang Z, Liu B (2020). YTHDF2 mediates the mRNA degradation of the tumor suppressors to induce AKT phosphorylation in N6-methyladenosine-dependent way in prostate cancer. Mol Cancer.

[311] Zhu H, Gan X, Jiang X, Diao S, Wu H, Hu J (2019). ALKBH5 inhibited autophagy of epithelial ovarian cancer through miR-7 and BCL-2. J Exp Clin Cancer Res.

[312] Cassim S, Pouyssegur J (2019). Tumor microenvironment: a metabolic player that shapes the immune response. Int J Mol Sci.

[313] Li H, Su Q, Li B, Lan L, Wang C, Li W, Wang G, Chen W, He Y, Zhang C (2020). High expression of WTAP leads to poor prognosis of gastric cancer by influencing tumour-associated T lymphocyte infiltration. J Cell Mol Med.

[314] Luo Y, Sun Y, Li L, Mao Y (2020). METTL3 may regulate testicular germ cell tumors through EMT and immune pathways. Cell Transplant.

[315] Song Z, Jia G, Ma P, Cang S (2021). Exosomal miR-4443 promotes cisplatin resistance in non-small cell lung carcinoma by regulating FSP1 m6A modification-mediated ferroptosis. Life Sci.

[316] Chao Y, Shang J, Ji W (2020). ALKBH5-m(6)A-FOXM1 signaling axis promotes proliferation and invasion of lung adenocarcinoma cells under intermittent hypoxia. Biochem Biophys Res Commun.

[317] Chen Y, Peng C, Chen J, Chen D, Yang B, He B, Hu W, Zhang Y, Liu H, Dai L (2019). WTAP facilitates progression of hepatocellular carcinoma via m6A-HuR-dependent epigenetic silencing of ETS1. Mol Cancer.

[318] Müller S, Bley N, Busch B, Glaß M, Lederer M, Misiak C, Fuchs T, Wedler A, Haase J, Bertoldo JB (2020). The oncofetal RNA-binding protein IGF2BP1 is a druggable, post-transcriptional super-enhancer of E2F-driven gene expression in cancer. Nucleic Acids Res.

[319] Guo J, Wu Y, Du J, Yang L, Chen W, Gong K, Dai J, Miao S, Jin D, Xi S (2018). Deregulation of UBE2C-mediated autophagy repression aggravates NSCLC progression. Oncogenesis.

[320] Zhao L, Kong X, Zhong W, Wang Y, Li P (2020). FTO accelerates ovarian cancer cell growth by promoting proliferation, inhibiting apoptosis, and activating autophagy. Pathol Res Pract.

[321] Yue C, Chen J, Li Z, Li L, Chen J, Guo Y (2020). microRNA-96 promotes occurrence and progression of colorectal cancer via regulation of the AMPKα2-FTO-m6A/MYC axis. J Exp Clin Cancer Res.

[322] Guo X, Li K, Jiang W, Hu Y, Xiao W, Huang Y, Feng Y, Pan Q, Wan R (2020). RNA demethylase ALKBH5 prevents pancreatic cancer progression by posttranscriptional activation of PER1 in an m6A-YTHDF2-dependent manner. Mol Cancer.

[323] Nie S, Zhang L, Liu J, Wan Y, Jiang Y, Yang J, Sun R, Ma X, Sun G, Meng H (2021). ALKBH5-HOXA10 loop-mediated JAK2 m6A demethylation and cisplatin resistance in epithelial ovarian cancer. J Exp Clin Cancer Res.

[324] Li J, Chen F, Peng Y, Lv Z, Lin X, Chen Z, Wang H (2020). N6-methyladenosine regulates the expression and secretion of TGFβ1 to affect the epithelial-mesenchymal transition of cancer cells. Cells.

[325] Yue B, Song C, Yang L, Cui R, Cheng X, Zhang Z, Zhao G (2019). METTL3-mediated N6-methyladenosine modification is critical for epithelial-mesenchymal transition and metastasis of gastric cancer. Mol Cancer.

[326] Lin X, Chai G, Wu Y, Li J, Chen F, Liu J, Luo G, Tauler J, Du J, Lin S (2019). RNA m(6)A methylation regulates the epithelial mesenchymal transition of cancer cells and translation of Snail. Nat Commun.

[327] Wanna-Udom S, Terashima M, Lyu H, Ishimura A, Takino T, Sakari M, Tsukahara T, Suzuki T (2020). The m6A methyltransferase METTL3 contributes to transforming growth factor-beta-induced epithelial-mesenchymal transition of lung cancer cells through the regulation of JUNB. Biochem Biophys Res Commun.

[328] Hua W, Zhao Y, Jin X, Yu D, He J, Xie D, Duan P (2018). METTL3 promotes ovarian carcinoma growth and invasion through the regulation of AXL translation and epithelial to mesenchymal transition. Gynecol Oncol.

[329] Chen Y, Wu R, Chen W, Liu Y, Liao X, Zeng B, Guo G, Lou F, Xiang Y, Wang Y, Wang X (2021). Curcumin prevents obesity by targeting TRAF4-induced ubiquitylation in m(6) A-dependent manner. EMBO Rep.

[330] Xu W, Xie S, Chen X, Pan S, Qian H, Zhu X (2021). Effects of quercetin on the efficacy of various chemotherapeutic drugs in cervical cancer cells. Drug Des Dev Ther.

[331] Lai W, Jia J, Yan B, Jiang Y, Shi Y, Chen L, Mao C, Liu X, Tang H, Gao M (2018). Baicalin hydrate inhibits cancer progression in nasopharyngeal carcinoma by affecting genome instability and splicing. Oncotarget.

[332] Sun K, Du Y, Hou Y, Zhao M, Li J, Du Y, Zhang L, Chen C, Yang H, Yan F, Su R (2021). Saikosaponin D exhibits anti-leukemic activity by targeting FTO/m(6)A signaling. Theranostics.

[333] Chen WW, Qi JW, Hang Y, Wu JX, Zhou XX, Chen JZ, Wang J, Wang HH (2020). Simvastatin is beneficial to lung cancer progression by inducing METTL3-induced m6A modification on EZH2 mRNA. Eur Rev Med Pharmacol Sci.

[334] Wei J, Yin Y, Zhou J, Chen H, Peng J, Yang J, Tang Y (2020). METTL3 potentiates resistance to cisplatin through m(6) A modification of TFAP2C in seminoma. J Cell Mol Med.

[335] Liu X, Su K, Sun X, Jiang Y, Wang L, Hu C, Zhang C, Lu M, Du X, Xing B (2021). Sec62 promotes stemness and chemoresistance of human colorectal cancer through activating Wnt/β-catenin pathway. J Exp Clin Cancer Res.

[336] Sun Y, Li S, Yu W, Zhao Z, Gao J, Chen C, Wei M, Liu T, Li L, Liu L (2020). N(6)-methyladenosine-dependent pri-miR-17-92 maturation suppresses PTEN/TMEM127 and promotes sensitivity to everolimus in gastric cancer. Cell Death Dis.

[337] Yankova E, Blackaby W, Albertella M, Rak J, De Braekeleer E, Tsagkogeorga G, Pilka ES, Aspris D, Leggate D, Hendrick AG (2021). Small-molecule inhibition of METTL3 as a strategy against myeloid leukaemia. Nature.

[338] Malacrida A, Rivara M, Di Domizio A, Cislaghi G, Miloso M, Zuliani V, Nicolini G (2020). 3D proteome-wide scale screening and activity evaluation of a new ALKBH5 inhibitor in U87 glioblastoma cell line. Bioorg Med Chem.

[339] Yan F, Al-Kali A, Zhang Z, Liu J, Pang J, Zhao N, He C, Litzow MR, Liu S (2018). A dynamic N(6)-methyladenosine methylome regulates intrinsic and acquired resistance to tyrosine kinase inhibitors. Cell Res.

[340] Wang SS, Lv Y, Xu XC, Zuo Y, Song Y, Wu GP, Lu PH, Zhang ZQ, Chen MB (2019). Triptonide inhibits human nasopharyngeal carcinoma cell growth via disrupting Lnc-RNA THOR-IGF2BP1 signaling. Cancer Lett.

[341] Zhang Y, Liu X, Yu M, Xu M, Xiao Y, Ma W, Huang L, Li X, Ye X (2020). Berberine inhibits proliferation and induces G0/G1 phase arrest in colorectal cancer cells by downregulating IGF2BP3. Life Sci.

[342] Mancarella C, Pasello M, Ventura S, Grilli A, Calzolari L, Toracchio L, Lollini PL, Donati DM, Picci P, Ferrari S, Scotlandi K (2018). Insulin-like growth factor 2 mRNA-binding protein 3 is a novel post-transcriptional regulator of ewing sarcoma malignancy. Clin Cancer Res.

[343] Dahlem C, Abuhaliema A, Kessler SM, Kröhler T, Zoller BGE, Chanda S, Wu Y, Both S, Müller F, Lepikhov K (2022). First small-molecule inhibitors targeting the RNA-binding protein IGF2BP2/IMP2 for cancer therapy. ACS Chem Biol.

[344] Ding N, You A, Tian W, Gu L, Deng D (2020). Chidamide increases the sensitivity of non-small cell lung cancer to crizotinib by decreasing c-MET mRNA methylation. Int J Biol Sci.

